# Design, Spectroscopy, and Assessment of Cholinesterase Inhibition and Antimicrobial Activities of Novel Coumarin–Thiadiazole Hybrids

**DOI:** 10.3390/ijms23116314

**Published:** 2022-06-05

**Authors:** Dariusz Karcz, Karolina Starzak, Ewa Ciszkowicz, Katarzyna Lecka-Szlachta, Daniel Kamiński, Bernadette Creaven, Anna Miłoś, Hollie Jenkins, Lidia Ślusarczyk, Arkadiusz Matwijczuk

**Affiliations:** 1 Department of Chemical Technology and Environmental Analytics (C1), Faculty of Chemical Engineering and Technology, Cracow University of Technology, 311-55 Kraków, Poland; karolina.starzak@pk.edu.pl; 2 Department of Biotechnology and Bioinformatics, Faculty of Chemistry, Rzeszow University of Technology, 35-959 Rzeszow, Poland; eciszkow@prz.edu.pl (E.C.); szlachta@prz.edu.pl (K.L.-S.); 3 Department of General and Coordination Chemistry and Crystallography, Institute of Chemical Sciences, Maria Curie-Sklodowska University in Lublin, 20-031 Lublin, Poland; dkami@umcs.pl; 4 School of Chemical and Pharmaceutical Sciences, Technological University Dublin, Central Quad, Grangegorman, D07 ADY7 Dublin, Ireland; Bernie.Creaven@tudublin.ie; 5 Department of Biotechnology and Bioinformatics, Faculty of Chemistry, Doctoral School of Engineering and Technical Sciences, Rzeszow University of Technology, 35-959 Rzeszow, Poland; d520@stud.prz.edu.pl; 6 Department of Applied Science, Technological University Dublin, Tallaght, D24 FKT9 Dublin, Ireland; x00108091@mytudublin.ie; 7 Department of Biophysics, University of Life Sciences in Lublin, 20-950 Lublin, Poland; lidia.slusarczyk@up.lublin.pl (L.Ś.); arkadiusz.matwijczuk@up.lublin.pl (A.M.)

**Keywords:** coumarin, thiadiazole, hybrids, cholinesterase inhibitors, neurodegeneration, antimicrobial activity, fluorescence imaging

## Abstract

A novel series of coumarin–thiadiazole hybrids, derived from substituted coumarin-3-carboxylic acids was isolated and fully characterized with the use of a number of spectroscopic techniques and XRD crystallography. Several of the novel compounds showed intensive fluorescence in the visible region, comparable to that of known coumarin-based fluorescence standards. Moreover, the new compounds were tested as potential antineurodegenerative agents via their ability to act as acetylcholinesterase (AChE) and butyrylcholinesterase (BuChE) inhibitors. Compared to the commercial standards, only a few compounds demonstrated moderate AChE and BuChE activities. Moreover, the novel derivatives were tested for their antimicrobial activity against a panel of pathogenic bacterial and fungal species. Their lack of activity and toxicity across a broad range of biochemical assays, together with the exceptional emission of some hybrid molecules, highlights the possible use of a number of the novel hybrids as potential fluorescence standards or fluorescence imaging agents.

## 1. Introduction

Coumarins and 1,3,4-thiadiazoles are heterocyclic moieties present in a large number of biologically active molecules of both natural and synthetic origin [1,2,3,4]. The relative ease of their isolation and structural modification, together with often extraordinary physiochemical characteristics, make these compounds an ever-growing source of novel fluorescent probes, sensors, dyes, and therapeutic agents [2,3,5,6,7]. The hybrids of coumarin derivatives are of particular interest in the field of medicinal chemistry as in most cases such compounds have shown greater antimicrobial activity than some standard drugs [6]. Furthermore, the thiadiazole derivatives are considered as useful intermediates in the synthesis of novel antimicrobial agents and especially those which may potentially help in addressing the issue of an increasing number of drug-resistant bacterial and invasive fungal species [4]. Moreover, an extended conjugation system of coumarin hybrids is often a key structural feature for the development of novel fluorescent dyes and fluorescence imaging agents [5].

Recently, a number of reports have evidenced a variety of newly synthesized molecules [8,9] including several coumarins and thiadiazoles as potent inhibitors of cholinesterase enzymes, and pointed at these compounds as potential novel classes of antineurodegenerative agents [10,11,12,13,14,15]. In addition, the presence of various electron-pair donating heteroatoms in coumarins and thiadiazoles enabled these compounds to act as effective chelators of a wide variety of transition metal ions [16,17]. Considering the current understanding of the role of metals in the progress of neurodegenerative disorders [18,19,20], these features of coumarins and thiadiazoles are particularly noteworthy. More specifically, the design of novel cholinesterase inhibitors, with the additional ability to act as chelators regulating the homeostasis of essential metals, may result in a new method for treatment of fatal neurodegenerative disorders such as Alzheimer’s or Parkinson’s diseases [18,19,20]. This type of therapeutic chelator may potentially prevent neurotoxic effects associated with an overexposure to metals and their accumulation within the brain tissues [19,20,21].

Coumarins and their biologically active transition metal complexes are of continuing interest to our group [22], though more recently we extended our scientific interest into 1,3,4-thiadiazole-based ligands and their transition metal complexes [23,24]. Moreover, we carried out in-depth studies on the excited state properties of various 1,3,4-thiadiazoles [25,26] and have shown that some known coumarin derivatives have potential as fluorescent probes sensitive to reactive chlorine species [27]. Our more recent effort relied on evidencing a number of 1,3,4-thiadiazoles and their Zn(II) complexes as potential antibacterial agents which synergistically enhance the therapeutic action of the commercial antibiotic Kanamycin [28]. Most recently, our studies focused on a series of novel coumarin–thiadiazole ligands together with their corresponding Zn(II) and Cu(II) complexes as potential acetylcholinesterase (AChE) inhibitors [29].

**Figure 1:**
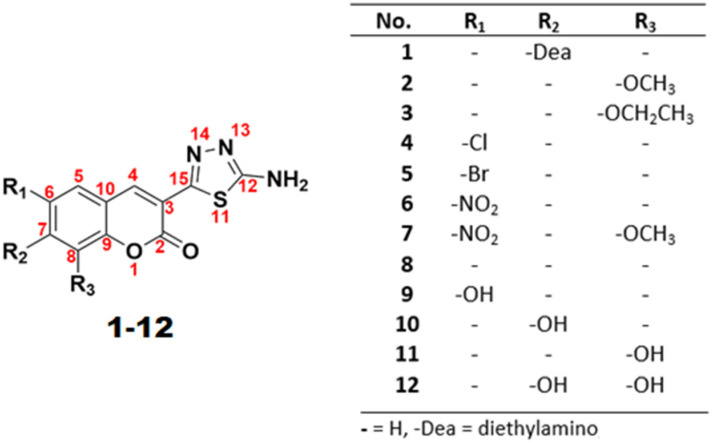
General structure of coumarin–thiadiazole hybrids **1-12** showing the numbering system of atoms and substituents present at the coumarin phenyl ring.

The promising results obtained to date together with the numerous reports on effective antimicrobial and antineurodegenerative potency of coumarins [16,30,31,32] and thiadiazoles [3,4,15,33] prompted us to continue our research on coumarin–thiadiazole hybrids and their biological activities. Therefore, in this work we report the isolation and structural characterization of another series of coumarin and 1,3,4-thiadiazole conjugates, wherein the link between coumarin and thiadiazole moieties is established at the C3 and C15 positions, respectively (Figure 1). A literature search revealed the previous isolation of only a limited number of similar hybrids with only a thiadiazole hybrid, derived from an unsubstituted coumarin 3-carboxylic acid, was reported to possess antimicrobial [34], analgesic, and antiprotolytic activities [35] and a structurally similar thiol-substituted derivative of that compound was reported to possess antitumor activity [36]. Regardless of the structural similarities, the synthetic protocols applied therein were notably different to those established by our group.

Thus, the main novelty of our current work is the isolation of novel coumarin–thiadiazole hybrids and their detailed structural characterization, carried out with use of various spectroscopic techniques. Secondly, all coumarin–thiadiazole hybrids isolated in this work were scrutinized for their ability to act as cholinesterase enzyme inhibitors and antimicrobial therapeutics and their cytotoxicity was assessed against a mammalian cell line.

## 2. Results and Discussion

### 2.1. Synthesis

Based on our previous work, the formation of an appropriate coumarin derivative was considered as the most convenient starting point for the isolation of coumarin–thiadiazole hybrids [29,37]. In our current work, we continued this approach by using the relatively easily accessible coumarin-3-carboxylic acids as precursors for the isolation of a novel series of coumarin–thiadiazole conjugates. In this new series, the coumarin moiety is directly linked at the C3 carbon with the thiadiazole ring (Figure 1).

**Figure 2:**
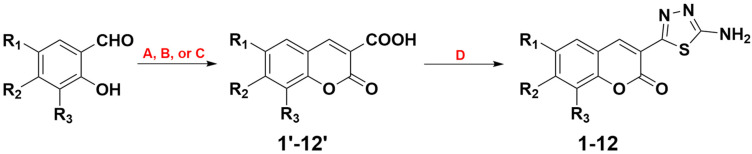
Synthetic pathway for the synthesis of coumarin–thiadiazole hybrids **1-12**: (**A**) diethyl malonate, piperidine, 40 °C; (**B**) Meldrum’s acid, piperidine, acetic acid, ethanol, reflux; (**C**) Meldrum’s acid, H_2_O, reflux; (**D**) POCl_3_, thiosemicarbazide, 75 °C.

The synthetic strategy applied involved two steps (Figure 2), the first step being the formation of the coumarin precursors **1′-12′** via the classical Knoevenagel condensation mechanism. Depending on the substituent present at the aromatic ring, alternative protocols were employed. In more detail, compounds **1′-3′** were obtained from the reaction between the appropriate salicylaldehyde and diethyl malonate with the formation of the ethyl ester and its subsequent alkaline hydrolysis [37], while Meldrum’s acid was used as a substrate for the isolation of compounds **4′-12′** [38,39].

**Figure 3:**
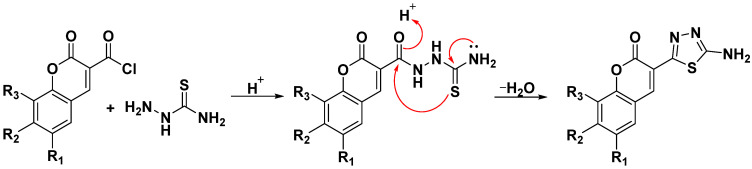
Formation of the coumarin–thiadiazole hybrids **1-12** from coumarin-3-carbonyl chloride intermediates.

The second synthetic step involved the transformation of coumarin carboxylic acids **1′-12′** into their corresponding 1,3,4-thiadiazole derivatives **1-12** (Figure 3). The procedure involved the POCl_3_-catalyzed acyl chloride formation and its subsequent reaction with thiosemicarbazide, followed by the in situ cyclization of the coumarin-3-(hydrazine-1-carbothioamide) intermediates (Figure 2).

Although in most cases the two-step formation of coumarin–thiadiazole hybrids was feasible, the synthesis of hydroxyl-substituted derivatives **9-12** proved more complicated. More specifically, the syntheses with use of the OH-substituted substrates **9′-12′** resulted in the isolation of impure corresponding coumarin–thiadiazole hybrids **9-12**. The undesirable side products originated most likely from the reactions between the unprotected phenolic groups and in situ generated acyl chloride. Therefore, protection of the phenolic groups in **9′-12′** was required. This issue was addressed by an acetylation of the phenolic groups in **9′-12′** with the use of acetic anhydride in the presence of concentrated H_2_SO_4_. The resulting acyl derivatives of **9′-12′** were used for the isolation of the corresponding acylated hybrids, which after deacetylation yielded the final OH-substituted hybrids **9-12** (Figure 3, Table 1).

### 2.2. XRD Crystallography

**Figure 4:**
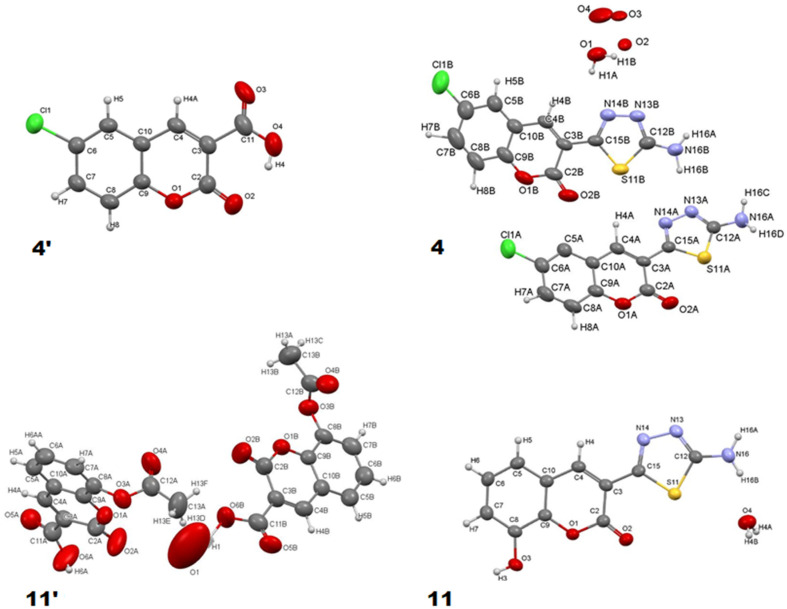
Crystal structures of coumarin–thiadiazole hybrids **4** and **11** and their respective coumarin-3-carboxylic acid precursors **4′** and **11′**.

Single crystals suitable for XRD analysis were grown from ethanolic solution upon slow evaporation of the solvent. Isolation of single crystals of coumarin–thiadiazole hybrids **4** and **11** as well as their respective coumarin-3-carboxylic acid precursors **4′** and **11′** allowed for a direct comparison of the crystal structures with the use of X-ray diffraction technique (Figure 4). All structures were solved by direct methods [40] and refined using SHELXL [41,42]. The refinement was based on squared structure factors (F^2^) for all reflections. All hydrogen atoms were located in idealized averaged geometrical positions. Details of all crystals measured are provided in Supplementary materials.

Examination of the crystal structure data revealed nearly identical bond lengths and valence angles for the coumarin moiety. The differences are observed only in the distance between C3 and C11 carbons which in **4′** is 1.469(3) Å, while in the corresponding hybrid **4** it is 1.459(5) Å, though it is worth emphasizing that the structure of **4′** was obtained from a low-quality crystal. Moreover, slight differences were observed in the case of the C3 and C11 (carboxylic) carbons in **4′** and C3 and C15 (thiadiazole) carbons in **4**. In more detail the bond linking the carboxylic group with the coumarin core in the acid **4′** seems shorter, but compared to the hybrid **4** it is burdened with greater error. Nevertheless, the difference observed might result from the presence of two carboxylic oxygen atoms which reduce the electron density at the C3-C11 bond in **4′** and this effect seems stronger compared to that caused by the thiadiazole ring in **4** and **11**.

Both coumarin-3-carboxylic acid molecules (**4′** and **11′**) have intramolecular hydrogen bonds between their carboxylic O4-H4 and the lactone O2 atoms which certainly affects the positioning of the IR bands (see Section 2.3). Molecules of coumarin 3-carboxylic acid **4′** are aligned parallel to the (101) plane in the crystal. The intermolecular interactions in **4′**, namely Cl-H8, H7-O1, and H5-O2, are relatively weak and result in brittleness of the crystal.

The crystal structure of precursor **11′** has two types of channels filled with disordered water molecules. To correctly solve the structure, a solvent mask and one water molecule O1 was added, which reduced the R_1_ value from ~11 to 7%. The independent part of the unit cell contains two molecules of **11′** which form hydrogen bonds with disordered water molecules from channels. This structure does not contain any internal hydrogen bonding. The neighboring molecules interact with one another by rather weak hydrogen bonds between O5A and H4B and H5B with the respective distances of 2.57 Å and 2.62 Å. Another weak but possible hydrogen bond may occur between O4A and H13B (2.63 Å). There is also an attractive interaction between the stacked **11′** molecules occurring via one electron pair of the O3A oxygen in the first molecule and the lactone ring of the second **11′** molecule where the distance between O3A and the ring is 3.073 Å.

The crystal lattice of the coumarin–thiadiazole hybrid **11** is rich in intermolecular hydrogen bonds. Both N13 and N14 nitrogen atoms of the thiadiazole ring form a hydrogen bond with the H3 hydrogen with the respective distances of 2.021 Å and 2.716 Å. The H16A hydrogen of the amine forms a hydrogen bond with the O3 oxygen (2.319 Å), while the H16B hydrogen forms a hydrogen bond with a water molecule with a distance of 2.048 Å. In this case, the lactone oxygen O2 does not participate in the formation of any hydrogen bonding, which is in line with the assignment of the relevant IR bands (see Section 2.3). Additionally, the molecules which are oriented parallel to one another in the crystal lattice interact through a number of π–π stacking interactions.

The crystal lattice in hybrid **4** differs significantly from that of **11** in terms of the number of hydrogen bonds formed by the amine group. In particular, there are several hydrogen bonds between the NH16-H16C…O1 (2.334 Å), NH16-H16D…N13A (2.718 Å), NH16-H16C…O2A (2.386 Å), N16B-H16A…N13B (2.152 Å), N16B-H16A…N13A (2.031 Å), and N16B-H16A…N14A (2.653 Å) atoms. Moreover, one of the water molecules present in the crystal of **11** forms a hydrogen bond with the thiadiazole nitrogen N14B (2.155 Å). The remaining water molecules are disordered in a channel extending along the *a* axis. The lactone oxygen O2B forms a relatively weak hydrogen bond with H4A hydrogen, but the O2A atom forms a strong hydrogen bond with the amine via the H16D hydrogen (2.385 Å). Although this interaction may potentially affect the lactone IR band positioning, no such effect was observed (see Section 2.3).

### 2.3. IR(ATR) Spectroscopy

**Figure 5:**
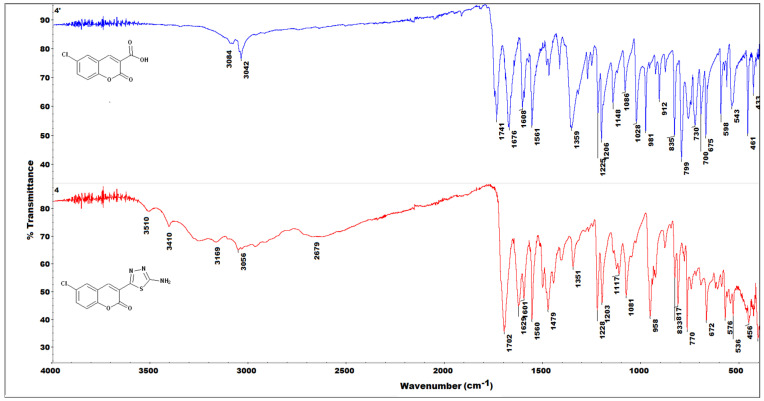
Comparison of the IR(ATR) spectra of coumarin-3-carboxylic acid **4′** (**top**) and the corresponding coumarin–thiadiazole hybrid **4** (**bottom**).

Representative examples of the IR spectra of the hybrids are given in Figure 5 and the remaining IR data are given in the Supplementary Materials (Figures S1–S11). The IR spectra of all coumarin 3-carboxylic acid intermediates **1′-12′** were similar to those reported in our previous studies [37,38,39,40,41,42,43]. More specifically, all free acids show two characteristic carbonyl C=O stretching bands originating from the carboxyl and the lactone moieties. Compared to an unsubstituted coumarin, where the lactone C=O stretch occurs at approximately 1700 cm^−1^ (Supplementary Material, Figure S12) in the coumarin carboxylic acids **1′-12′,** the lactone C=O bands are observed at somewhat lower wavenumbers as a result of an intramolecular hydrogen bond between the lactone oxygen and the carboxylic hydrogen (Figure 4). For the same reason, the carboxylic C=O band is positioned lower than usually [44], but its positioning is still higher than that of the lactone. The carboxylic C=O band appears in the region of 1710–1760 cm^−1^, while the lactone C=O stretch occupies the region of 1700–1650 cm^−1^ and in a few cases overlaps with the other bands appearing in this range.

Alteration of the carboxylic group to the thiadiazole derivative results in two significant changes observed in the IR spectra of the hybrid products **1-12**, namely the disappearance of the carboxylic C=O band and a notable positional change of the lactone C=O band. Initially, it was assumed that the lactone C=O band would shift towards a lower frequency, consistent with the hypothesis that increased conjugation occurring upon the formation of the thiadiazole ring notably lowers the energy of the coumarin lactone C=O stretching vibration. On the other hand, compared to the acids **1′-12′,** the increase in conjugation degree in **1-12** results from the formation of a 1,3,4-thiadiazole ring, which incorporates a large number of strongly electronegative atoms. As such, it also introduces a notable electron withdrawing effect and, as a consequence, increased energy of the coumarin lactone C=O vibration [44]. This effect is in line with the slight downfield shifts of **^1^**H-NMR signals observed (see Section 2.4). Moreover, the thiadiazole ring formation is accompanied by a disappearance of the intramolecular hydrogen bond (see Section 2.2, Figure 4, and crystallographic data in the Supplementary Materials), which shifts the lactone C=O band to a wavenumber similar to that of the unsubstituted coumarin (approximately 1700 cm^−1^). Trends similar to those observed here for this type of complex have previously been shown in theoretical studies [43,45].

Further evidence for the formation of the thiadiazole ring in **1-12** was the sequence of sharp bands at 1600 cm^−1^ cm and 1500–1450 cm^−1^, characteristic of the thiadiazole C=N stretching vibrations [29]. Moreover, most hybrids revealed the characteristic amine N-H bands at approximately 3300 cm^−1^. These bands often overlapped with several other bands observed through the whole series of hybrids **1-12** originating from the specific substituents present at the coumarin phenyl ring.

### 2.4. NMR Spectroscopy

Transformation of the carboxylic acids **1′-12′** into the corresponding coumarin–thiadiazole hybrids **1-12** is accompanied by a number of differences observed in the ^1^H-NMR spectra. Most importantly, the highly deshielded and relatively broad singlet originating from the carboxylic hydrogen present in the spectra of acids **1′-12′** is replaced with a more intense and sharp singlet of the primary amine in coumarin–thiadiazole hybrids **1-12,** the latter signal being present in all spectra at ca. 7.40 ppm. In a few of the spectra this latter peak overlaps with those of the coumarin phenyl, though its assignment remains relatively easy based on the relative integral values.

**Figure 6:**
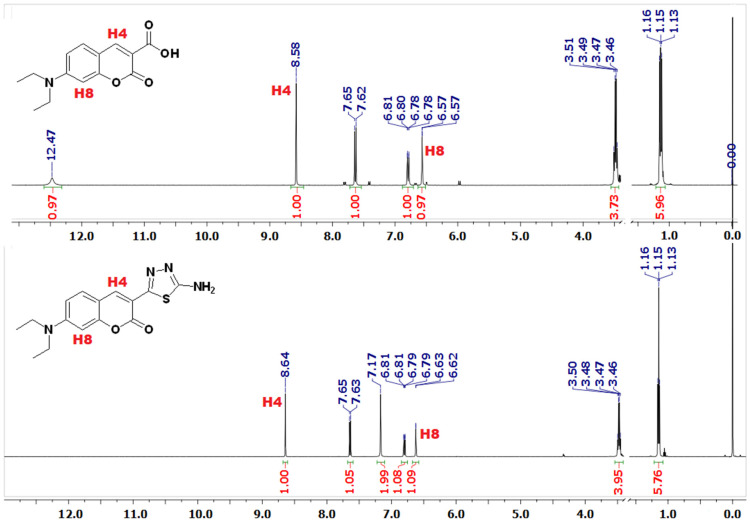
Comparison of the ^1^H-NMR (DMSO_d6_) spectra of coumarin-3-carboxylic acid **1′** (**top**) and the corresponding coumarin–thiadiazole hybrid **1** (**bottom**). The most informative signals, namely the hydrogens H4 and H8, are marked in red. For better clarity, the residual solvent and water peaks were omitted.

As is seen in the representative example (Figure 6), in all **^1^**H-NMR spectra the coumarin vinyl hydrogen H4 gives rise to a singlet peak. The positioning between the phenyl ring and the strong electron withdrawing carbonyl groups results in a characteristic strong deshielding of this proton and its appearance is at ca. 8.5 ppm. Interestingly, in hybrids **1-12** this peak is shifted more downfield compared to the spectra of corresponding acids **1′-12′**. Moreover, a similar slight downfield shift takes place in the case of the signal arising from the proton H8. The slight downfield shifts of H4 and H8 peaks support the hypothesis that the formation of a thiadiazole ring results in an electron withdrawal from the coumarin lactone ring, which, in turn gives rise to a number of characteristic features observed, not only in the NMR spectra, but also in the IR spectra of the coumarin–thiadiazole hybrids obtained (see Section 2.3). The **^1^**H-NMR spectra of the remaining hybrids **2-12** are provided in the Supplementary Materials (Figures S13–S21).

Due to the relatively low solubility of hybrids **1-12** in the commonly used deuterated solvents, efforts at the acquisition of good quality ^13^C-NMR spectra remained unsuccessful. In most cases, the ^13^C-NMR spectra lacked quaternary carbon peaks, for which the long T2 relaxation time is a well-known issue [44].

### 2.5. UV-Vis Spectroscopy

**Figure 7:**
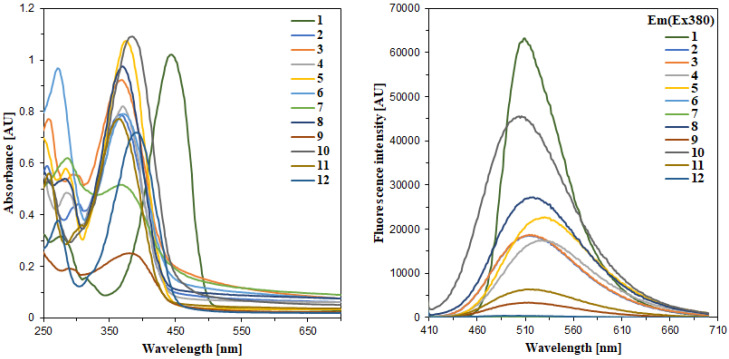
Electronic absorption (**left**) and fluorescence emission spectra (**right**) of coumarin–thiadiazole hybrids **1-12** recorded in methanol. The absorption spectra were recorded at a concentration of 0.2 mM, except **1**, which was recorded at 0.04 mM. The fluorescence spectra were recorded at λ_ex_ = 380 nm and a concentration of 0.01 mM, except **1,** which was recorded at λ_ex_ = 420 nm and 0.002 mM, respectively.

The UV-Vis spectra of hybrids **1-12** were recorded in 0.2 mM methanolic solutions, except for compound **1,** which was recorded at a concentration of 0.04 mM (Figure 7). All spectra demonstrated two distinct bands with the higher energy band observed at approximately 255–290 nm and the lower energy one at about 360–390 nm (Table 1). The higher energy band was assigned to the phenyl ring [28], while the lower energy one is characteristic of coumarin–thiadiazoles or other coumarin conjugates [29]. In most cases the lower energy band was of much higher intensity, except for the -NO_2_-substituted compounds **6** and **7**, in which the higher energy band was more intense. Undoubtedly, all bands observed originate from the allowed π–π* transitions and the differences in positioning of these bands along the series result from the substituents present at the coumarin phenyl ring.

Compared to all the remaining compounds in the series, the electronic absorption characteristics of compound **1** were notably different. In particular, its higher energy band was of low intensity, while the low energy band was intense and additional dilution (down to 0.04 mM) was necessary in order to compare the spectra (Figure 7). Moreover, compared to hybrids **2-12**, the maximum wavelength of the low energy band in **1** was notably shifted to the visible region and was observed at 445 nm.

### 2.6. Fluorescence Spectroscopy

In order to avoid undesirable reabsorption processes, the steady-state fluorescence spectra of hybrids **1-12** were recorded in 0.01 mM methanolic solutions, except for compound **1**, which was diluted down to 0.002 mM. Moreover, due to its notable difference in positioning of the absorption maximum in **1**, the excitation wavelength was set to 420 nm, while the remaining hybrids **2-12** were excited at 380 nm (Figure 7).

Each coumarin–thiadiazole hybrid revealed a single emission band. Depending on the substituents present at the coumarin phenyl ring, the emission maxima ranged from 530 to 500 nm. Moreover, significant differences in the intensities of the emission bands were observed along the series. The lowest energy emission was observed in the halogen-substituted hybrids **4** and **5** (524 and 530 nm, respectively). The unsubstituted derivative **8** displayed maximum emission at 518 nm, while the 8-substituted hybrids **2**, **3** and **11**, together with the 6-hydroxyl-substituted compound **9**, emitted at about 513 nm. The 7-substituted hybrids **1** and **10** have their emission maxima at lower wavelengths (509 and 504 nm, respectively), while the 7,8-dihydroxy derivative **12** gave the highest energy emission (501 nm) (Table 1).

**Figure 8:**
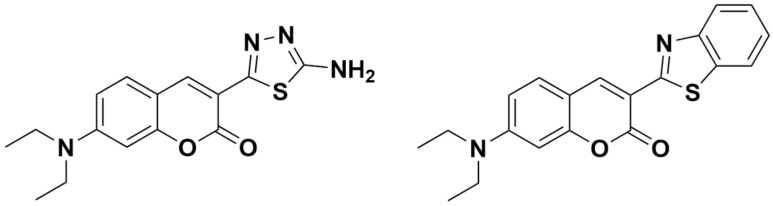
Comparison of structures of highly fluorescent coumarin–thiadiazole hybrid **1** (**left**) and coumarin-derived fluorescence standard **Coumarin 6** (**Right**).

The intensities of the emission bands observed in **1-12** were even more substituent-dependent. In 7-diethylamino-substituted hybrid **1,** the intensity was the highest in the series. Even though the concentration of **1** was notably lower, its fluorescence intensity was still the highest in the series, with 7-hydroxyl-substituted compound **10** demonstrating the second highest fluorescence intensity in the series (Figure 7). Apparently the substitution at the C7 carbon of the coumarin nucleus with a strong ring-activating moiety results in an increased fluorescence quantum yield, consistent with the structural features of known coumarin-based fluorescent standard Coumarin 6 (Figure 8) [46,47]. The intensities of the 6- and 8-substituted hybrids (**2-5**) and unsubstituted derivative **8** were comparable to one another but notably lower compared to that of **10**. Clearly, the weak ring-deactivating substituents or weak activating moieties present at the respective C6 and C8 carbons are responsible for such a trend. In turn, the presence of a strongly activating substituent such as the hydroxyl at the C6 or C8 carbon results in a notable decrease in fluorescence intensity of the respective hybrids **9** and **11**. Interestingly, an even more notable decrease in the fluorescence intensity is observed in 7,8-dihydroxyl derivative **12**, regardless of the presence of an OH-group at the C7 carbon. The relatively weak fluorescence intensity of the hydroxyl-substituted derivatives may result from the intermolecular interactions such as the hydrogen bonding, which is known to favor internal conversion as the main relaxation mechanism in polar solvents. More detailed information on the solvent dependence of the excited state properties of hybrids **1-12** would require extended and time-consuming experimentation involving the use of various solvents. Such experiments will be considered as future work. The fluorescence intensity demonstrated by nitro-substituted hybrids **6** and **7** was negligible, leading to the conclusion that the strong ring deactivating substituents at the C6 carbon result in quenching the fluorescence of the whole compound. Considering the fact that the nitro group may often act as fluorescence quencher, compounds **6** and **7** were considered non-fluorescent.

### 2.7. AChE and BuChE Inhibition Activity

Compared to the tacrine control, all hybrids tested show only a moderate ability to inhibit both cholinesterase enzymes. In general, the IC_50_ values determined for **1-12** are comparable with those obtained previously from hybrids in which the thiadiazole moiety was attached to coumarin via the C8 carbon of the coumarin phenyl ring [29]. Nonetheless, it is worth noticing that the two most active anti-AChE compounds (**3** and **11**) have electron-donating substituents at the C8 coumarin carbon [48], while in the case of the anti-BuChE assay compounds **2** and **11** with electron-donating substituents at C8 carbon and compounds **4** and **6** with electron-withdrawing groups at C6 carbon demonstrated the highest activity in the series. It is highly likely that the relatively low activities result from the lack of flexibility of the hybrids. The rigidity of structural cores in hybrids **1-12** may prevent them from adopting the conformation necessary for reaching and docking in the enzyme-binding site [30]. This in turn raises the idea of introducing a linker between the coumarin and thiadiazole pharmacophores which would provide the required flexibility, similar to that reported in ensaculin [49]. The results obtained are presented in Table 2.

### 2.8. Antibacterial Activity

Minimum inhibitory and minimum bactericidal concentrations (MIC and MBC, respectively) were determined in order to evaluate the antibacterial activity of hybrids **1-12** (Table 3). The saturated solution of each compound was used as a stock and hence each compound was tested at different concentration ranges. Compared to Gram-positive strains (*S. aureus*, *S. epidermidis*), the Gram-negative species (*E. coli*, *P. aeruginosa*) demonstrated higher resistance against the tested compounds. Only two compounds, namely **1** and **6**, were able to moderately inhibit the growth of *P. aeruginosa* with MIC values 1.79 mg/mL and 0.25 mg/mL, respectively. The lowest concentrations needed to inhibit bacterial growth were observed for **6** and **11** against *S. epidermidis* ATCC 12228 with, respectively, 0.03 mg/mL and 0.01 mg/mL. Compound **12** showed no antibacterial properties against any strains tested. Interestingly, the MBC values were identified for **7** out of **12** compounds and only against *S. epidermidis* ATCC 12228 (non-biofilm formatting strain).

Overall, the results obtained revealed no significant antibacterial activity of hybrids **1-12** against the bacterial strains tested. All compounds were notably less active compared to the control drugs. Moreover, considering the fact that there are numerous other coumarin and thiadiazole derivatives reported to possess significantly higher antibacterial activities, the series of coumarin–thiadiazole hybrids obtained in this work is considered inactive.

### 2.9. Antifungal Activity

Antifungal activity was tested at five different concentrations, namely 16, 32, 64, 128, and 256 μg/mL. Most compounds did not demonstrate any significant antifungal effect against the species tested. The MIC values determined were significantly higher compared to that of the Amphotericin B control (Table 4) and only the *A. fumigatus* showed a limited susceptibility to a few compounds from the series. Despite the overall poor antifungal activity of whole series, the exposure of fungal strains to hybrids **1-12** resulted in a notable prolongation of their growth time, which in a number of cases was approximately 30 h.

### 2.10. Determination of Cytotoxicity

A number of previous studies on related complexes had shown that metal complexes of coumarins were active against immortalized cell lines and it was thought that the increased solubility of the hybrids tested here may give rise to an increase in cytotoxicity against mammalian-derived cells. Cytotoxicity testing for the compounds was carried out over a range of 0 to 500 μM and compared to the control mitoxantrone, which the clinical agent used for breast cancer and is the positive control for the MCF-7 breast cancer-derived cell line. None of the compounds showed any activity, even at the highest concentration tested. Therefore, the hybrids tested were essentially non-cytotoxic and this lack of cytotoxicity does highlight the potential of a select number of the hybrids as imaging agents.

## 3. Experimental Section

### 3.1. Materials and Methods

All chemicals used for the syntheses and for biological testing were of reagent grade or higher. The substituted salicylaldehydes, thiosemicarbazide, POCl_3_, acetic anhydride, and DMSO-d_6_, were purchased from Aldrich (Darmstadt, Germany). Concentrated HCl and solid NaOH were purchased from ChemPur (Piekary Śląskie, Poland). Methanol and metal salts were purchased from Avantor (Gliwice, Poland). Ethanol was purchased from Honeywell (Offenbach, Germany) All solvents were of 99% purity (HPLC grade) or higher. Compounds used for the antimicrobial studies, namely dimethyl sulfoxide (99%), Mueller Hinton Broth (MHB), and Mueller Hinton Agar (MHA), were purchased from Merck, Darmstadt, Germany. The commercially available antibiotics chloramphenicol and kanamycin (Carl ROTH, Germany), and gentamicin (Aldrich, Germany) were used as reference standards. All reagents and bacterial cultures were prepared using Laminar Flow Cabinet ESCO Airstream.

The NMR spectra were acquired in d_6_-DMSO on a Bruker Avance III spectrometer (500 MHz). A Shimadzu IR Spirit FT-IR apparatus equipped with the QATR-S ATR adapter was used for the recording of IR Spectra. All electronic absorption and steady-state fluorescence spectra were recorded on a Tecan Infinite 200 microplate reader (Tecan Austria GmbH, Grödig/Salzburg, Austria) using 96-well plates. Melting point values were recorded on a Stuart SMP20 apparatus within the range of 25–300 °C and were uncorrected. The single crystal X-ray diffraction data were collected at 293 K on a SuperNova diffractometer (Agilent, Santa Clara, CA, USA) with CuKa radiation for all measured crystals. Cell refinement and data collection as well as data reduction and analysis were performed with CRYSALIS^PRO^ software (Rigaku, Austin, TX, USA). Structures were solved with the use of ShelXT [41] and refined with the SHELXL−2014 [42] included in Olex2 software [40]. All non-hydrogen atoms were refined with anisotropic displacement parameters. Hydrogen atoms attached to carbon atoms were added to the structure model at geometrically idealized positions and refined as riding atoms with Uiso(H) = 1.2Ueq (CH and CH_2_) or Uiso(H) = 1.5Ueq (CH_3_). The measurement information is presented in the Supplementary Materials.

The AChE and BuChE inhibition assays were performed on 96-well plates using a Tecan Infinite 200 microplate reader (Tecan Austria GmbH, Grödig/Salzburg, Austria). Acetylcholinesterase (E.C. 3.1.1.7) from *Electrophorus electricus*, acetylthiocholine iodide, butyrylcholinesterase (EC 3.1.1.8) from equine serum, butyrylthiocholine iodide, tacrine hydrochloride hydrate, and Ellman’s reagent were purchased from Sigma-Aldrich (St. Louis, MO, USA). Buffer of pH 8 was obtained from Honeywell (Charlotte, NC, USA).

The antibacterial activities were determined against Gram-positive (*Staphylococcus aureus* ATCC 6538, *Staphylococcus epidermidis* ATCC 12228, non-biofilm-formation strain, and *Staphylococcus epidermidis* ATCC 35984, high-biofilm-forming strain) and Gram-negative (*Escherichia coli* ATCC 10536 and *Pseudomonas aeruginosa* ATCC 15442) bacterial strains, obtained from the Department of Biotechnology and Bioinformatics, Faculty of Chemistry, Rzeszow University of Technology.

The antifungal activities were assessed against *Candida parapsilosis*, *Saccharomyces cerevisiae*, *Aspergillus flavus*, *Aspergillus fumigatus*, and *Fusarium oxysporum* species. Sabouraud agar dextrose (BTL, Łódź, Poland) was used for the fungal cell growth. The analyses were carried out using the Bioscreen C apparatus (Labsystem, Helsinki, Finland).

### 3.2. Synthesis of a Series of Substituted Coumarin-3-Carboxylic Acids (***1-12**′*)

Depending on the substituents present at the salicylaldehyde substrate, three various methodologies were applied for the syntheses of coumarin-3-carboxylic acids **1′-12′** [37,38,39]. Since the coumarin carboxylic acids **1′-12′** are known compounds, their detailed spectroscopic characterization was omitted. The identities and purities of these intermediates were confirmed by comparison of the FT-IR spectra (Figures S1–S12) and the melting point (Mp) values recorded with those available in literature [37,38,39].

#### 3.2.1. Synthesis of Coumarin Carboxylic Acids **1**′–**5**′, and **8**′ (Method A)

An equimolar amount of the appropriate salicylaldehyde and diethyl malonate was dissolved in ethanol in a 50 mL flask and few drops of piperidine were added. The mixture was stirred under reflux for 6 h and then poured onto water with ice. The precipitated coumarin-3-carboxylate ethyl ester was filtered off and dried in air. The ester was then suspended in water–ethanol (3:2 *v*/*v*) and solid NaOH was added. The mixture was refluxed for 30 min and then it was poured onto ice cold 20% HCl. The solid formed was filtered off and recrystalized from ethanol.

#### 3.2.2. Synthesis of Coumarin Carboxylic Acids **6**′ and **7**′ (Method B)

An equimolar amount of the appropriate salicylaldehyde and Meldrum’s acid were suspended in water and refluxed for 3–6 h. The mixture was then cooled down to ambient temperature and the solid was filtered off and recrystalized from ethanol.

#### 3.2.3. Synthesis of Coumarin Carboxylic Acids **9**′–**12**′ (Method C)

An equimolar amount of the appropriate salicylaldehyde and Meldrum’s acid were dissolved in ethanol and a few drops of piperidine and acetic acid were added. The mixture was then refluxed for 6 h and then poured onto ice. The diluted NaOH was used to adjust the pH in order to precipitate the crude product, which was then filtered off and recrystalized from ethanol.

### 3.3. Synthesis of Coumarin–Thiadiazole Hybrids (***1**–**8***)

Coumarin–thiadiazole hybrids **1-8** were obtained according to a previously published procedure [29] with minor modifications. Typically, the appropriate substituted coumarin 3-carboxylic acid was suspended in POCl_3_ and stirred at room temperature for 20 min. An equivalent amount of thiosemicarbazide was then added and the mixture was heated up and stirred at 80 °C for 3 h. After that time, the mixture was cooled down to 40 °C and the excess POCl_3_ was quenched by the slow addition of small aliquots of water. The mixture was then refluxed at 105 °C for another 3 h, cooled down and the pH was brought to approximately 7.5 with a diluted NaOH solution. The precipitate formed was filtered off, dried and recrystalized from ethanol.

(**1**) Yield: 95%; C_15_H_16_N_4_O_2_S (316.38 g/mol); M.P.: 234–236 °C; ^1^H-NMR (DMSO): δ = 8.64 ppm (s, 1H, H4), 7.64 (d, 1H, H5, *J* = 9.0 Hz), 7.17 (s, 2H, H17, (-NH_2_)), 7.05 (dd, 1H, H6, *J*_1_ = 9.0, *J*_2_ = 2.5 Hz), 6.63 (d, 1H, H8, *J*_1_ = 2.5 Hz); IR (ATR): 3379, 3284, 3083, 2966, 1689, 1610, 1577, 1497, 1414, 1356, 1261, 1200, 1186, 1125, 1073, 1014, 939, 819, 793, 768, 672, 473 cm^−1^; UV-Vis (MeOH): λ_max1_ = 285 nm, λ_max2_ = 445 nm; fluorescence (MeOH): λ_Em(Ex420)_ = 509 nm

(**2**) Yield: 87%; C_12_H_9_N_3_O_3_S (275.28 g/mol); M.P.: >300 °C; ^1^H-NMR (DMSO): δ = 8.84 ppm (s, 1H, H4), 7.48 (dd, 1H, H5, *J*_1_ = 6.1, *J*_2_ = 2.6 Hz), 7.42 (s, 2H, H17, (-NH_2_)), 7.37 (m, 2H, H6 and H7), 3.94 (s, 3H, (-OCH_3_)); IR (ATR): 3047, 3272, 3040, 1700, 1642, 1600, 1572, 1445, 1395, 1363, 1127, 1225, 1183, 1099, 1009, 949, 784, 764, 723, 533, 474 cm^−1^; UV-Vis (MeOH): λ_max1_ = 307 nm, λ_max2_ = 367 nm; fluorescence (MeOH): λ_Em(Ex380)_ = 513 nm

(**3**) Yield: 87%; C_13_H_11_N_3_O_3_S (289.31 g/mol); M.P.: >300 °C; ^1^H-NMR (DMSO): δ = 8.83 ppm (s, 1H, H4), 7.46 (dd, 1H, H5, *J* = 2.5 Hz), 7.42 (s, 2H, H17, (-NH_2_)), 7.35 (m, 2H, H6 and H7), 4.21 (q, 2H, (-OCH_2_-), *J* = 6.9 Hz), 1.42 (t, 3H, (-CH_3_) *J* = 6.9 Hz); IR (ATR): 3047, 3272, 3040, 1700, 1624, 1600, 1572, 1445, 1395, 1363, 1277, 1225, 1183, 1099, 1009, 949, 784, 764, 723, 533, 474 cm^−1^; UV-Vis (MeOH): λ_max1_ = 306 nm, λ_max_ = 368 nm; fluorescence (MeOH): λ_Em(Ex380)_ = 511 nm

(**4**) Yield: 82%; C_11_H_6_ClN_3_O_2_S (324.15 g/mol); M.P.: >300 °C; ^1^H-NMR (DMSO): δ = 8.84 ppm (s, 1H, H4), 8.08 (d, 1H, H5, *J* = 2.6 Hz), 7.71 (dd, 1H, H7, *J*_1_ = 8.9, *J*_2_ = 2.6 Hz), 7.56 (s, 2H, H17, (-NH_2_)), 7.54 (d, 1H, H8, *J* = 8.9 Hz); IR (ATR): cm^−1^; UV-Vis (MeOH): λ_max1_ = 284 nm, λ_max2_ = 370 nm; fluorescence (MeOH): λ_Em(Ex380)_ = 524 nm

(**5**) Yield: 93%; C_11_H_6_BrN_3_O_2_S (279.70 g/mol); M.P.: >300 °C; ^1^H-NMR (DMSO): δ = 8.83 ppm (s, 1H, H4), 8.21 (d, 1H, H5, *J* = 2.4 Hz), 7.81 (dd, 1H, H7, *J*_1_ = 8.9, *J*_2_ = 2.4 Hz), 7.57 (s, 2H, H17, (-NH_2_)), 7.47 (d, 1H, H8, *J* = 8.9 Hz); IR (ATR): 3524, 3411, 3289, 3061, 1701, 1624, 1598, 1556, 1504, 1488, 1400, 1228, 1206, 1133, 1068, 1028, 960, 925, 830, 814, 784, 770, 666, 576, 514, 454 cm^−1^; UV-Vis (MeOH): λ_max1_ = 284 nm, λ_max2_ = 374 nm; fluorescence (MeOH): λ_Em(Ex380)_ = 530 nm

(**6**) Yield: 87%; C_11_H_6_N_4_O_4_S (290.25 g/mol); M.P.: 294–296 °C; ^1^H-NMR (DMSO): δ = 9.02 ppm (s, 1H, H4), 8.94 (d, 1H, H5, *J* = 2.7 Hz), 8.44 (dd, 1H, H7, *J*_1_ = 9.2, *J*_2_ = 2.7 Hz), 7.72 (d, 1H, H8, *J* = 9.2 Hz), 7.58 (s, 2H, H17, (-NH_2_)); IR (ATR): 3302, 3080, 1707, 1618, 1603, 1574, 1525, 1472, 1422, 1342, 1236, 1135, 1030, 958, 833, 786, 772, 750, 673, 543, 465, 444 cm^−1^; UV-Vis (MeOH): λ_max1_ = 272 nm, λ_max2_ = 371 nm

(**7**) Yield: 81%; C_12_H_8_N_4_O_5_S (320.28 g/mol); M.P.: 292–294 °C; ^1^H-NMR (DMSO): δ = 8.99 ppm (s, 1H, H4), 8.54 (d, 1H, H5, *J* = 2.4 Hz), 8.03 (d, 1H, H7, *J* = 2.4), 7.55 (s, 2H, H17, (-NH_2_)), 4.07 (s, 3H, (-OCH_3_)); IR (ATR): 3473, 3370, 3101, 3036, 1710, 1606, 1525, 1479, 1451, 1369, 1344, 1298, 1239, 1098, 948, 874, 775, 739, 561 cm^−1^; UV-Vis (MeOH): λ_max1_ = 286 nm, λ_max2_ = 375 nm

(**8**) Yield: 82%; C_11_H_7_N_3_O_2_S (245.26 g/mol); M.P.: >300 °C; ^1^H-NMR (DMSO): δ = 8.99 ppm (s, 1H, H4), 7.94 (d, 1H, H5, *J* = 7.8 Hz), 7.68 (t, 1H, H6, *J* = 7.8 Hz), 7.50 (d, 1H, H8, *J* = 8.3 Hz), 7.43 (m, 3H, (-NH_2_ and H7)); IR (ATR): 3047, 3269, 3161, 3053, 1700, 1625, 1606, 1564, 1464, 1436, 1365, 1205, 1124, 1032, 955, 924, 771, 751, 644, 570, 461 cm^−1^; UV-Vis (MeOH): λ_max1_ = 282 nm, λ_max2_ = 370 nm; fluorescence (MeOH): λ_Em(Ex380)_ = 518 nm

### 3.4. Synthesis of Coumarin–Thiadiazole Hybrids (***9**-**12***)

The appropriate hydroxyl-substituted coumarin 3-carboxylic acid **9′-12′** was suspended in acetic anhydride and two drops of concentrated H_2_SO_4_ were added. The mixture was stirred for 10 min at room temperature and then heated up to 60 °C for another 10 min. The mixture was then cooled down to room temperature and poured onto ice water. The precipitate formed was filtered off, washed with cold methanol and dried in an oven at 80 °C, yielding the acetoxy-derived coumarin carboxylic acid intermediate. The dry acetoxy-derivative was then suspended in POCl_3_ and stirred at room temperature for 20 min. An equivalent of thiosemicarbazide was then added and the mixture heated up and stirred at 80 °C for 3 h. After that time, the mixture was cooled down to 40 °C and the excess POCl_3_ was quenched by the slow addition of small aliquots of water. The mixture was then refluxed at 105 °C for another 3 h, cooled down and the pH was brought to approximately 7.5 with the diluted NaOH solution. The precipitate formed was filtered off and dried in an oven. Finally, the acetoxy-derived hybrid was subjected to deacetylation by refluxing in a 2:1 (*v*/*v*) mixture of concentrated HCl and ethanol for 6 h. After that time, the mixture was cooled down and the pH was brought to ~7 with the use of the diluted NaOH. The crude product was then filtered off, dried and recrystalized from ethanol.

(**9**) Yield: 84%; C_11_H_7_N_3_O_3_S (261.26 g/mol); M.P.: >300 °C; ^1^H-NMR (DMSO): δ = 8.78 ppm (s, 1H, H4), 7.94 (m, 3H, (-NH_2_ and H8)), 7.23 (d, 1H, H5, *J* = 2.8 Hz), 7.11 (dd, 1H, H7, *J*_1_ = 8.9, *J*_2_ = 2.8 Hz); IR (ATR): 3538, 3107, 2880, 2755, 1700, 1599, 1574, 1472, 1436, 1373, 1294, 1226, 1203, 1089, 1022, 950, 838, 798, 736, 709, 633, 539, 462 cm^−1^; UV-Vis (MeOH): λ_max1_ = 290 nm, λ_max2_ = 378 nm; fluorescence (MeOH): λ_Em(Ex380)_ = 514 nm

(**10**) Yield: 96%; C_11_H_7_N_3_O_3_S (261.26 g/mol); M.P.: >300 °C; ^1^H-NMR (DMSO): δ = 8.74 ppm (s, 1H, H4), 7.73 (d, 1H, H5, *J* = 8.6 Hz), 7.29 (s, 2H, H17, (-NH_2_), 6.84 (dd, 1H, H6, *J*_1_ = 8.6, *J*_2_ = 2.1 Hz), 6.77 (d, 1H, H8, *J* = 2.1 Hz); IR (ATR): 3511, 3411, 3326, 3293, 3216, 3045, 1687, 1594, 1508, 1449, 1375, 1332, 1255, 1203, 1126, 950, 780, 767, 741, 642, 510 cm^−1^; UV-Vis (MeOH): λ_max1_ = 254 nm, λ_max2_ = 385 nm; fluorescence (MeOH): λ_Em(Ex380)_ = 504 nm

(**11**) Yield: 78%; C_11_H_7_N_3_O_3_S (261.26 g/mol); M.P.: >300 °C; ^1^H-NMR (DMSO): δ = 10.40 ppm (s, 1H, (-OH)), 8.80 ppm (s, 1H, H4), 7.42 (s, 2H, H17, (-NH_2_), 7.35 (dd, 1H, H5, *J*_1_ = 7.8, *J*_2_ = 1.5 Hz), 7.21 (t, 1H, H6, *J* = 7.9 Hz), 7.15 (dd, 1H, H7, *J*_1_ = 8.0, *J*_2_ = 1.5 Hz); IR (ATR): 3426, 3385, 3325, 3246, 3166, 1701, 1604, 1573, 1470, 1368, 1295, 1225, 1205, 1168, 1078, 1064, 947, 788, 767, 736, 711, 634, 602, 539, 467 cm^−1^; UV-Vis (MeOH): λ_max1_ = 258 nm, λ_max2_ = 364 nm; fluorescence (MeOH): λ_Em(Ex380)_ = 513 nm

(**12**) Yield: 75%; C_11_H_7_N_3_O_4_S (277.25 g/mol); M.P.: >300 °C; ^1^H-NMR (DMSO): δ = 8.82 ppm (s, 1H, H4), 7.40 (d, 1H, H5, *J* = 8.5 Hz), 7.30 (s, 2H, H17, (-NH_2_), 6.89 (d, 1H, H6, *J* = 8.5 Hz); IR (ATR): 3561, 3440, 3298, 3083, 3044, 1680, 1610, 1577, 1499, 1374, 1337, 1292, 1119, 1085, 1054, 1026, 808, 782, 772, 701, 502, 471 cm^−1^; UV-Vis (MeOH): λ_max1_ = 272 nm, λ_max2_ = 391 nm; fluorescence (MeOH): λ_Em(Ex380)_ = 501 nm

### 3.5. AChE and BuChE Inhibition Activity

The acetylcholinesterase (AChE) and butyrylcholinesterase (BuChE) inhibition assays were performed via Ellman’s method [50] with slight modifications as described previously [29]. Briefly, 1 mg of each sample (**1**-**12**) was dissolved in 1 mL of DMSO and diluted with water to obtain a 0.1 μM solution. To the following wells of a 96-well transparent plate, an increasing concentration of tested samples (**1**-**12**) were added followed by AChE (0.05 U) or BuChE (0.023 U). Next, 20 μL of pH 8 buffer solution and 20 μL of Ellman’s reagent (10 mM) were added to all wells. The plate was then incubated for 10 min at 37 °C and prior to starting the absorbance measurements at 412 nm, 5 μL of the corresponding substrate (75 mM) was added to each sample. Changes in absorbance were recorded for 30 min and tacrine was used as the standard. The final volume of each sample was 200 μL and the concentration of DMSO did not exceed 0.005% per well and was considered negligible. The results were presented as the IC_50_ values, which correspond to the concentration of the potential inhibitor that inhibits the enzyme activity by 50%. All experiments were performed in three separate replicates, and the results obtained were averaged. All compounds were tested at concentrations not compromised by their sparing solubility.

### 3.6. Antibacterial Activity

The antibacterial activity of all compounds was evaluated by the minimum inhibitory concentration (MIC, mg/mL) evaluation with the use of the microdilution method [51,52]. Briefly, each bacterial strain was incubated in 37 °C in a New Brunswick Innova 40 Shaker (Eppendorf AG, Hamburg, Germany) until turbidity of 0.5 McFarland’s standard (10^8^ CFU/mL, colony-forming units per mL) was obtained and bacterial cultures were diluted to a final density of 10^5^ CFU/mL. In all experiments, appropriate controls were applied: a positive control of culture growth (MHB medium with no extract added), a negative control (MHB medium with tested compounds and no bacterial cultures added), and solvent control (serial dilutions of DMSO). The experiments were carried out with saturated solutions and the dilutions of all compounds in triplicate. MIC was defined as the lowest concentration of tested compounds, which completely inhibited the visible growth of each microorganism. In ambiguous cases, the results were compared with measurement of the optical density at 630 nm using a BIO-RAD Microplate Reader. Minimum bactericidal concentration (MBC) was determined by transferring 20 µL aliquots from the well obtained from the MIC experiment (MIC value) and two wells above the MIC value. Aliquots were seeded on MHA plates and incubated for 24 h at 37 °C. The number of visible colonies was counted manually and the concentration of sample that produces < 10 colonies was considered as the MBC value. Each experiment was carried out in triplicate.

### 3.7. Antifungal Activity

The effect of coumarin–thiadiazole hybrids on the growth of the selected fungal species was determined by measuring the optical density (OD_600_) in microcultures using a Bioscreen C system (Labsystem, Helsinki, Finland). Cultures were centrifuged and pellets were diluted in 0.9% NaCl to an optical density OD_600_ of 0.5. Aliquots of 250 μL of previously prepared dilutions of the test compounds in the appropriate culture medium (at concentrations 16, 32, 64, 128, 256 μg/mL) were applied into each well of a sterile 100-well Bioscreen plate. Three replicates were performed for each concentration. Subsequently, 50 μL of the bacterial suspension was added to each well. The control for fungal growth included wells containing a fungal cell suspension without tested compounds and the background control contained only growth media. Optical density (OD) cultures were measured at two-hour intervals for 48 h at 28 °C and at λ = 600 nm. To assess the effect of tested compounds on fungal growth, the data obtained were analyzed using the PYTHON script in accordance with Hoeflinger et al. [53]. It was used to calculate to parameters such as lag time, doubling time, delta OD, maximum specific growth rate, and other parameters. Final results were expressed as minimal inhibitory concentrations (MICs), defined as the lowest concentration of the antifungal agent causing a turbidity change compared to that of the control.

### 3.8. MTT Assay

The activity of coumarin–thiadiazole hybrids **1-12** was assessed using the MCF-7 cell line, which is a breast cancer-derived cell line. Testing was carried out using the methylthiazolyldiphenyl-tetrazolium bromide (MTT) assay following 96 h exposure of the cells. This method is based on the reduction of the tetrazolium salt MTT (3-(4, 5-dimethylthiazolyl-2)-2,5-diphenyltetrazolium bromide) into a crystalline blue formazan product by the cellular oxidoreductases of viable cells. The resultant formazan crystal formation is proportional to the number of viable cells. Cells were seeded at 4 × 10^5^ cells/mL in 96-well plates and incubated at 37 °C in 5% CO_2_ for 96 h. Cells were treated with a range of concentrations of the test compounds, in triplicate, from 0.2 to 200 µM or with a solvent control (0.5% DMSO) in complete medium. After 24 h incubation, the cells were assayed by the addition of one-tenth (20 µL) of the culture volume with MTT (5 mg/mL) in 0.1 M phosphate-buffered saline (PBS), pH 7.4, at 37 °C in a humid atmosphere with 5% CO_2_ for 4 h. The medium was then gently aspirated from test cultures and 100 µL of DMSO was added to each well. The plates were then shaken for 2 min and the absorbance was read at 550 nm in a Varioscan plate reader. The IC_50_ value was defined as the concentration of test compound required to reduce the absorbance of the MTT–formazan crystals by 50%, indicating 50% reduction in cellular activity.

## 4. Conclusions

In conclusion, a series of novel coumarin–thiadiazole hybrids was isolated and their structural characteristics were investigated in detail with the use of spectroscopic techniques. A number of the new compounds showed highly intensive emission in the visible region of the spectra. The particularly unusual absorption features of compound **1** together with its extraordinary emission properties are most likely due to its structural similarities to known coumarin derivatives used as fluorescence standards and point at the possibility for potential practical application of this compound as a fluorescence standard or fluorescence imaging agent. In this context the hydroxy-substituted compound **10** is equally interesting, though its absorption characteristics are more typical. More detailed studies on the excited state properties of **1** and **10** are currently in progress. It is also worth mentioning that the intensive fluorescence emission in the hybrids seems associated with the presence of the electron-donating substituent at the C7 carbon of the coumarin nucleus. It is highly likely that the appropriate substituent at the C7 carbon together with the relatively high rigidity of the coumarin–thiadiazole scaffolding is the key feature responsible for the high molar absorptivity value and intensive fluorescence emission.

The anti-cholinesterase and antimicrobial assays did not reveal significant activities for the novel hybrids. Clearly, the directly linked coumarin and thiadiazole nuclei do not offer the expected enhancement in the antineurodegenerative and antimicrobial potency. This issue might be addressed by introduction of a linker which would increase the distance between two pharmacophores. Moreover, such a linker would notably increase the flexibility of the hybrid, making the docking into the cholinesterase and other enzymes more effective. Therefore, the coumarin–thiadiazole hybrids incorporating an additional linker will be designed during the course of our future studies.

In terms of the antimicrobial activity, the coumarin–thiadiazole core in **1-12** does not offer a new therapeutic target, though the presence of an easily modifiable amino group enables the possibility for further modifications and especially those which would result in an improved metal-binding ability and the formation of a new series of metal-based agents.

## Figures and Tables

**Table 1:** Electronic absorption, molar absorptivity, and fluorescence emission maxima values in coumarin–thiadiazole hybrids **1-12**.

Compound	λ_max1_ (nm)	λ_max2_ (nm)	ε (×10^3^ M^−1^ cm^−1^)	λ_Em_ (nm)
**1**	285	445	38.12	509
**2**	307	367	9.18	513
**3**	306	368	10.57	511
**4**	284	370	8.71	524
**5**	284	374	13.46	530
**6**	272	371	1.38	-
**7**	286	375	7.12	-
**8**	282	370	10.34	518
**9**	290	378	2.57	514
**10**	254	385	14.31	504
**11**	258	364	9.83	513
**12**	272	391	7.80	501

**Table 2:** AChE and BuChE inhibition activity of coumarin–thiadiazole hybrids **1-12**.

Compound	AChE	BuChE
	IC_50_ (μM) ± SD *	IC_50_ (μM) ± SD *
**1**	0.184 ± 0.011	0.191 ± 0.024
**2**	0.213 ± 0.009	0.198 ± 0.039
**3**	0.152 ± 0.003	0.295 ± 0.024
**4**	0.211 ± 0.010	0.205 ± 0.014
**5**	0.183 ± 0.018	0.261 ± 0.015
**6**	0.198 ± 0.016	0.166 ± 0.022
**7**	0.159 ± 0.015	0.494 ± 0.024
**8**	0.199 ± 0.003	0.392 ± 0.024
**9**	0.196 ± 0.022	0.235 ± 0.034
**10**	0.200 ± 0.012	0.380 ± 0.010
**11**	0.165 ± 0.003	0.202 ± 0.030
**12**	0.187 ± 0.015	0.264 ± 0.017
**Tacrine**	0.059 ± 0.002	0.079 ± 0.008

* Data are expressed as mean ± standard deviation (*n* = 3).

**Table 3:** Minimal inhibitory concentration (MIC) and minimal bactericidal concentration (MBC) values determined for coumarin–thiadiazole hybrids **1-12**.

Compound	MIC/MBC ^a^ (mg/mL)				
	Gram-Negative			Gram-Positive	
	*E. coli*	*P. aeruginosa*	*S. aureus*	*S. epidermidis* ATCC 12228	*S. epidermidis* ATCC 35984
**1**	1.79	1.79	0.22	0.89/7.14	1.79
**2**	NI	NI	0.49	0.49/0.49	NI
**3**	NI	NI	0.51	0.51/0.51	NI
**4**	NI	NI	3.57	0.45/1.79	0.45
**5**	0.89	NI	0.89	0.11	0.45
**6**	0.06	0.25	0.03	0.03	0.06
**7**	0.89	NI	0.89	3.57	0.89
**8**	NI	NI	NI	0.06/0.12	NI
**9**	NI	NI	1.22	0.61/1.22	NI
**10**	NI	NI	0.51	0.25/1.02	NI
**11**	NI	NI	0.12	0.1	0.06
**12**	NI	NI	NI	NI	NI
chloramphenicol	3.9 × 10^−3^	0.25	7.8 × 10^−3^	7.8 × 10^−3^	1.56 × 10^−2^
gentamicin	1.9 × 10^−3^	1.9	0.97 × 10^−3^	0.24 × 10^−3^	3.12 × 10^−2^
kanamycin	7.8 × 10^−3^	NI	3.9 × 10^−3^	1.9 × 10^−3^	NI

^a^—identified only in the concentration range tested. NI—no visible inhibition of bacterial growth in the concentration range tested.

**Table 4:** Assessment of antifungal activities of coumarin–thiadiazole hybrids **1-12** expressed as MIC values against *C. parapsilosis*, *S. cerevisiae*, *A. fumigatus*, *F. oxysporum*.

Compound	Strain/MIC (μg/mL)				
	*C. parapsilosis*	*S. cerevisiae*	*A. flavus*	*A. fumigatus*	*F. oxysporum*
**1**	>256	>256	>256	>256	128
**2**	>256	>256	256	>256	256
**3**	>256	>256	>256	128	>256
**4**	>256	>256	>256	64	>256
**5**	>256	>256	>256	>256	>256
**6**	>256	>256	>256	64	>256
**7**	>256	>256	>256	64	64
**8**	>256	128	256	>256	>256
**9**	>256	>256	>256	>256	128
**10**	>256	>256	256	64	>256
**11**	>256	>256	>256	>256	>256
**12**	>256	>256	>256	>256	>256
**Amphotericin B**	2	2	2	1	2

## Data Availability

The data presented in this study are available on request from the corresponding author.

## Supplementary Materials

The following are available online at https://www.mdpi.com/article/10.3390/ijms23116314/s1.

## References

[1] Medina F.G., Marrero J.G., Macías-Alonso M., González M.C., Córdova-Guerrero I., Teissier García A.G., Osegueda-Robles S. (2015). Coumarin heterocyclic derivatives: Chemical synthesis and biological activity. Nat. Prod. Rep..

[2] Annunziata F., Pinna C., Dallavalle S., Tamborini L., Pinto A. (2020). An Overview of Coumarin as a Versatile and Readily Accessible Scaffold with Broad-Ranging Biological Activities. Int. J. Mol. Sci..

[3] Hu Y., Li C.-Y., Wang X.-M., Yang Y.-H., Zhu H.-L. (2014). 1,3,4-Thiadiazole: Synthesis, Reactions, and Applications in Medicinal, Agricultural, and Materials Chemistry. Chem. Rev..

[4] Serban G., Stanasel O., Serban E., Bota S. (2018). 2-Amino-1,3,4-thiadiazole as a potential scaffold for promising antimicrobial agents. Drug Des. Dev. Ther..

[5] Cao D., Liu Z., Verwilst P., Koo S., Jangjili P., Kim J.S., Lin W. (2019). Coumarin-Based Small-Molecule Fluorescent Chemosensors. Chem. Rev..

[6] Ioannis F., Dimitra H.-L. (2020). Hybrids of Coumarin Derivatives as Potent and Multifunctional Bioactive Agents: A Review. Med. Chem..

[7] Dawood K.M., Farghaly T.A. (2017). Thiadiazole inhibitors: A patent review. Expert Opin. Ther. Pat..

[8] Maříková J., Mamun A.A., Shammari L.A., Korábečný J., Kučera T., Hulcová D., Kuneš J., Malaník M., Vašková M., Kohelová E. (2021). Structure Elucidation and Cholinesterase Inhibition Activity of Two New Minor Amaryllidaceae Alkaloids. Molecules.

[9] Kos J., Kozik V., Pindjakova D., Jankech T., Smolinski A., Stepankova S., Hosek J., Oravec M., Jampilek J., Bak A. (2021). Synthesis and Hybrid SAR Property Modeling of Novel Cholinesterase Inhibitors. Int. J. Mol. Sci..

[10] Carneiro A., Matos M.J., Uriarte E., Santana L. (2021). Trending Topics on Coumarin and Its Derivatives in 2020. Molecules.

[11] Şahin Ö., Özmen Özdemir Ü., Seferoğlu N., Adem Ş., Seferoğlu Z. (2021). Synthesis, characterization, molecular docking and in vitro screening of new metal complexes with coumarin Schiff base as anticholine esterase and antipancreatic cholesterol esterase agents. J. Biomol. Struct. Dyn..

[12] Masroor A., Chandel T.I., Malik S., Mateen Q.N., Uversky V.N., Khan R.H. (2021). Evaluation of ThT augmentation and RLS inner filter effect caused by highly fluorescent coumarin derivative and establishing it as true inhibitor of amyloid fibrillation. Arch. Biochem. Biophys..

[13] De Souza L.G., Rennó M.N., Figueroa-Villar J.D. (2016). Coumarins as cholinesterase inhibitors: A review. Chem.-Biol. Interact..

[14] Ujan R., Saeed A., Channar P.A., Larik F.A., Abbas Q., Alajmi M.F., El-Seedi H.R., Rind M.A., Hassan M., Raza H. (2019). Drug-1,3,4-Thiadiazole Conjugates as Novel Mixed-Type Inhibitors of Acetylcholinesterase: Synthesis, Molecular Docking, Pharmacokinetics, and ADMET Evaluation. Molecules.

[15] Skrzypek A., Matysiak J., Karpińska M., Czarnecka K., Kręcisz P., Stary D., Kukułowicz J., Paw B., Bajda M., Szymański P. (2021). Biological evaluation and molecular docking of novel 1,3,4-thiadiazole-resorcinol conjugates as multifunctional cholinesterases inhibitors. Bioorg. Chem..

[16] Balewski Ł., Szulta S., Jalińska A., Kornicka A. (2021). A Mini-Review: Recent Advances in Coumarin-Metal Complexes with Biological Properties. Front. Chem..

[17] Frija L.M.T., Pombeiro A.J.L., Kopylovich M.N. (2016). Coordination chemistry of thiazoles, isothiazoles and thiadiazoles. Coord. Chem. Rev..

[18] Wang L., Yin Y.-L., Liu X.-Z., Shen P., Zheng Y.-G., Lan X.-R., Lu C.-B., Wang J.-Z. (2020). Current understanding of metal ions in the pathogenesis of Alzheimer’s disease. Transl. Neurodegener..

[19] Huat T.J., Camats-Perna J., Newcombe E.A., Valmas N., Kitazawa M., Medeiros R. (2019). Metal Toxicity Links to Alzheimer’s Disease and Neuroinflammation. J. Mol. Biol..

[20] Liu Y., Nguyen M., Robert A., Meunier B. (2019). Metal Ions in Alzheimer’s Disease: A Key Role or Not?. Acc. Chem. Res..

[21] Bakulski K.M., Seo Y.A., Hickman R.C., Brandt D., Vadari H.S., Hu H., Park S.K. (2020). Heavy Metals Exposure and Alzheimer’s Disease and Related Dementias. J. Alzheimer’s Dis. JAD.

[22] MacLean L., Karcz D., Jenkins H., McClean S., Devereux M., Howe O., Pereira M.D., May N.V., Enyedy É.A., Creaven B.S. (2019). Copper (II) complexes of coumarin-derived Schiff base ligands: Pro- or antioxidant activity in MCF-7 cells?. J. Inorg. Biochem..

[23] Karcz D., Matwijczuk A., Boroń B., Creaven B., Fiedor L., Niewiadomy A., Gagoś M. (2017). Isolation and spectroscopic characterization of Zn(II), Cu(II), and Pd(II) complexes of 1,3,4-thiadiazole-derived ligand. J. Mol. Struct..

[24] Karcz D., Starzak K., Matwijczuk A., Ciszkowicz E., Lecka-Szlachta K., Matwijczuk A., Ciupak A., Gładyszewska B., Niewiadomy A. (2018). Synthesis, spectroscopy and biological activity of novel Cu (II) and Cn (II) complexes with 1,3,4-thiadiazole derivatives. Przem. Chem..

[25] David M., Budziak-Wieczorek I., Karcz D., Florescu M., Matwijczuk A. (2021). Insight into dual fluorescence effects induced by molecular aggregation occurring in membrane model systems containing 1,3,4-thiadiazole derivatives. Eur. Biophys. J..

[26] Czernel G., Budziak I., Oniszczuk A., Karcz D., Pustuła K., Górecki A., Matwijczuk A., Gładyszewska B., Gagoś M., Niewiadomy A. (2020). ESIPT-Related Origin of Dual Fluorescence in the Selected Model 1,3,4-Thiadiazole Derivatives. Molecules.

[27] Starzak K., Matwijczuk A., Creaven B., Matwijczuk A., Wybraniec S., Karcz D. (2019). Fluorescence quenching-based mechanism for determination of hypochlorite by coumarin-derived sensors. Int. J. Mol. Sci..

[28] Karcz D., Matwijczuk A., Kamiński D., Creaven B., Ciszkowicz E., Lecka-Szlachta K., Starzak K. (2020). Structural features of 1,3,4-thiadiazole-derived ligands and their Zn (II) and Cu (II) complexes which demonstrate synergistic antibacterial effects with kanamycin. Int. J. Mol. Sci..

[29] Karcz D., Starzak K., Ciszkowicz E., Lecka-Szlachta K., Kamiński D., Creaven B., Jenkins H., Radomski P., Miłoś A., Ślusarczyk L. (2021). Novel Coumarin-Thiadiazole Hybrids and Their Cu (II) and Zn (II) Complexes as Potential Antimicrobial Agents and Acetylcholinesterase Inhibitors. Int. J. Mol. Sci..

[30] Yusufzai S.K., Khan M.S., Sulaiman O., Osman H., Lamjin D.N. (2018). Molecular docking studies of coumarin hybrids as potential acetylcholinesterase, butyrylcholinesterase, monoamine oxidase A/B and β-amyloid inhibitors for Alzheimer’s disease. Chem. Cent. J..

[31] Hassan M.Z., Osman H., Ali M.A., Ahsan M.J. (2016). Therapeutic potential of coumarins as antiviral agents. Eur. J. Med. Chem..

[32] Calcio Gaudino E., Tagliapietra S., Martina K., Palmisano G., Cravotto G. (2016). Recent advances and perspectives in the synthesis of bioactive coumarins. RSC Adv..

[33] Chudzik B., Bonio K., Dabrowski W., Pietrzak D., Niewiadomy A., Olender A., Malodobry K., Gagoś M. (2019). Synergistic antifungal interactions of amphotericin B with 4-(5-methyl-1,3,4-thiadiazole-2-yl) benzene-1,3-diol. Sci. Rep..

[34] Mahmoud M.R., El-Shahawi M.M., Abu El-Azm F.S., Abdeen M. (2017). Synthesis and Antimicrobial Activity of Polyfunctionally Substituted Heterocyclic Compounds Derived from 5-Cinnamoylamino-2-Cyanomethyl-1,3,4-Thiadiazole. J. Heterocycl. Chem..

[35] Bhalla M., Hitkari A., Gujrati V.R., Bhalla T.N., Shanker K. (1994). Benzopyran-2-one derivatives: Antiinflammatory, analgesic and antiproteolytic agents. Eur. J. Med. Chem..

[36] Gomha S., Abdel-Aziz H. (2015). Synthesis and Antitumor Activity of 1,3,4-Thiadiazole Derivatives Bearing Coumarine Ring. Heterocycles.

[37] Creaven B.S., Egan D.A., Kavanagh K., McCann M., Noble A., Thati B., Walsh M. (2006). Synthesis, characterization and antimicrobial activity of a series of substituted coumarin-3-carboxylatosilver (I) complexes. Inorg. Chim. Acta.

[38] Song A., Wang X., Lam K.S. (2003). A convenient synthesis of coumarin-3-carboxylic acids via Knoevenagel condensation of Meldrum’s acid with ortho-hydroxyaryl aldehydes or ketones. Tetrahedron Lett..

[39] Maggi R., Bigi F., Carloni S., Mazzacani A., Sartori G. (2001). Uncatalysed reactions in water: Part 2. Preparation of 3-carboxycoumarins. Green Chem..

[40] Dolomanov O.V., Bourhis L.J., Gildea R.J., Howard J.A.K., Puschmann H. (2009). OLEX2: A complete structure solution, refinement and analysis program. J. Appl. Crystallogr..

[41] Sheldrick G. (2015). Crystal structure refinement with SHELXL. Acta Crystallogr. Sect. C.

[42] Sheldrick G.M. (2015). SHELXT—Integrated space-group and crystal-structure determination. Acta Crystallogr. Sect. A Found. Adv..

[43] Creaven B.S., Devereux M., Georgieva I., Karcz D., McCann M., Trendafilova N., Walsh M. (2011). Molecular structure and spectroscopic studies on novel complexes of coumarin-3-carboxylic acid with Ni (II), Co (II), Zn (II) and Mn (II) ions based on density functional theory. Spectrochim. Acta Part A Mol. Biomol. Spectrosc..

[44] Silverstein R.M., Webster F.X., Kiemle D. (2005). Spectrometric Identification of Organic Compounds.

[45] Georgieva I., Trendafilova N., Kiefer W., Rastogi V.K., Kostova I. (2007). Vibrational and theoretical study of coumarin-3-carboxylic acid binding mode in Ce (III) and Nd (III) complexes. Vib. Spectrosc..

[46] Kristoffersen A.S., Erga S.R., Hamre B., Frette Ø. (2014). Testing Fluorescence Lifetime Standards using Two-Photon Excitation and Time-Domain Instrumentation: Rhodamine B, Coumarin 6 and Lucifer Yellow. J. Fluoresc..

[47] Papakonstantinou I., Tummeltshammer C. (2015). Fundamental limits of concentration in luminescent solar concentrators revised: The effect of reabsorption and nonunity quantum yield. Optica.

[48] Aggarwal N., Jain S., Chopra N. (2022). Hybrids of thiazolidin-4-ones and 1,3,4-thiadiazole: Synthesis and biological screening of a potential new class of acetylcholinesterae inhibitors. Biointerface Res. Appl. Chem..

[49] Castro A., Martinez A. (2001). Peripheral and Dual Binding Site Acetylcholinesterase Inhibitors: Implications in treatment of Alzheimers Disease. Mini-Rev. Med. Chem..

[50] Ellman G.L., Courtney K.D., Andres V., Featherstone R.M. (1961). A new and rapid colorimetric determination of acetylcholinesterase activity. Biochem. Pharmacol..

[51] Bocian A., Ciszkowicz E., Hus K.K., Buczkowicz J., Lecka-Szlachta K., Pietrowska M., Petrilla V., Petrillova M., Legáth Ľ., Legáth J. (2020). Antimicrobial Activity of Protein Fraction from Naja ashei Venom against Staphylococcus epidermidis. Molecules.

[52] Pusz J., Ciszkowicz E., Lecka-Szlachta K., Wolowiec S., Woźnicka E. (2017). Synthesis and antibacterial activity of La (III), Ce (III), Pr (III), and Nd (III) complexes of chrysin-4’-sulfonate. Acta Pol. Pharm. Drug Res..

[53] Hoeflinger J.L., Hoeflinger D.E., Miller M.J. (2017). A dynamic regression analysis tool for quantitative assessment of bacterial growth written in Python. J. Microbiol. Methods.

