# Supplementary material for: Design, Spectroscopy, and Assessment of Cholinesterase Inhibition and Antimicrobial Activities of Novel Coumarin–Thiadiazole Hybrids
Source: Int J Mol Sci. 2022 Jun 5;23(11):6314. doi: 10.3390/ijms23116314 (PMC9180949; doi:10.3390/ijms23116314)
Supplement: Supplementary file 1 [file ijms-23-06314-s001.zip › Supplementary material DKarcz KS1.pdf]

Supplementary material

Design, spectroscopy, and assessment of cholinesterase inhibition and antimicrobial activities of novel coumarin-thiadiazole hybrids

Dariusz Karcz[1*], Karolina Starzak[1], Ewa Ciszkowicz[2], Katarzyna Lecka-Szlachta[2], Daniel Kamiński[3], Bernadette Creaven[4], Anna Miłoś[5] Hollie Jenkins[6], Lidia Ślusarczyk[7], and Arkadiusz Matwijczuk[7]

[1]Department of Chemical Technology and Environmental Analytics (C1), Faculty of Chemical Engineering and Technology, Cracow University of Technology, Cracow, Poland; [2]Department of Biotechnology and Bioinformatics, Faculty of Chemistry, Rzeszow University of Technology, Rzeszow, Poland; [3]Department of General and Coordination Chemistry and Crystallography, Institute of Chemical Sciences, Maria Curie-Sklodowska University in Lublin, Lublin, Poland; [4]School of Chemical and Pharmaceutical Sciences, Technological University Dublin, Dublin, Ireland; [5]Department of Biotechnology and Bioinformatics, Faculty of Chemistry, Doctoral School of Engineering and Technical Sciences at the Rzeszow University of Technology, Rzeszow, Poland, [6]Department of Applied Science, Technological University Dublin, Tallaght, Ireland; [7]Department of Biophysics, University of Life Sciences in Lublin, Lublin, Poland;

[*]Correspondence: dariusz.karcz@pk.edu.pl ; Tel.: +48(12)6282177

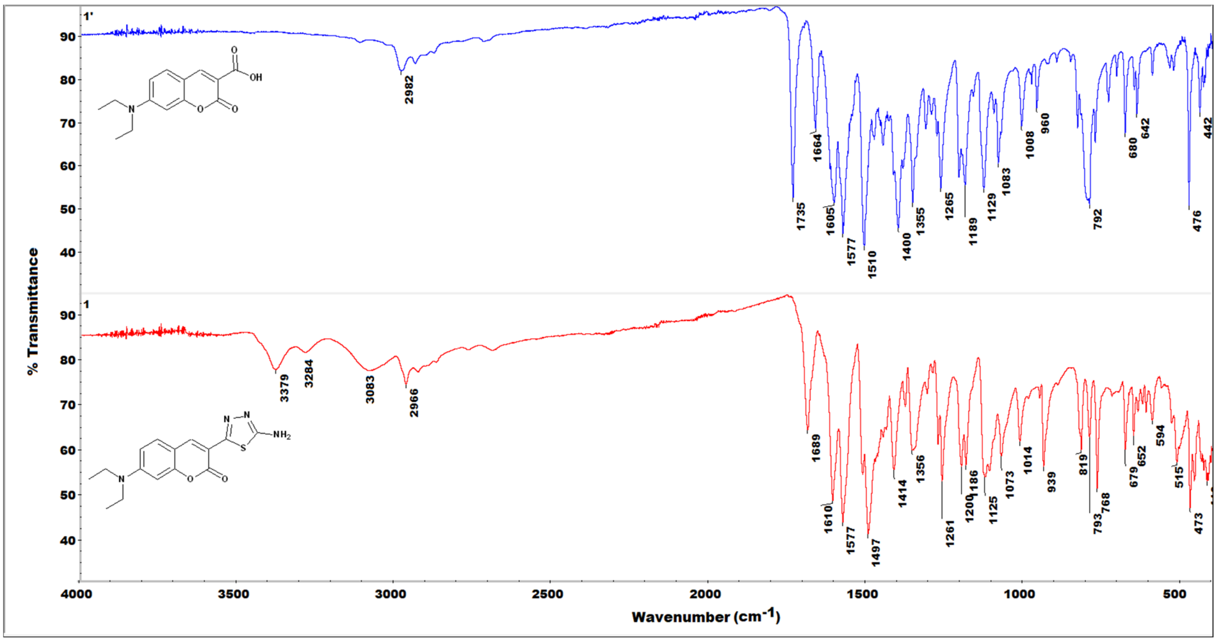

Figure S1. Comparison of the IR(ATR) spectra of coumarin-3-carboxylic acid **1’** (top) and the corresponding coumarin-thiadiazole hybrid **1** (bottom).

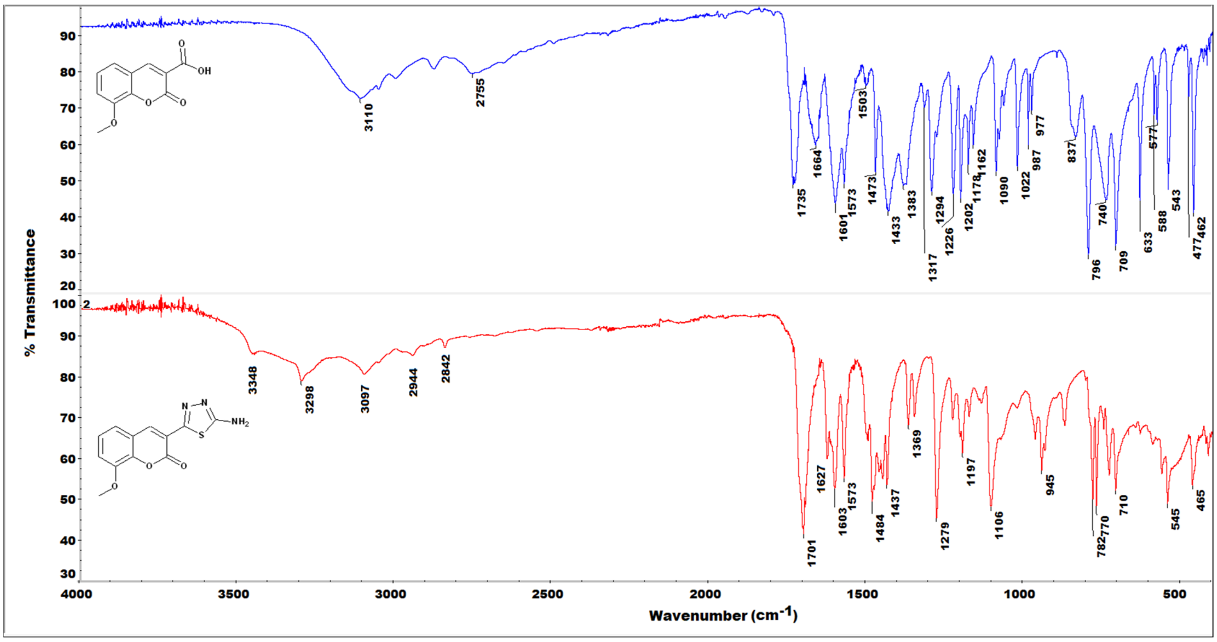

Figure S2. Comparison of the IR(ATR) spectra of coumarin-3-carboxylic acid **2’** (top) and the corresponding coumarin-thiadiazole hybrid **2** (bottom).

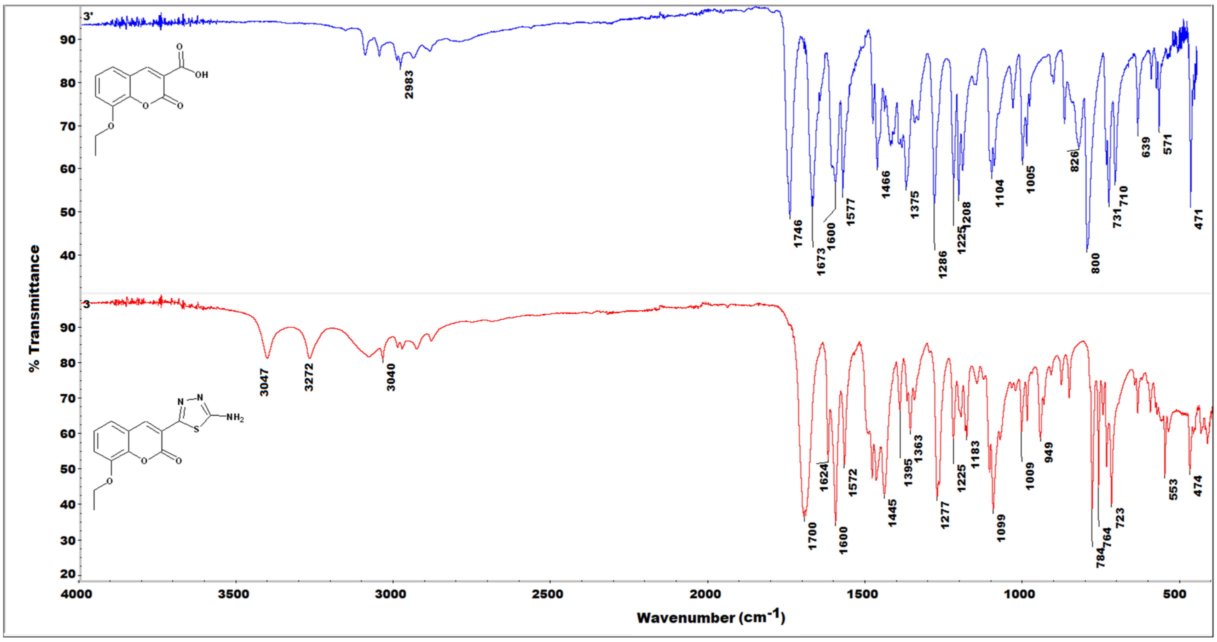

Figure S3. Comparison of the IR(ATR) spectra of coumarin-3-carboxylic acid **3’** (top) and the corresponding coumarin-thiadiazole hybrid **3** (bottom).

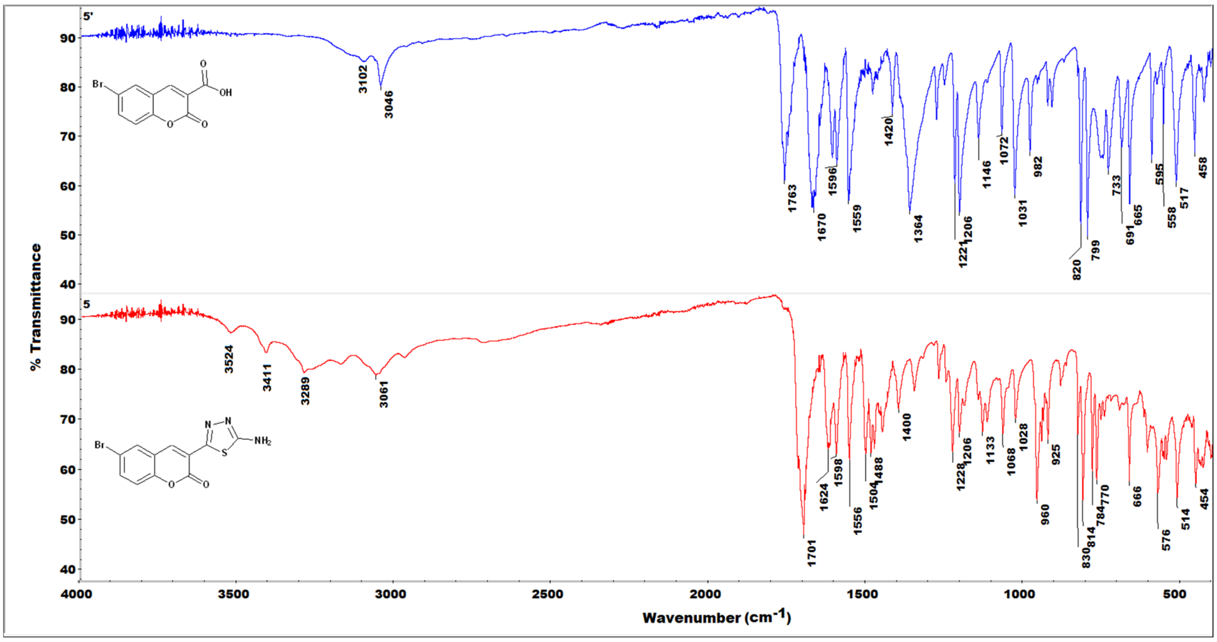

Figure S4. Comparison of the IR(ATR) spectra of coumarin-3-carboxylic acid **5’** (top) and the corresponding coumarin-thiadiazole hybrid **5** (bottom).

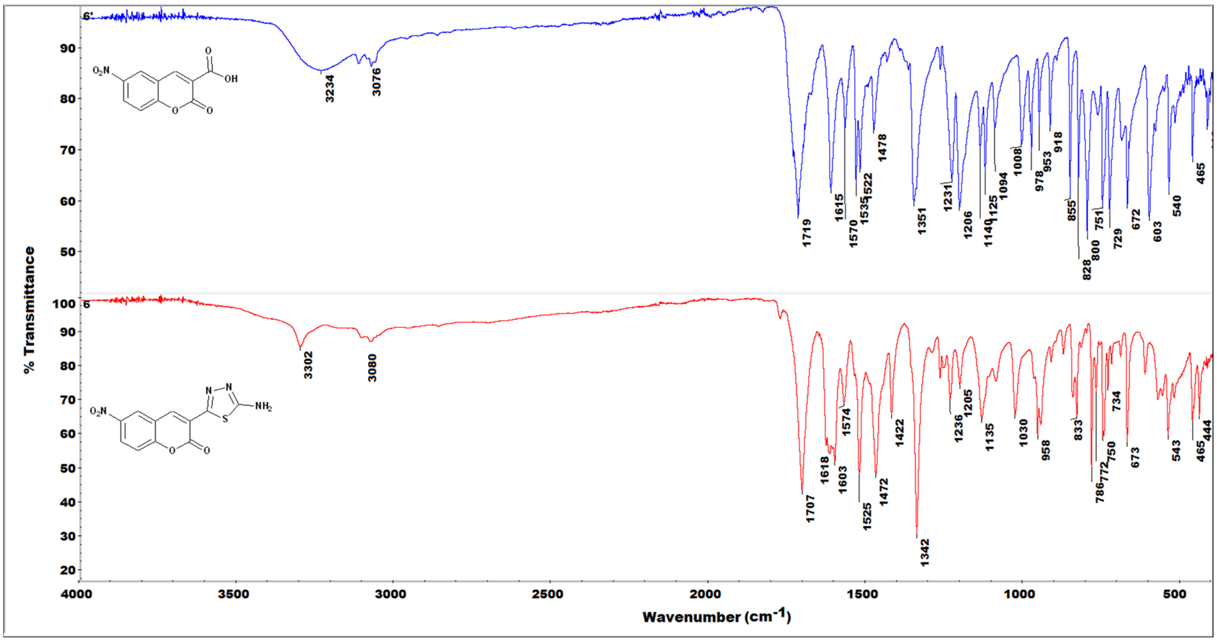

Figure S5. Comparison of the IR(ATR) spectra of coumarin-3-carboxylic acid **6’** (top) and the corresponding coumarin-thiadiazole hybrid **6** (bottom).

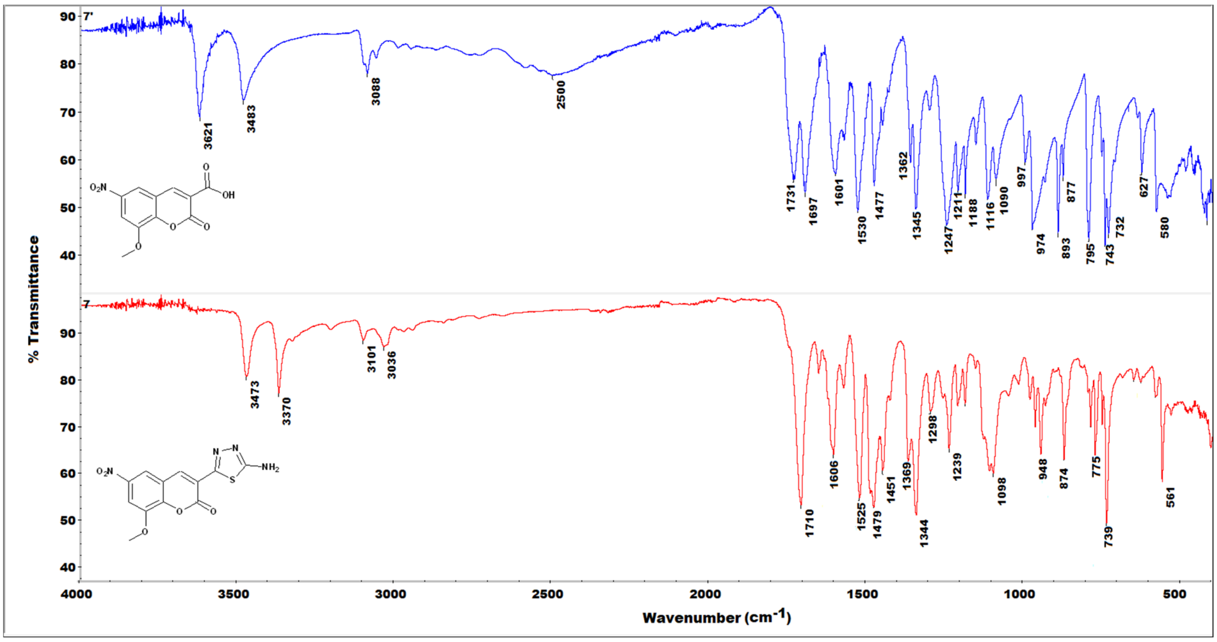

Figure S6. Comparison of the IR(ATR) spectra of coumarin-3-carboxylic acid **7’** (top) and the corresponding coumarin-thiadiazole hybrid **7** (bottom).

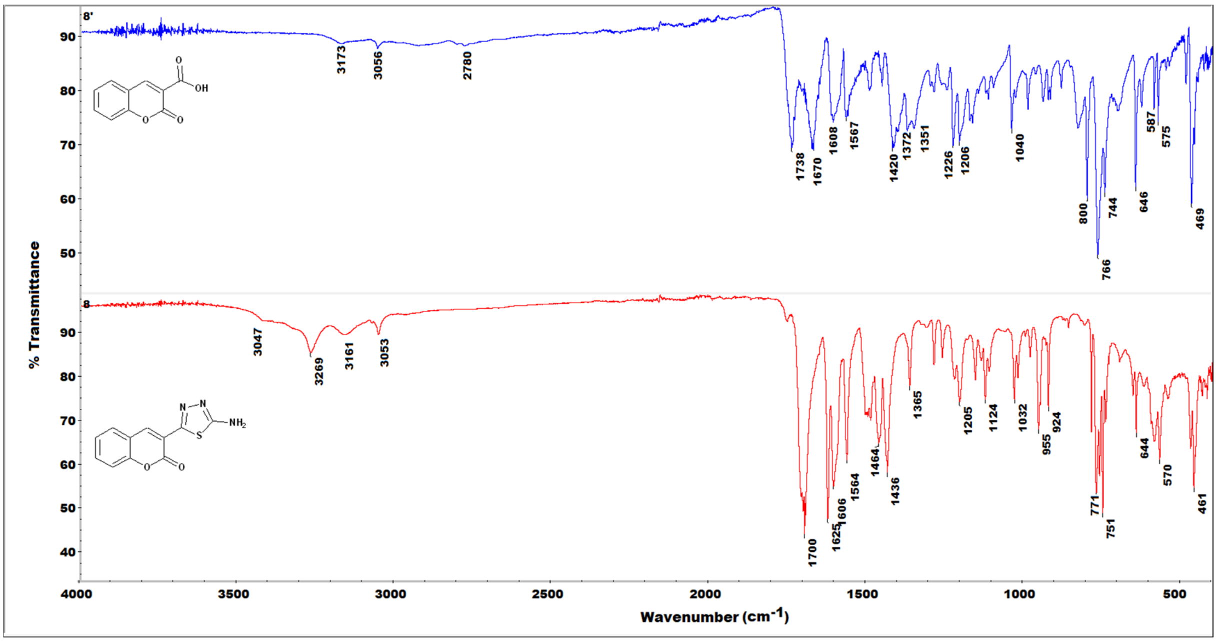

Figure S7. Comparison of the IR(ATR) spectra of coumarin-3-carboxylic acid **8’** (top) and the corresponding coumarin-thiadiazole hybrid **8** (bottom).

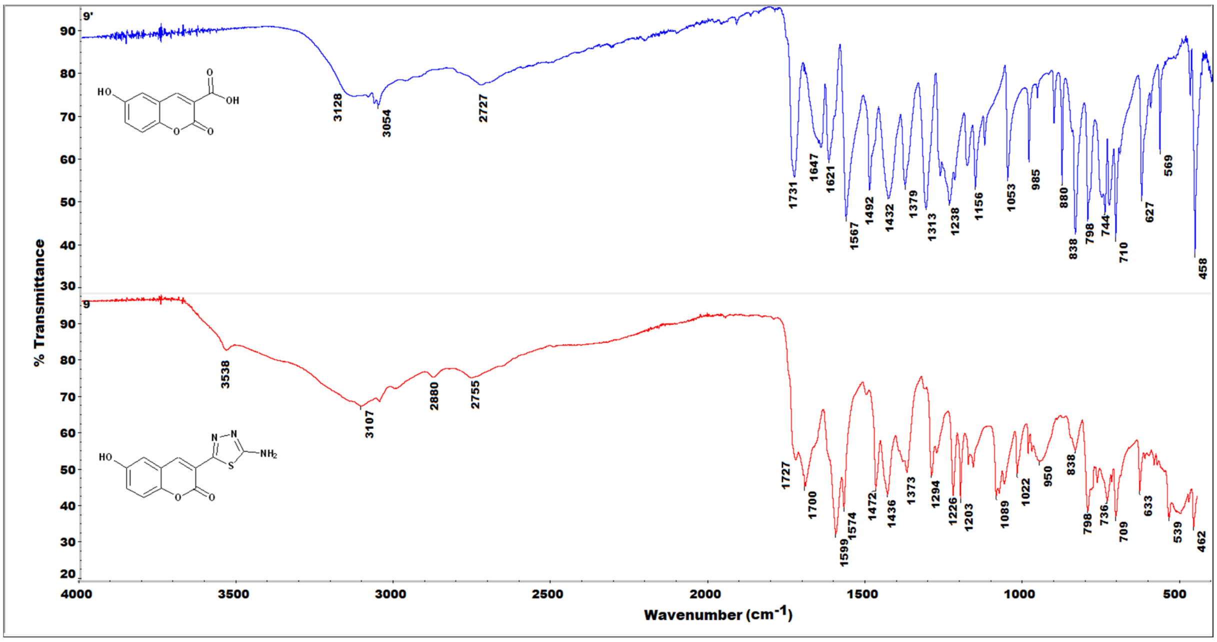

Figure S8. Comparison of the IR(ATR) spectra of coumarin-3-carboxylic acid **9’** (top) and the corresponding coumarin-thiadiazole hybrid **9** (bottom).

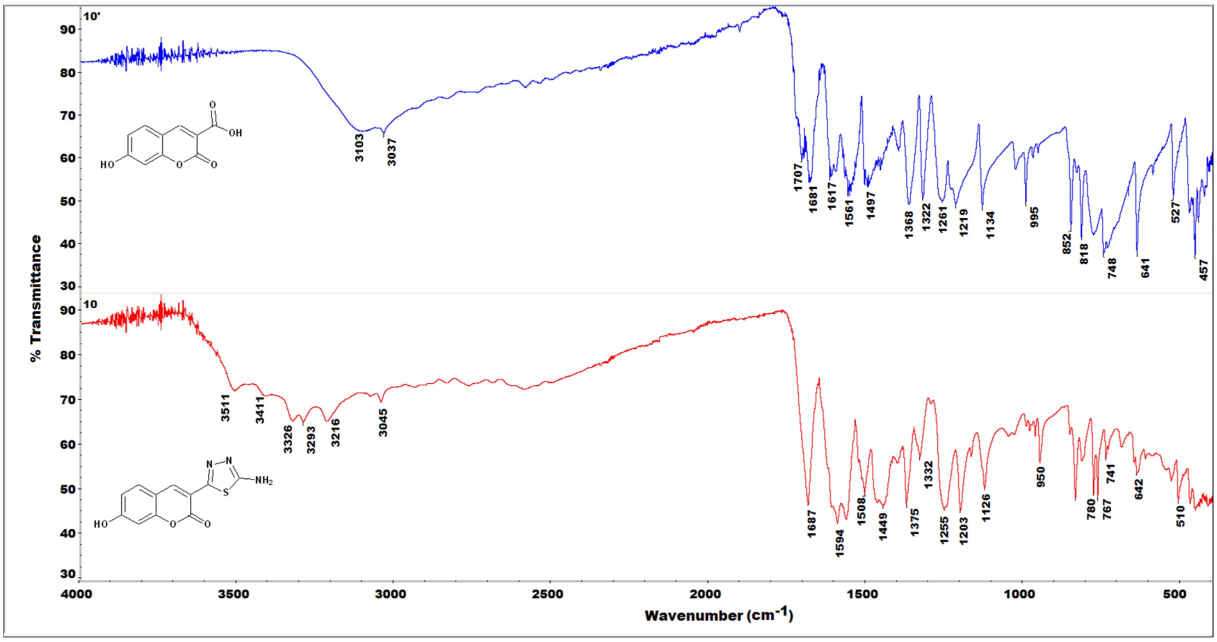

Figure S9. Comparison of the IR(ATR) spectra of coumarin-3-carboxylic acid **10’** (top) and the corresponding coumarin-thiadiazole hybrid **10** (bottom).

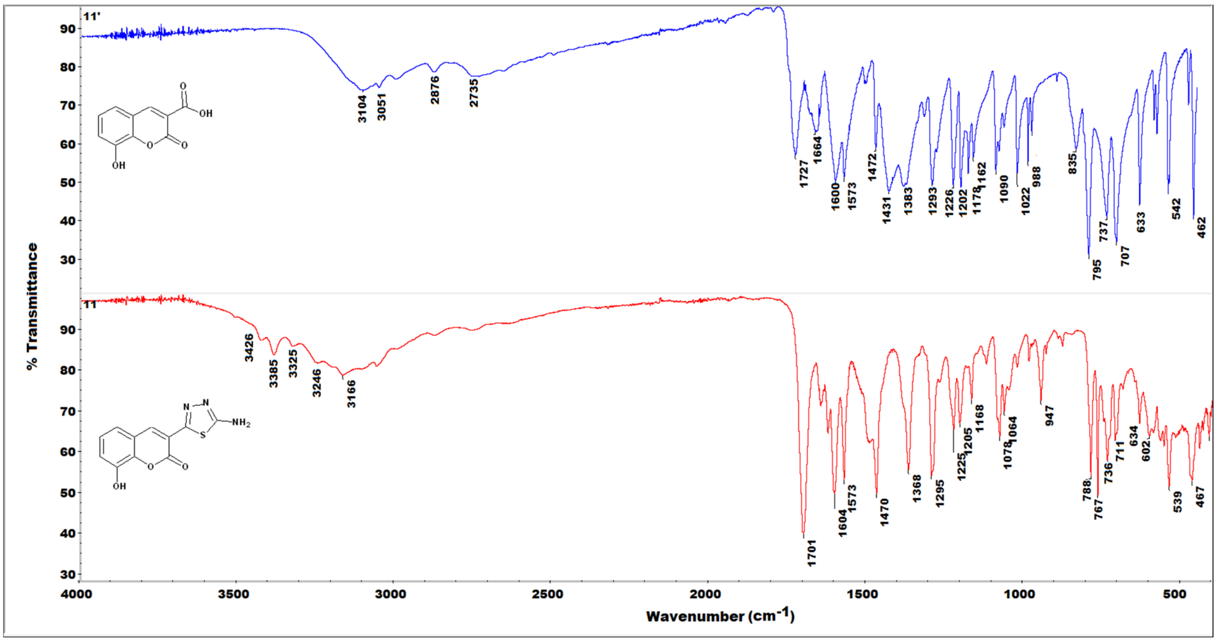

Figure S10. Comparison of the IR(ATR) spectra of coumarin-3-carboxylic acid **11’** (top) and the corresponding coumarin-thiadiazole hybrid **11** (bottom).

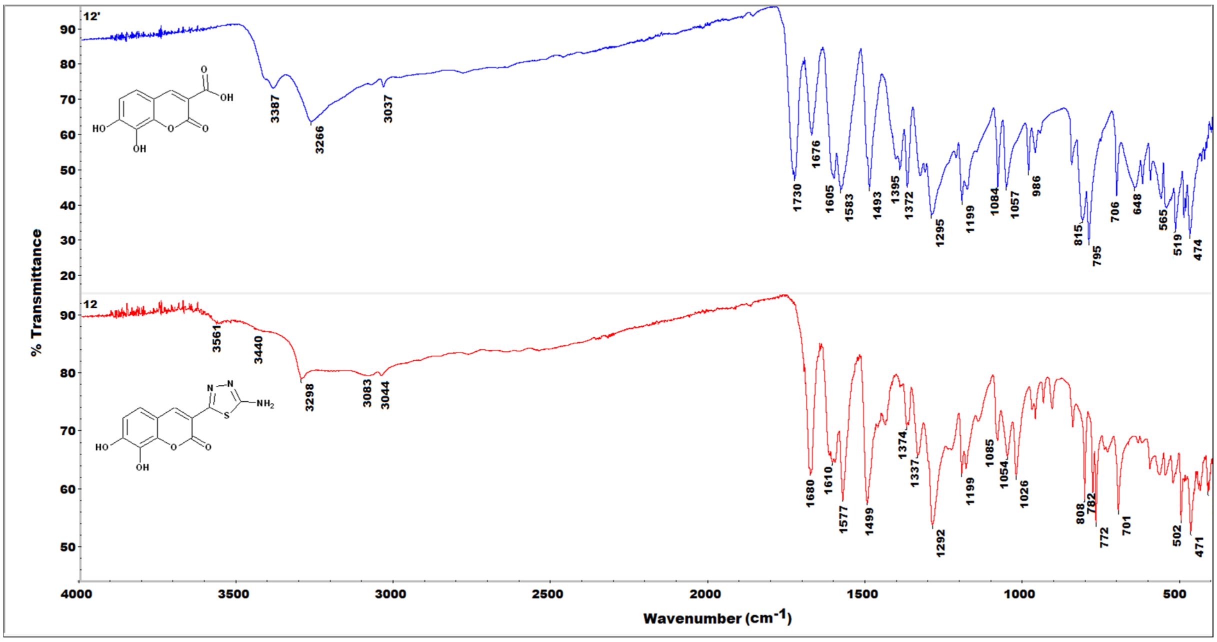

Figure S11. Comparison of the IR(ATR) spectra of coumarin-3-carboxylic acid **12’** (top) and the corresponding coumarin-thiadiazole hybrid **12** (bottom).

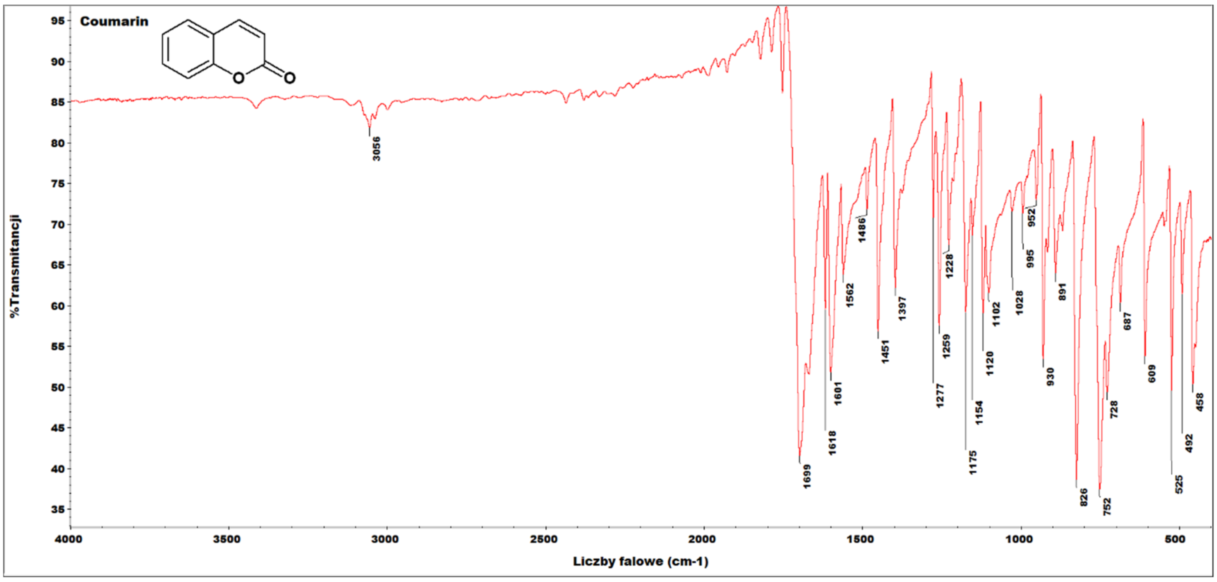

Figure S12. IR(ATR) spectrum of coumarin.

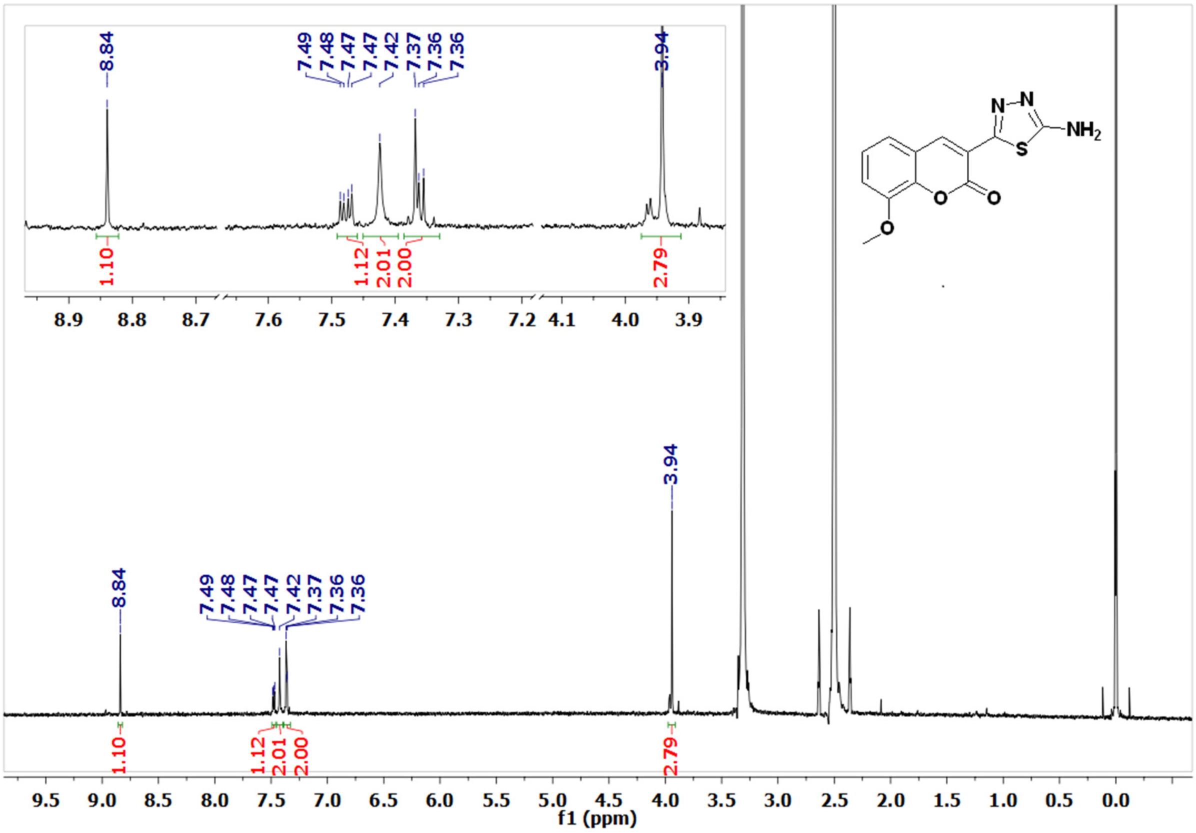

Figure S13. ^{1}H-NMR (DMSO$_{d6}$) spectrum of coumarin-thiadiazole hybrid **2.**

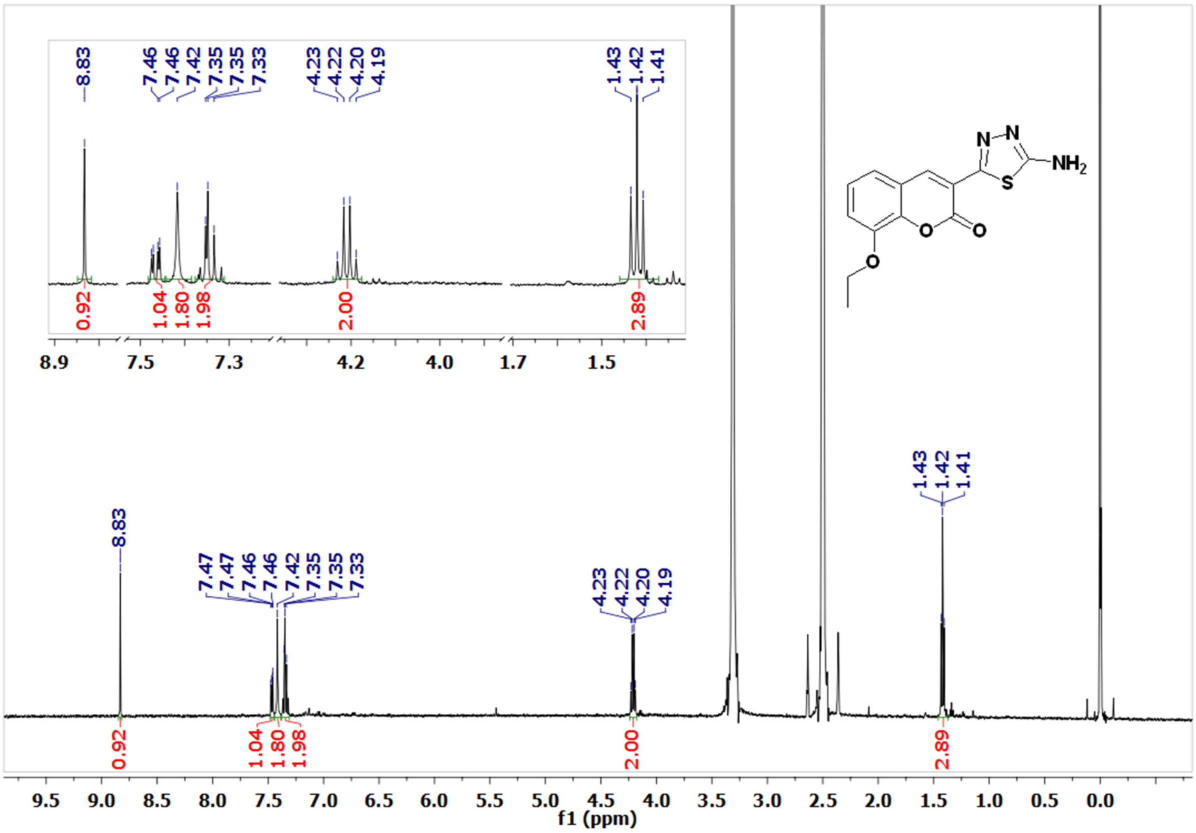

Figure S14. ^{1}H-NMR (DMSO$_{d6}$) spectrum of coumarin-thiadiazole hybrid **3.**

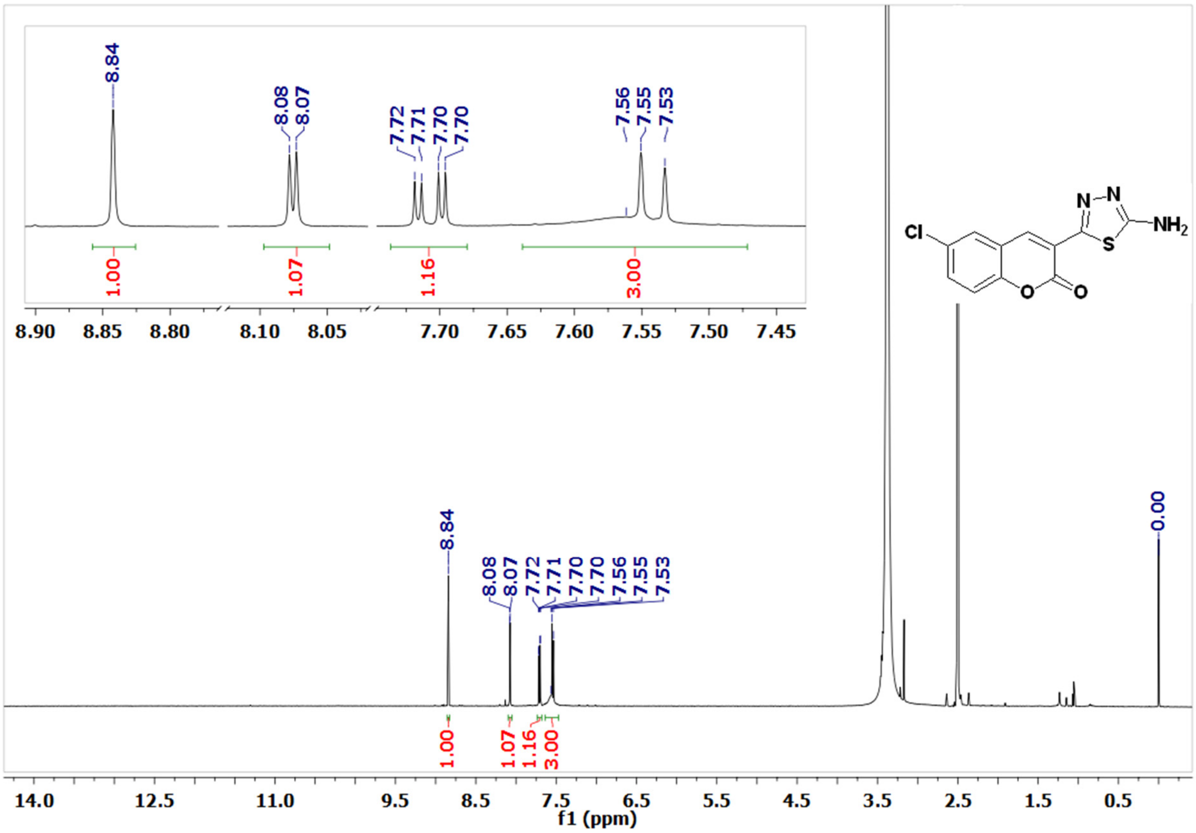

Figure S15. ^{1}H-NMR (DMSO$_{d6}$) spectrum of coumarin-thiadiazole hybrid **4.**

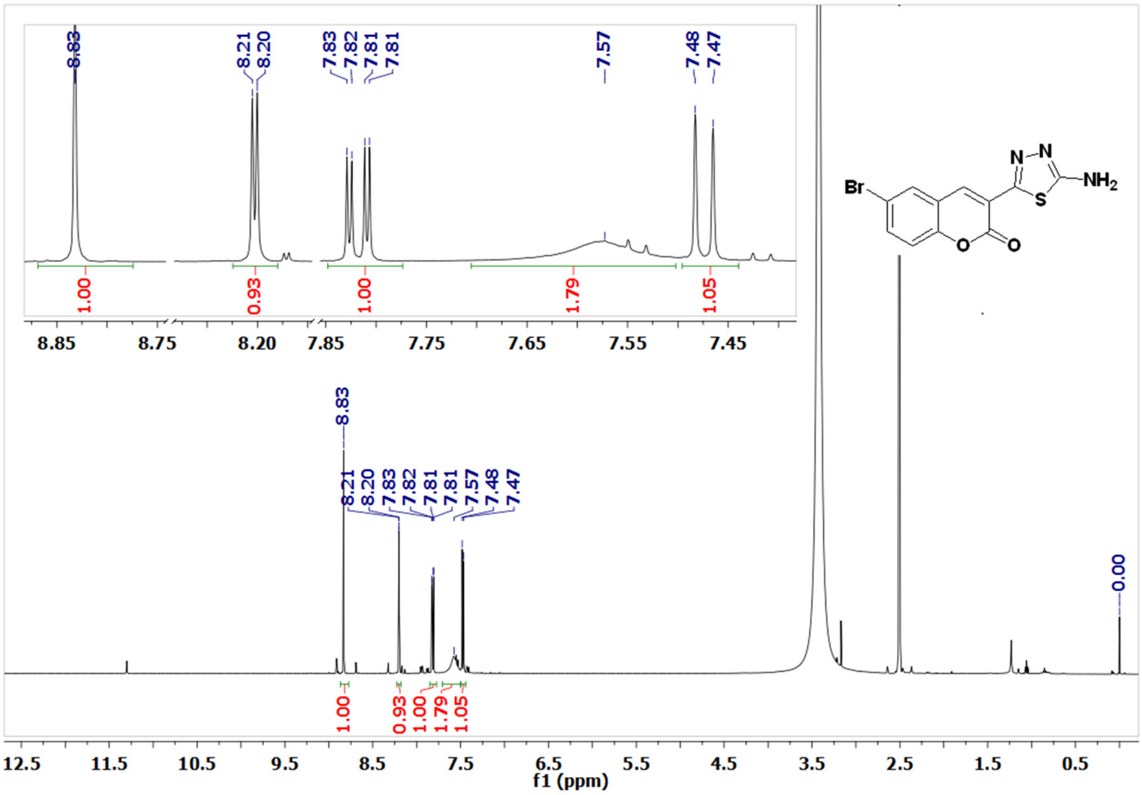

Figure S16. ^{1}H-NMR (DMSO$_{d6}$) spectrum of coumarin-thiadiazole hybrid **5.**

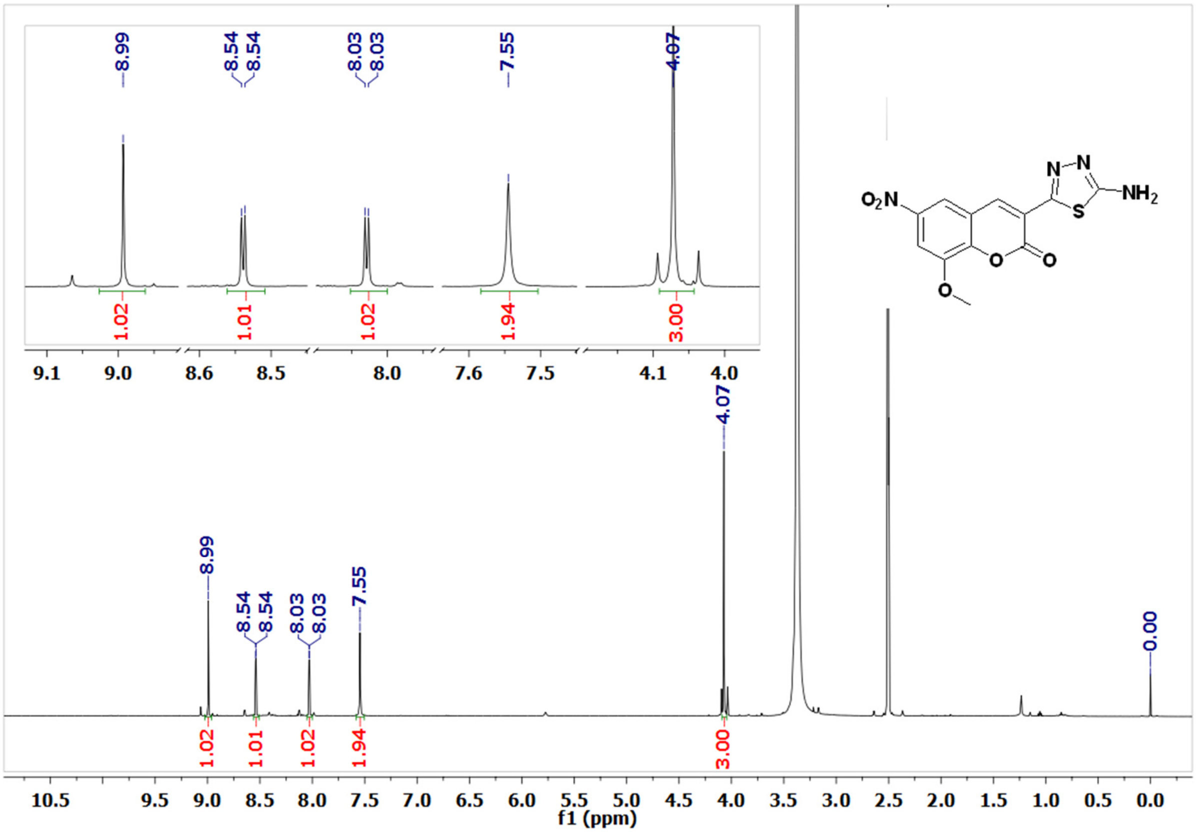

Figure S17. ^{1}H-NMR ($DMSO_{d6}$) spectrum of coumarin-thiadiazole hybrid **7.**

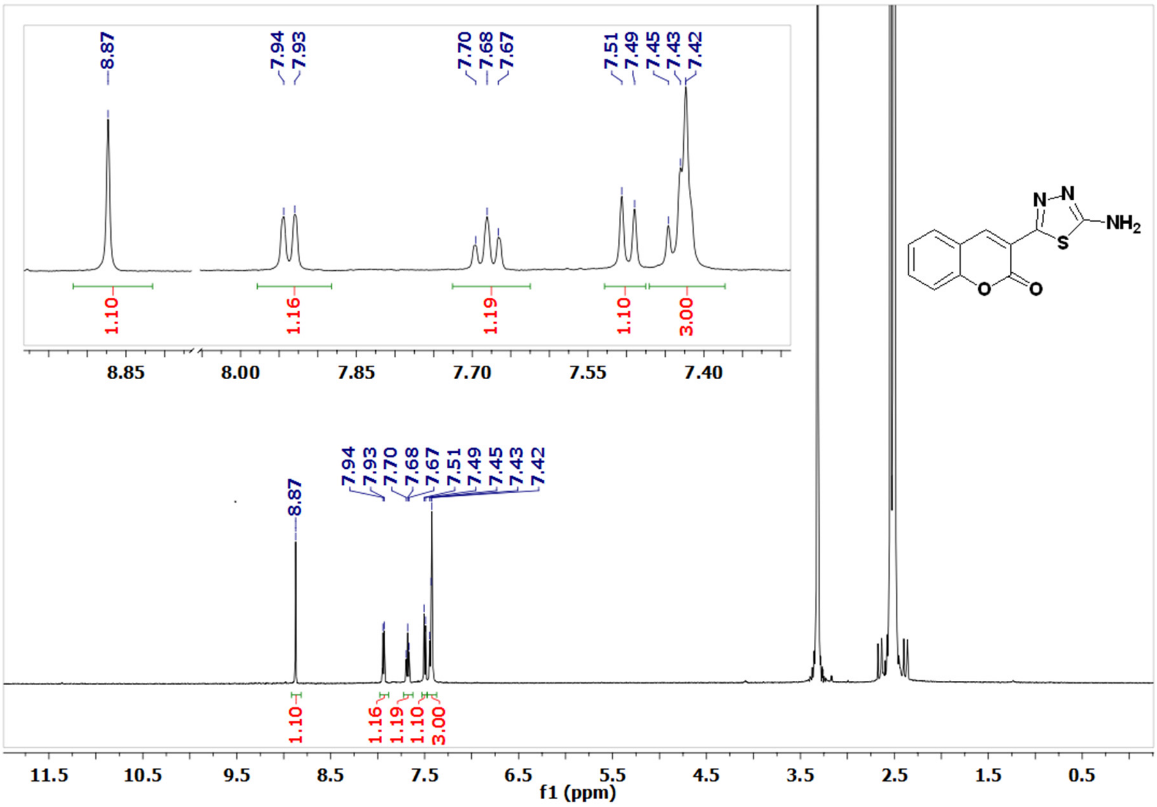

Figure S18. ^{1}H-NMR ($DMSO_{d6}$) spectrum of coumarin-thiadiazole hybrid **8.**

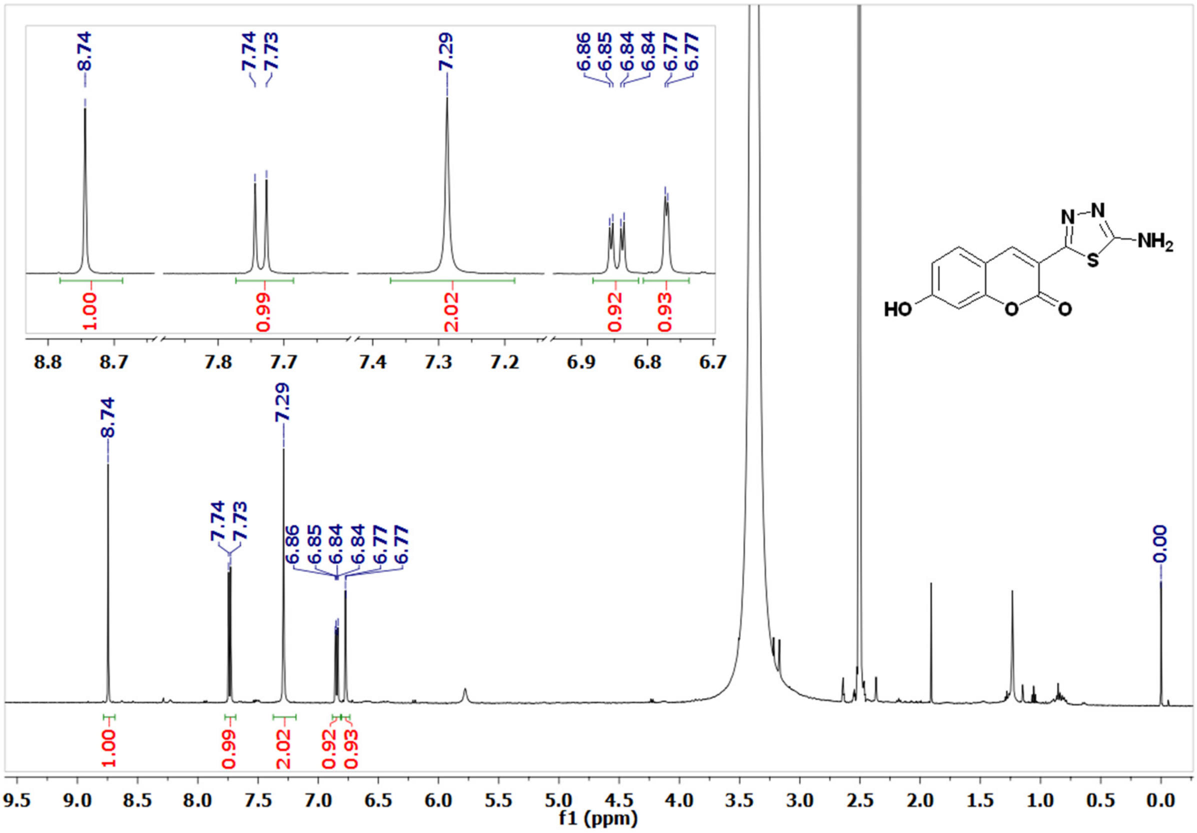

Figure S19. ^{1}H-NMR ($DMSO_{d6}$) spectrum of coumarin-thiadiazole hybrid **10.**

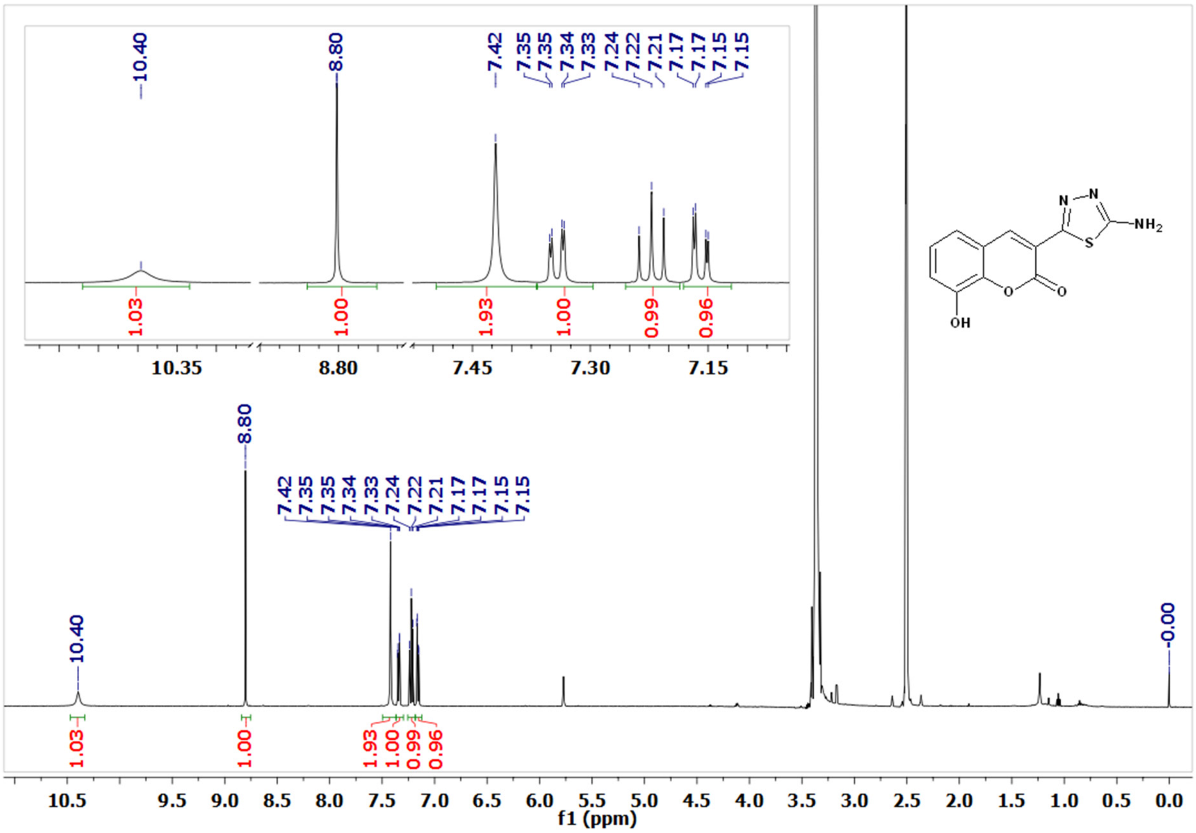

Figure S20. ^{1}H-NMR ($DMSO_{d6}$) spectrum of coumarin-thiadiazole hybrid **11.**

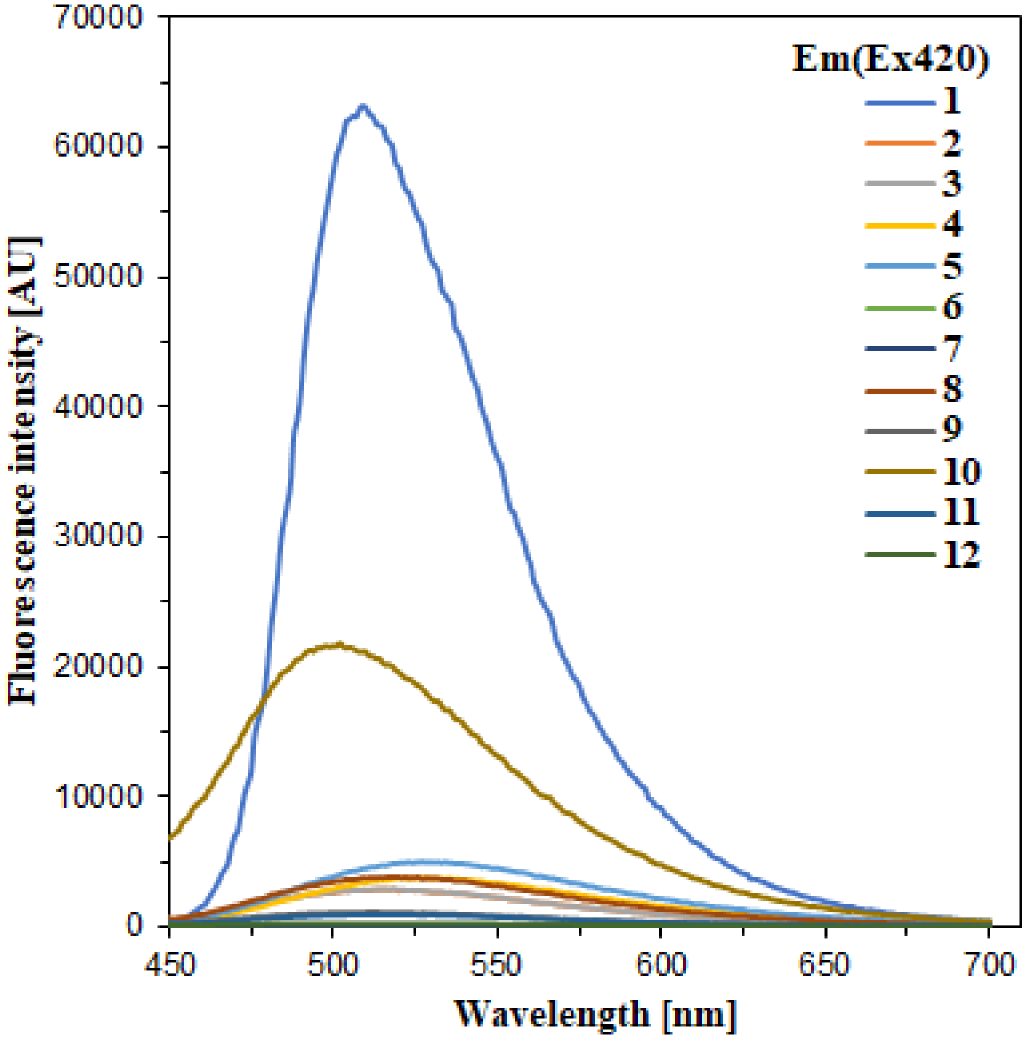

Figure S21. Fluorescence emission spectra of coumarin-thiadiazole hybrids **1-12** in methanol. The spectra were recorded at the λ_{ex}= 420 nm and concentration of 0.01 mM, except **1** which was recorded at the concentration of 0.002 mM.
