# Supplementary material for: Design, Spectroscopy, and Assessment of Cholinesterase Inhibition and Antimicrobial Activities of Novel Coumarin–Thiadiazole Hybrids
Source: Int J Mol Sci. 2022 Jun 5;23(11):6314. doi: 10.3390/ijms23116314 (PMC9180949; doi:10.3390/ijms23116314)
Supplement: Supplementary file 1 [file ijms-23-06314-s001.zip › Supplementary materials - Crystallographic data.zip!/Supplementary materials - Crystallographic data/11'/11'_tables.html]

11'


# 11'

Table 1 Crystal data and structure refinement for 11'.

| Identification code | 11' |
| Empirical formula | C_12_H_8_O_6.42_ |
| Formula weight | 255.36 |
| Temperature/K | 293(2) |
| Crystal system | orthorhombic |
| Space group | Pna2_1_ |
| a/Å | 21.1129(7) |
| b/Å | 5.1623(2) |
| c/Å | 23.0980(11) |
| α/° | 90 |
| β/° | 90 |
| γ/° | 90 |
| Volume/Å^3^ | 2517.48(18) |
| Z | 8 |
| ρ_calc_g/cm^3^ | 1.348 |
| μ/mm^‑1^ | 0.967 |
| F(000) | 1053.0 |
| Crystal size/mm^3^ | 0.3 × 0.1 × 0.1 |
| Radiation | CuKα (λ = 1.54184) |
| 2Θ range for data collection/° | 7.654 to 152.736 |
| Index ranges | -26 ≤ h ≤ 23, -6 ≤ k ≤ 5, -25 ≤ l ≤ 28 |
| Reflections collected | 5812 |
| Independent reflections | 3745 [R_int_ = 0.0190, R_sigma_ = 0.0286] |
| Data/restraints/parameters | 3745/1/342 |
| Goodness-of-fit on F^2^ | 1.058 |
| Final R indexes [I>=2σ (I)] | R_1_ = 0.0679, wR_2_ = 0.1980 |
| Final R indexes [all data] | R_1_ = 0.0774, wR_2_ = 0.2134 |
| Largest diff. peak/hole / e Å^-3^ | 0.83/-0.26 || Flack parameter | 0.21(18) |

Table 2 Fractional Atomic Coordinates (×10^4^) and Equivalent Isotropic Displacement Parameters (Å^2^×10^3^) for 11'. U_eq_ is defined as 1/3 of of the trace of the orthogonalised U_IJ_ tensor.

| Atom | *x* | *y* | *z* | U(eq) |
| --- | --- | --- | --- | --- |
| O1 | -2788(13) | -11380(20) | -4001(9) | 316(13) |
| O1B | -5042.4(19) | -3770(8) | -3173.5(19) | 60.1(10) |
| O2B | -4619(3) | -6053(15) | -3886(2) | 108(2) |
| O3B | -5775(2) | 314(8) | -2904(2) | 63.5(11) |
| O4B | -6535(3) | -2456(10) | -3140(3) | 79.6(14) |
| O5B | -3723(2) | -11024(9) | -2772(3) | 75.0(13) |
| C10B | -4892(2) | -4886(10) | -2178(3) | 49.5(12) |
| C2B | -4639(3) | -5731(13) | -3363(3) | 64.8(16) |
| C3B | -4320(3) | -7214(11) | -2919(3) | 53.8(12) |
| C4B | -4452(3) | -6819(10) | -2353(3) | 54.4(13) |
| C5B | -5025(3) | -4321(14) | -1595(3) | 62.3(15) |
| C6B | -5433(3) | -2293(13) | -1467(3) | 64.4(15) |
| C7B | -5695(3) | -836(11) | -1889(3) | 60.7(15) |
| C8B | -5564(2) | -1309(10) | -2465(3) | 53.0(13) |
| C9B | -5158(2) | -3378(10) | -2615(3) | 47.2(11) |
| C11B | -3839(3) | -9214(13) | -3068(3) | 63.3(16) |
| C12B | -6248(3) | -493(12) | -3228(3) | 63.0(15) |
| C13B | -6363(5) | 1386(17) | -3724(4) | 99(3) |
| O6B | -3525(3) | -8665(14) | -3565(3) | 94(3) |
| O1A | -2301.0(19) | -11116(8) | -5594.3(17) | 56.0(9) |
| O2A | -1879(3) | -8886(13) | -4907(2) | 104(2) |
| O3A | -3013.5(19) | -15266(8) | -5882(2) | 62.4(11) |
| O4A | -3769(3) | -12461(11) | -5644(3) | 86.7(16) |
| O5A | -980(3) | -3872(10) | -6037(2) | 76.4(13) |
| O6A | -856(4) | -5804(15) | -5209(3) | 121(3) |
| C2A | -1909(3) | -9169(13) | -5419(3) | 63.0(15) |
| C3A | -1582(3) | -7637(12) | -5866(3) | 53.6(12) |
| C4A | -1707(2) | -8035(10) | -6421(3) | 50.3(12) |
| C5A | -2265(3) | -10629(11) | -7187(3) | 54.3(13) |
| C6A | -2677(3) | -12647(12) | -7325(3) | 62.9(15) |
| C7A | -2936(3) | -14161(11) | -6885(3) | 57.2(14) |
| C8A | -2806(3) | -13612(10) | -6324(3) | 51.3(13) |
| C9A | -2415(2) | -11571(10) | -6186(3) | 51.0(12) |
| C10A | -2135(2) | -10064(11) | -6607(3) | 51.2(12) |
| C11A | -1119(3) | -5533(13) | -5704(3) | 63.3(16) |
| C12A | -3498(3) | -14400(12) | -5533(3) | 62.7(14) |
| C13A | -3603(5) | -16234(17) | -5061(4) | 101(3) |

Table 3 Anisotropic Displacement Parameters (Å^2^×10^3^) for 11'. The Anisotropic displacement factor exponent takes the form: -2π^2^[h^2^a\*^2^U_11_+2hka\*b\*U_12_+…].

| Atom | U_11_ | U_22_ | U_33_ | U_23_ | U_13_ | U_12_ |
| --- | --- | --- | --- | --- | --- | --- |
| O1 | 560(40) | 144(9) | 241(17) | 32(11) | 40(20) | 70(16) |
| O1B | 53(2) | 66(2) | 61(2) | 10(2) | 1.5(19) | 12.4(19) |
| O2B | 116(4) | 147(6) | 62(3) | 12(4) | 19(3) | 54(4) |
| O3B | 60(2) | 52(2) | 79(3) | 17(2) | -8(2) | 2.5(18) |
| O4B | 73(3) | 83(3) | 84(3) | -4(3) | -15(3) | 1(3) |
| O5B | 73(3) | 56(2) | 96(4) | 2(3) | -5(3) | 23(2) |
| C10B | 45(2) | 44(2) | 59(3) | 4(2) | 1(2) | -7(2) |
| C2B | 62(3) | 68(3) | 64(4) | 11(3) | 8(3) | 14(3) |
| C3B | 46(2) | 47(3) | 68(3) | 7(3) | 4(3) | 2(2) |
| C4B | 50(3) | 44(2) | 69(4) | 8(3) | -5(3) | 0(2) |
| C5B | 58(3) | 71(4) | 57(4) | -1(3) | -2(3) | -2(3) |
| C6B | 66(3) | 66(3) | 61(4) | -10(3) | 1(3) | 1(3) |
| C7B | 50(2) | 49(3) | 84(4) | -6(3) | -2(3) | -1(2) |
| C8B | 41(2) | 40(2) | 78(4) | 7(3) | -2(3) | 2.4(19) |
| C9B | 41(2) | 46(2) | 54(3) | 7(2) | -3(2) | -3(2) |
| C11B | 53(3) | 59(3) | 78(4) | -5(3) | -3(3) | 3(3) |
| C12B | 60(3) | 54(3) | 75(4) | -3(3) | -2(3) | 14(3) |
| C13B | 106(6) | 113(7) | 79(5) | 17(5) | -3(5) | 36(6) |
| O6B | 85(4) | 106(5) | 91(5) | 27(4) | 32(4) | 42(4) |
| O1A | 58(2) | 59(2) | 51(2) | 2.2(18) | 1.6(18) | -12.1(17) |
| O2A | 127(5) | 130(5) | 57(3) | -5(3) | 2(3) | -67(4) |
| O3A | 54(2) | 48.7(19) | 84(3) | 2(2) | 9(2) | -6.7(16) |
| O4A | 79(3) | 78(3) | 103(4) | -3(3) | 28(3) | 19(3) |
| O5A | 70(3) | 70(3) | 89(3) | 7(3) | -2(3) | -16(2) |
| O6A | 149(6) | 139(6) | 75(3) | 20(4) | -44(4) | -85(5) |
| C2A | 62(3) | 70(4) | 57(4) | 5(3) | -4(3) | -18(3) |
| C3A | 46(3) | 56(3) | 59(3) | -3(3) | -3(3) | -8(2) |
| C4A | 40(2) | 49(2) | 61(3) | 6(2) | 5(2) | -2(2) |
| C5A | 57(3) | 52(3) | 53(3) | 1(2) | 1(2) | -3(2) |
| C6A | 61(3) | 65(3) | 62(3) | -6(3) | -10(3) | 4(3) |
| C7A | 50(2) | 51(3) | 70(4) | -2(3) | -10(3) | -6(2) |
| C8A | 49(2) | 43(2) | 62(3) | 4(2) | 4(3) | -1(2) |
| C9A | 45(2) | 49(3) | 59(3) | 2(3) | -1(2) | -1(2) |
| C10A | 45(2) | 51(2) | 58(3) | 2(2) | 2(2) | -1(2) |
| C11A | 59(3) | 59(3) | 72(4) | 6(3) | -1(3) | -18(3) |
| C12A | 59(3) | 61(3) | 68(4) | -7(3) | 6(3) | -4(3) |
| C13A | 124(7) | 87(5) | 91(6) | 12(5) | 33(6) | -2(5) |

Table 4 Bond Lengths for 11'.

| Atom | Atom | Length/Å |  | Atom | Atom | Length/Å |
| --- | --- | --- | --- | --- | --- | --- |
| O1B | C2B | 1.394(8) |  | O1A | C2A | 1.364(7) |
| O1B | C9B | 1.329(8) |  | O1A | C9A | 1.408(7) |
| O2B | C2B | 1.221(9) |  | O2A | C2A | 1.193(8) |
| O3B | C8B | 1.389(7) |  | O3A | C8A | 1.400(7) |
| O3B | C12B | 1.317(8) |  | O3A | C12A | 1.377(8) |
| O4B | C12B | 1.198(8) |  | O4A | C12A | 1.181(9) |
| O5B | C11B | 1.183(9) |  | O5A | C11A | 1.189(8) |
| C10B | C4B | 1.421(8) |  | O6A | C11A | 1.278(9) |
| C10B | C5B | 1.406(9) |  | C2A | C3A | 1.472(9) |
| C10B | C9B | 1.393(8) |  | C3A | C4A | 1.326(9) |
| C2B | C3B | 1.446(9) |  | C3A | C11A | 1.508(8) |
| C3B | C4B | 1.353(9) |  | C4A | C10A | 1.448(7) |
| C3B | C11B | 1.489(8) |  | C5A | C6A | 1.393(8) |
| C5B | C6B | 1.386(9) |  | C5A | C10A | 1.399(8) |
| C6B | C7B | 1.349(10) |  | C6A | C7A | 1.394(10) |
| C7B | C8B | 1.381(10) |  | C7A | C8A | 1.356(9) |
| C8B | C9B | 1.412(7) |  | C8A | C9A | 1.375(7) |
| C11B | O6B | 1.356(10) |  | C9A | C10A | 1.379(8) |
| C12B | C13B | 1.520(10) |  | C12A | C13A | 1.462(11) |

Table 5 Bond Angles for 11'.

| Atom | Atom | Atom | Angle/˚ |  | Atom | Atom | Atom | Angle/˚ |
| --- | --- | --- | --- | --- | --- | --- | --- | --- |
| C9B | O1B | C2B | 121.9(5) |  | C2A | O1A | C9A | 121.0(4) |
| C12B | O3B | C8B | 117.9(5) |  | C12A | O3A | C8A | 117.5(5) |
| C5B | C10B | C4B | 123.3(6) |  | O1A | C2A | C3A | 118.2(5) |
| C9B | C10B | C4B | 116.8(5) |  | O2A | C2A | O1A | 114.7(6) |
| C9B | C10B | C5B | 119.7(5) |  | O2A | C2A | C3A | 127.1(6) |
| O1B | C2B | C3B | 116.5(6) |  | C2A | C3A | C11A | 121.1(5) |
| O2B | C2B | O1B | 115.5(6) |  | C4A | C3A | C2A | 120.1(5) |
| O2B | C2B | C3B | 127.9(6) |  | C4A | C3A | C11A | 118.7(5) |
| C2B | C3B | C11B | 121.4(6) |  | C3A | C4A | C10A | 121.6(5) |
| C4B | C3B | C2B | 120.7(5) |  | C6A | C5A | C10A | 119.8(6) |
| C4B | C3B | C11B | 117.9(5) |  | C5A | C6A | C7A | 119.9(6) |
| C3B | C4B | C10B | 121.0(5) |  | C8A | C7A | C6A | 120.0(5) |
| C6B | C5B | C10B | 119.0(6) |  | C7A | C8A | O3A | 120.3(5) |
| C7B | C6B | C5B | 121.5(6) |  | C7A | C8A | C9A | 120.2(6) |
| C6B | C7B | C8B | 121.0(6) |  | C9A | C8A | O3A | 119.2(6) |
| O3B | C8B | C9B | 118.2(6) |  | C8A | C9A | O1A | 117.0(5) |
| C7B | C8B | O3B | 122.2(5) |  | C8A | C9A | C10A | 121.8(6) |
| C7B | C8B | C9B | 119.5(5) |  | C10A | C9A | O1A | 121.1(5) |
| O1B | C9B | C10B | 122.9(5) |  | C5A | C10A | C4A | 123.9(5) |
| O1B | C9B | C8B | 117.7(5) |  | C9A | C10A | C4A | 117.8(5) |
| C10B | C9B | C8B | 119.3(6) |  | C9A | C10A | C5A | 118.2(5) |
| O5B | C11B | C3B | 123.7(7) |  | O5A | C11A | O6A | 123.5(6) |
| O5B | C11B | O6B | 123.6(6) |  | O5A | C11A | C3A | 121.2(6) |
| O6B | C11B | C3B | 112.6(6) |  | O6A | C11A | C3A | 115.1(6) |
| O3B | C12B | C13B | 110.4(6) |  | O3A | C12A | C13A | 109.9(6) |
| O4B | C12B | O3B | 123.6(7) |  | O4A | C12A | O3A | 120.6(7) |
| O4B | C12B | C13B | 126.0(7) |  | O4A | C12A | C13A | 129.6(7) |

Table 6 Torsion Angles for 11'.

| A | B | C | D | Angle/˚ |  | A | B | C | D | Angle/˚ |
| --- | --- | --- | --- | --- | --- | --- | --- | --- | --- | --- |
| O1B | C2B | C3B | C4B | 5.0(9) |  | O1A | C2A | C3A | C4A | 5.0(9) |
| O1B | C2B | C3B | C11B | -175.0(5) |  | O1A | C2A | C3A | C11A | -177.2(6) |
| O2B | C2B | C3B | C4B | -171.6(8) |  | O1A | C9A | C10A | C4A | 1.5(7) |
| O2B | C2B | C3B | C11B | 8.4(12) |  | O1A | C9A | C10A | C5A | 179.6(5) |
| O3B | C8B | C9B | O1B | -5.1(7) |  | O2A | C2A | C3A | C4A | -173.9(8) |
| O3B | C8B | C9B | C10B | 174.0(5) |  | O2A | C2A | C3A | C11A | 3.8(12) |
| C10B | C5B | C6B | C7B | -0.9(10) |  | O3A | C8A | C9A | O1A | -6.5(7) |
| C2B | O1B | C9B | C10B | -0.3(8) |  | O3A | C8A | C9A | C10A | 172.2(5) |
| C2B | O1B | C9B | C8B | 178.7(5) |  | C2A | O1A | C9A | C8A | 178.6(5) |
| C2B | C3B | C4B | C10B | -2.0(9) |  | C2A | O1A | C9A | C10A | 0.0(8) |
| C2B | C3B | C11B | O5B | -153.4(7) |  | C2A | C3A | C4A | C10A | -3.7(9) |
| C2B | C3B | C11B | O6B | 29.7(9) |  | C2A | C3A | C11A | O5A | -160.2(7) |
| C4B | C10B | C5B | C6B | 176.1(5) |  | C2A | C3A | C11A | O6A | 25.2(10) |
| C4B | C10B | C9B | O1B | 3.5(7) |  | C3A | C4A | C10A | C5A | -177.6(6) |
| C4B | C10B | C9B | C8B | -175.6(4) |  | C3A | C4A | C10A | C9A | 0.5(8) |
| C4B | C3B | C11B | O5B | 26.6(9) |  | C4A | C3A | C11A | O5A | 17.6(10) |
| C4B | C3B | C11B | O6B | -150.2(7) |  | C4A | C3A | C11A | O6A | -157.0(8) |
| C5B | C10B | C4B | C3B | -177.2(6) |  | C5A | C6A | C7A | C8A | 2.3(9) |
| C5B | C10B | C9B | O1B | 178.6(5) |  | C6A | C5A | C10A | C4A | 179.0(5) |
| C5B | C10B | C9B | C8B | -0.4(7) |  | C6A | C5A | C10A | C9A | 0.9(8) |
| C5B | C6B | C7B | C8B | -0.3(10) |  | C6A | C7A | C8A | O3A | -173.8(5) |
| C6B | C7B | C8B | O3B | -173.4(5) |  | C6A | C7A | C8A | C9A | -0.3(9) |
| C6B | C7B | C8B | C9B | 1.2(9) |  | C7A | C8A | C9A | O1A | 180.0(5) |
| C7B | C8B | C9B | O1B | -179.9(5) |  | C7A | C8A | C9A | C10A | -1.4(8) |
| C7B | C8B | C9B | C10B | -0.8(7) |  | C8A | O3A | C12A | O4A | 7.9(9) |
| C8B | O3B | C12B | O4B | 5.6(9) |  | C8A | O3A | C12A | C13A | -173.6(6) |
| C8B | O3B | C12B | C13B | -173.5(6) |  | C8A | C9A | C10A | C4A | -177.1(5) |
| C9B | O1B | C2B | O2B | 173.2(7) |  | C8A | C9A | C10A | C5A | 1.1(8) |
| C9B | O1B | C2B | C3B | -3.9(9) |  | C9A | O1A | C2A | O2A | 176.0(7) |
| C9B | C10B | C4B | C3B | -2.2(8) |  | C9A | O1A | C2A | C3A | -3.1(9) |
| C9B | C10B | C5B | C6B | 1.3(9) |  | C10A | C5A | C6A | C7A | -2.6(9) |
| C11B | C3B | C4B | C10B | 178.0(5) |  | C11A | C3A | C4A | C10A | 178.5(5) |
| C12B | O3B | C8B | C7B | -105.8(7) |  | C12A | O3A | C8A | C7A | -108.6(6) |
| C12B | O3B | C8B | C9B | 79.6(6) |  | C12A | O3A | C8A | C9A | 77.9(7) |

Table 7 Hydrogen Atom Coordinates (Å×10^4^) and Isotropic Displacement Parameters (Å^2^×10^3^) for 11'.

| Atom | *x* | *y* | *z* | U(eq) |
| --- | --- | --- | --- | --- |
| H1 | -3011(14) | -10130(60) | -3687(14) | -1(6) |
| H4B | -4252.35 | -7827.77 | -2073.57 | 65 |
| H5B | -4843.46 | -5292.41 | -1299.72 | 75 |
| H6B | -5527.26 | -1928.53 | -1082.69 | 77 |
| H7B | -5967.84 | 506.2 | -1789.95 | 73 |
| H13A | -6572.78 | 501.15 | -4035.14 | 149 |
| H13B | -5964.41 | 2053.03 | -3858.72 | 149 |
| H13C | -6622.81 | 2791.76 | -3591.89 | 149 |
| H6A | -784.37 | -7343.25 | -5150.4 | 182 |
| H4A | -1514.93 | -6987.55 | -6697.77 | 60 |
| H5A | -2077.83 | -9660.02 | -7480.18 | 65 |
| H6AA | -2777.79 | -12982.91 | -7709.44 | 75 |
| H7A | -3198.93 | -15547.49 | -6976.55 | 69 |
| H13D | -3345.67 | -15757.16 | -4735.02 | 151 |
| H13E | -3490.84 | -17946.59 | -5186.23 | 151 |
| H13F | -4041.31 | -16203.01 | -4950.41 | 151 |

Table 8 Atomic Occupancy for 11'.

| Atom | *Occupancy* |  | Atom | *Occupancy* |  | Atom | *Occupancy* |
| --- | --- | --- | --- | --- | --- | --- | --- |
| O6B | 0.847(18) |  |  |  |  |  |  |

Table 9 Solvent masks information for 11'.

| Number | X | Y | Z | Volume | Electron count | Content |
| --- | --- | --- | --- | --- | --- | --- |
| 1 | 0.002 | 0.259 | 0.035 | 56.6 | 13.5 | ? |
| 2 | -0.002 | -0.259 | 0.535 | 56.6 | 13.5 | ? |
| 3 | 0.502 | 0.241 | 0.035 | 56.6 | 13.5 | ? |
| 4 | 0.498 | 0.759 | 0.535 | 56.6 | 13.5 | ? |

11'


# 11'

Table 1 Crystal data and structure refinement for 11'.

| Identification code | 11' |
| Empirical formula | C_12_H_8_O_6.42_ |
| Formula weight | 255.36 |
| Temperature/K | 293(2) |
| Crystal system | orthorhombic |
| Space group | Pna2_1_ |
| a/Å | 21.1129(7) |
| b/Å | 5.1623(2) |
| c/Å | 23.0980(11) |
| α/° | 90 |
| β/° | 90 |
| γ/° | 90 |
| Volume/Å^3^ | 2517.48(18) |
| Z | 8 |
| ρ_calc_g/cm^3^ | 1.348 |
| μ/mm^‑1^ | 0.967 |
| F(000) | 1053.0 |
| Crystal size/mm^3^ | 0.3 × 0.1 × 0.1 |
| Radiation | CuKα (λ = 1.54184) |
| 2Θ range for data collection/° | 7.654 to 152.736 |
| Index ranges | -26 ≤ h ≤ 23, -6 ≤ k ≤ 5, -25 ≤ l ≤ 28 |
| Reflections collected | 5812 |
| Independent reflections | 3745 [R_int_ = 0.0190, R_sigma_ = 0.0286] |
| Data/restraints/parameters | 3745/1/342 |
| Goodness-of-fit on F^2^ | 1.058 |
| Final R indexes [I>=2σ (I)] | R_1_ = 0.0679, wR_2_ = 0.1980 |
| Final R indexes [all data] | R_1_ = 0.0774, wR_2_ = 0.2134 |
| Largest diff. peak/hole / e Å^-3^ | 0.83/-0.26 || Flack parameter | 0.21(18) |
