# Supplementary material for: Design, Spectroscopy, and Assessment of Cholinesterase Inhibition and Antimicrobial Activities of Novel Coumarin–Thiadiazole Hybrids
Source: Int J Mol Sci. 2022 Jun 5;23(11):6314. doi: 10.3390/ijms23116314 (PMC9180949; doi:10.3390/ijms23116314)
Supplement: Supplementary file 1 [file ijms-23-06314-s001.zip › Supplementary materials - Crystallographic data.zip!/Supplementary materials - Crystallographic data/11/11_tables.html]

11


# 11

Table 1 Crystal data and structure refinement for 11.

| Identification code | 11 |
| Empirical formula | C_11_H_9_N_3_O_4_S |
| Formula weight | 279.27 |
| Temperature/K | 294.37(10) |
| Crystal system | monoclinic |
| Space group | Pn |
| a/Å | 6.7057(2) |
| b/Å | 4.7543(2) |
| c/Å | 17.7050(6) |
| α/° | 90 |
| β/° | 95.298(3) |
| γ/° | 90 |
| Volume/Å^3^ | 562.04(3) |
| Z | 2 |
| ρ_calc_g/cm^3^ | 1.650 |
| μ/mm^‑1^ | 2.740 |
| F(000) | 288.0 |
| Crystal size/mm^3^ | 0.3 × 0.2 × 0.02 |
| Radiation | Cu Kα (λ = 1.54184) |
| 2Θ range for data collection/° | 10.034 to 152.168 |
| Index ranges | -8 ≤ h ≤ 7, -5 ≤ k ≤ 5, -21 ≤ l ≤ 19 |
| Reflections collected | 1983 |
| Independent reflections | 1499 [R_int_ = 0.0259, R_sigma_ = 0.0518] |
| Data/restraints/parameters | 1499/2/176 |
| Goodness-of-fit on F^2^ | 1.049 |
| Final R indexes [I>=2σ (I)] | R_1_ = 0.0343, wR_2_ = 0.0815 |
| Final R indexes [all data] | R_1_ = 0.0358, wR_2_ = 0.0833 |
| Largest diff. peak/hole / e Å^-3^ | 0.28/-0.19 || Flack parameter | -0.02(3) |

Table 2 Fractional Atomic Coordinates (×10^4^) and Equivalent Isotropic Displacement Parameters (Å^2^×10^3^) for 11. U_eq_ is defined as 1/3 of of the trace of the orthogonalised U_IJ_ tensor.

| Atom | *x* | *y* | *z* | U(eq) |
| --- | --- | --- | --- | --- |
| S11 | 5963.3(10) | 3490.3(19) | 4122.7(6) | 32.7(2) |
| O1 | 6092(4) | 9890(6) | 5984.0(16) | 34.2(6) |
| O2 | 7518(4) | 6811(7) | 5279.0(19) | 46.1(8) |
| O3 | 6644(4) | 13645(7) | 7086.5(17) | 41.6(7) |
| N14 | 2275(5) | 4989(7) | 3941.3(18) | 34.2(7) |
| O4 | 9026(5) | -778(8) | 3466(2) | 52.9(8) |
| N16 | 4943(6) | 120(8) | 2917(2) | 42.0(8) |
| C2 | 5996(5) | 7972(8) | 5410(2) | 32.9(7) |
| N13 | 2503(5) | 3037(7) | 3380.0(19) | 35.7(7) |
| C8 | 4755(5) | 13383(8) | 6739(2) | 31.9(7) |
| C3 | 4024(5) | 7484(8) | 4993(2) | 30.2(7) |
| C12 | 4335(5) | 2041(8) | 3404(2) | 32.3(8) |
| C10 | 2568(5) | 10964(8) | 5779(2) | 30.9(7) |
| C9 | 4459(5) | 11393(8) | 6162(2) | 30.4(7) |
| C15 | 3914(5) | 5473(8) | 4368(2) | 29.1(7) |
| C6 | 1247(6) | 14559(9) | 6545(2) | 37.1(8) |
| C4 | 2406(5) | 8886(8) | 5191(2) | 32.2(7) |
| C7 | 3139(6) | 14958(9) | 6922(2) | 37.0(8) |
| C5 | 955(6) | 12587(9) | 5981(2) | 35.4(8) |

Table 3 Anisotropic Displacement Parameters (Å^2^×10^3^) for 11. The Anisotropic displacement factor exponent takes the form: -2π^2^[h^2^a\*^2^U_11_+2hka\*b\*U_12_+…].

| Atom | U_11_ | U_22_ | U_33_ | U_23_ | U_13_ | U_12_ |
| --- | --- | --- | --- | --- | --- | --- |
| S11 | 29.1(4) | 34.7(4) | 33.6(4) | -3.1(4) | -1.4(3) | 0.0(4) |
| O1 | 26.7(12) | 38.6(15) | 36.2(13) | -7.5(12) | -2.8(10) | 1.5(10) |
| O2 | 32.0(14) | 52.6(18) | 52.3(18) | -15.2(15) | -3.6(13) | 7.7(13) |
| O3 | 36.7(15) | 50.3(17) | 35.8(14) | -10.9(13) | -7.0(12) | 0.7(12) |
| N14 | 34.9(16) | 33.7(16) | 32.8(15) | -3.9(14) | -3.2(13) | 3.0(12) |
| O4 | 38.5(16) | 54(2) | 65(2) | -0.2(18) | -2.6(15) | 0.0(14) |
| N16 | 43.3(18) | 42.1(17) | 39.8(17) | -10.9(17) | 0.2(14) | 0.8(15) |
| C2 | 29.6(17) | 35.2(18) | 33.4(17) | -0.6(15) | -0.4(14) | 1.3(14) |
| N13 | 36.1(16) | 38.4(17) | 31.2(15) | -4.1(14) | -4.4(12) | 1.5(13) |
| C8 | 29.8(17) | 38.1(19) | 27.1(16) | 2.3(15) | -0.9(13) | -4.3(15) |
| C3 | 31.5(17) | 30.4(18) | 27.9(15) | 2.9(14) | -1.6(13) | -3.2(13) |
| C12 | 34.5(18) | 32.5(18) | 29.2(17) | 1.4(15) | -0.1(14) | -3.6(14) |
| C10 | 29.8(17) | 31.8(18) | 30.6(17) | 3.5(14) | 0.4(13) | -1.2(13) |
| C9 | 28.8(16) | 32.2(18) | 29.8(16) | 3.3(14) | -0.2(13) | -0.6(13) |
| C15 | 28.4(16) | 28.2(16) | 30.2(16) | 6.1(14) | -0.4(13) | 3.1(13) |
| C6 | 35.6(19) | 38(2) | 38.5(19) | -1.4(16) | 7.2(15) | 4.3(15) |
| C4 | 28.1(16) | 36.3(19) | 31.4(17) | 1.4(15) | -1.5(13) | -2.6(14) |
| C7 | 41(2) | 37.8(19) | 32.2(18) | -1.8(16) | 4.1(15) | 0.7(17) |
| C5 | 27.4(17) | 42(2) | 36.7(18) | 3.1(17) | 2.1(14) | 1.8(14) |

Table 4 Bond Lengths for 11.

| Atom | Atom | Length/Å |  | Atom | Atom | Length/Å |
| --- | --- | --- | --- | --- | --- | --- |
| S11 | C12 | 1.740(4) |  | N13 | C12 | 1.313(5) |
| S11 | C15 | 1.754(4) |  | C8 | C9 | 1.393(5) |
| O1 | C2 | 1.363(5) |  | C8 | C7 | 1.380(6) |
| O1 | C9 | 1.368(5) |  | C3 | C15 | 1.459(5) |
| O2 | C2 | 1.202(5) |  | C3 | C4 | 1.347(5) |
| O3 | C8 | 1.362(5) |  | C10 | C9 | 1.396(5) |
| N14 | N13 | 1.378(5) |  | C10 | C4 | 1.432(5) |
| N14 | C15 | 1.296(5) |  | C10 | C5 | 1.402(5) |
| N16 | C12 | 1.345(5) |  | C6 | C7 | 1.391(6) |
| C2 | C3 | 1.472(5) |  | C6 | C5 | 1.370(6) |

Table 5 Bond Angles for 11.

| Atom | Atom | Atom | Angle/˚ |  | Atom | Atom | Atom | Angle/˚ |
| --- | --- | --- | --- | --- | --- | --- | --- | --- |
| C12 | S11 | C15 | 86.60(18) |  | N13 | C12 | N16 | 124.2(4) |
| C2 | O1 | C9 | 122.5(3) |  | C9 | C10 | C4 | 117.3(3) |
| C15 | N14 | N13 | 113.3(3) |  | C9 | C10 | C5 | 118.9(4) |
| O1 | C2 | C3 | 117.2(3) |  | C5 | C10 | C4 | 123.8(3) |
| O2 | C2 | O1 | 117.9(3) |  | O1 | C9 | C8 | 117.4(3) |
| O2 | C2 | C3 | 124.8(4) |  | O1 | C9 | C10 | 121.5(3) |
| C12 | N13 | N14 | 112.6(3) |  | C8 | C9 | C10 | 121.1(4) |
| O3 | C8 | C9 | 117.2(3) |  | N14 | C15 | S11 | 113.7(3) |
| O3 | C8 | C7 | 124.2(4) |  | N14 | C15 | C3 | 122.7(3) |
| C7 | C8 | C9 | 118.6(3) |  | C3 | C15 | S11 | 123.6(3) |
| C15 | C3 | C2 | 117.6(3) |  | C5 | C6 | C7 | 120.4(4) |
| C4 | C3 | C2 | 120.0(4) |  | C3 | C4 | C10 | 121.4(3) |
| C4 | C3 | C15 | 122.4(3) |  | C8 | C7 | C6 | 120.9(4) |
| N16 | C12 | S11 | 122.0(3) |  | C6 | C5 | C10 | 120.1(4) |
| N13 | C12 | S11 | 113.8(3) |  |  |  |  |  |

Table 6 Torsion Angles for 11.

| A | B | C | D | Angle/˚ |  | A | B | C | D | Angle/˚ |
| --- | --- | --- | --- | --- | --- | --- | --- | --- | --- | --- |
| O1 | C2 | C3 | C15 | 178.8(3) |  | C9 | C8 | C7 | C6 | 0.5(6) |
| O1 | C2 | C3 | C4 | -0.7(5) |  | C9 | C10 | C4 | C3 | -2.6(5) |
| O2 | C2 | C3 | C15 | -1.0(6) |  | C9 | C10 | C5 | C6 | 0.1(6) |
| O2 | C2 | C3 | C4 | 179.5(4) |  | C15 | S11 | C12 | N16 | 179.3(4) |
| O3 | C8 | C9 | O1 | -0.8(5) |  | C15 | S11 | C12 | N13 | 0.5(3) |
| O3 | C8 | C9 | C10 | 179.4(3) |  | C15 | N14 | N13 | C12 | 1.1(5) |
| O3 | C8 | C7 | C6 | -179.6(4) |  | C15 | C3 | C4 | C10 | -176.5(3) |
| N14 | N13 | C12 | S11 | -1.0(4) |  | C4 | C3 | C15 | S11 | -176.9(3) |
| N14 | N13 | C12 | N16 | -179.7(4) |  | C4 | C3 | C15 | N14 | 3.0(6) |
| C2 | O1 | C9 | C8 | -177.3(3) |  | C4 | C10 | C9 | O1 | -0.1(5) |
| C2 | O1 | C9 | C10 | 2.5(6) |  | C4 | C10 | C9 | C8 | 179.7(4) |
| C2 | C3 | C15 | S11 | 3.6(5) |  | C4 | C10 | C5 | C6 | -179.1(4) |
| C2 | C3 | C15 | N14 | -176.5(4) |  | C7 | C8 | C9 | O1 | 179.1(4) |
| C2 | C3 | C4 | C10 | 3.0(6) |  | C7 | C8 | C9 | C10 | -0.7(6) |
| N13 | N14 | C15 | S11 | -0.7(4) |  | C7 | C6 | C5 | C10 | -0.3(6) |
| N13 | N14 | C15 | C3 | 179.4(3) |  | C5 | C10 | C9 | O1 | -179.4(3) |
| C12 | S11 | C15 | N14 | 0.1(3) |  | C5 | C10 | C9 | C8 | 0.4(6) |
| C12 | S11 | C15 | C3 | -180.0(3) |  | C5 | C10 | C4 | C3 | 176.6(4) |
| C9 | O1 | C2 | O2 | 177.8(4) |  | C5 | C6 | C7 | C8 | 0.0(6) |
| C9 | O1 | C2 | C3 | -2.1(5) |  |  |  |  |  |  |

Table 7 Hydrogen Atom Coordinates (Å×10^4^) and Isotropic Displacement Parameters (Å^2^×10^3^) for 11.

| Atom | *x* | *y* | *z* | U(eq) |
| --- | --- | --- | --- | --- |
| H3 | 6665.96 | 14904.25 | 7403.53 | 62 |
| H4A | 9477.74 | -2263.32 | 3687.1 | 79 |
| H4B | 9995.18 | 206.58 | 3335.49 | 79 |
| H16A | 4111.17 | -532.99 | 2561.48 | 50 |
| H16B | 6164.65 | -453.99 | 2961.38 | 50 |
| H6 | 174.65 | 15638.28 | 6675.39 | 45 |
| H4 | 1156.71 | 8497.19 | 4940.18 | 39 |
| H7 | 3317.97 | 16304.09 | 7303.33 | 44 |
| H5 | -314.44 | 12323.82 | 5732.79 | 43 |

11


# 11

Table 1 Crystal data and structure refinement for 11.

| Identification code | 11 |
| Empirical formula | C_11_H_9_N_3_O_4_S |
| Formula weight | 279.27 |
| Temperature/K | 294.37(10) |
| Crystal system | monoclinic |
| Space group | Pn |
| a/Å | 6.7057(2) |
| b/Å | 4.7543(2) |
| c/Å | 17.7050(6) |
| α/° | 90 |
| β/° | 95.298(3) |
| γ/° | 90 |
| Volume/Å^3^ | 562.04(3) |
| Z | 2 |
| ρ_calc_g/cm^3^ | 1.650 |
| μ/mm^‑1^ | 2.740 |
| F(000) | 288.0 |
| Crystal size/mm^3^ | 0.3 × 0.2 × 0.02 |
| Radiation | Cu Kα (λ = 1.54184) |
| 2Θ range for data collection/° | 10.034 to 152.168 |
| Index ranges | -8 ≤ h ≤ 7, -5 ≤ k ≤ 5, -21 ≤ l ≤ 19 |
| Reflections collected | 1983 |
| Independent reflections | 1499 [R_int_ = 0.0259, R_sigma_ = 0.0518] |
| Data/restraints/parameters | 1499/2/176 |
| Goodness-of-fit on F^2^ | 1.049 |
| Final R indexes [I>=2σ (I)] | R_1_ = 0.0343, wR_2_ = 0.0815 |
| Final R indexes [all data] | R_1_ = 0.0358, wR_2_ = 0.0833 |
| Largest diff. peak/hole / e Å^-3^ | 0.28/-0.19 || Flack parameter | -0.02(3) |
