# Supplementary material for: Design, Spectroscopy, and Assessment of Cholinesterase Inhibition and Antimicrobial Activities of Novel Coumarin–Thiadiazole Hybrids
Source: Int J Mol Sci. 2022 Jun 5;23(11):6314. doi: 10.3390/ijms23116314 (PMC9180949; doi:10.3390/ijms23116314)
Supplement: Supplementary file 1 [file ijms-23-06314-s001.zip › Supplementary materials - Crystallographic data.zip!/Supplementary materials - Crystallographic data/4'/4`_tables.html]

4`


# 4`

Table 1 Crystal data and structure refinement for 4`.

|  |  |
| --- | --- |
| Identification code | 4` |
| Empirical formula | C_10_H_5_ClO_4_ |
| Formula weight | 224.59 |
| Temperature/K | 296 |
| Crystal system | monoclinic |
| Space group | P2_1_/c |
| a/Å | 8.8456(3) |
| b/Å | 10.9181(3) |
| c/Å | 9.4257(3) |
| α/° | 90 |
| β/° | 90.398(3) |
| γ/° | 90 |
| Volume/Å^3^ | 910.29(5) |
| Z | 4 |
| ρ_calc_g/cm^3^ | 1.639 |
| μ/mm^‑1^ | 3.673 |
| F(000) | 456.0 |
| Crystal size/mm^3^ | 0.3 × 0.2 × 0.2 |
| Radiation | Cu Kα (λ = 1.54184) |
| 2Θ range for data collection/° | 10 to 152.238 |
| Index ranges | -10 ≤ h ≤ 10, -11 ≤ k ≤ 13, -11 ≤ l ≤ 8 |
| Reflections collected | 3222 |
| Independent reflections | 1827 [R_int_ = 0.0212, R_sigma_ = 0.0235] |
| Data/restraints/parameters | 1827/0/137 |
| Goodness-of-fit on F^2^ | 1.062 |
| Final R indexes [I>=2σ (I)] | R_1_ = 0.0516, wR_2_ = 0.1333 |
| Final R indexes [all data] | R_1_ = 0.0597, wR_2_ = 0.1442 |
| Largest diff. peak/hole / e Å^-3^ | 0.42/-0.44 |

Table 2 Fractional Atomic Coordinates (×10^4^) and Equivalent Isotropic Displacement Parameters (Å^2^×10^3^) for 4`. U_eq_ is defined as 1/3 of of the trace of the orthogonalised U_IJ_ tensor.

| Atom | *x* | *y* | *z* | U(eq) |
| --- | --- | --- | --- | --- |
| Cl1 | 1815.6(8) | 1593.7(5) | 5514.7(8) | 65.0(3) |
| O1 | 1610.0(18) | 6915.4(15) | 6202.4(18) | 52.3(4) |
| O2 | 2257(2) | 8796.0(17) | 5672(2) | 68.8(5) |
| O3 | 4984(2) | 7664(2) | 2385(2) | 74.2(6) |
| O4 | 4270(2) | 9232.8(19) | 3683(3) | 76.3(6) |
| C9 | 1694(2) | 5671.8(19) | 6004(2) | 43.5(4) |
| C10 | 2586(2) | 5164(2) | 4937(2) | 42.9(4) |
| C4 | 3420(2) | 5996(2) | 4056(2) | 47.2(5) |
| C3 | 3360(2) | 7211(2) | 4283(2) | 47.5(5) |
| C5 | 2614(2) | 3892(2) | 4772(2) | 47.2(5) |
| C2 | 2413(3) | 7723(2) | 5395(3) | 50.6(5) |
| C8 | 856(3) | 4944(2) | 6914(2) | 51.9(5) |
| C6 | 1786(2) | 3178(2) | 5679(2) | 47.0(5) |
| C7 | 906(3) | 3689(2) | 6748(2) | 51.5(5) |
| C11 | 4270(3) | 8054(3) | 3373(3) | 56.7(6) |

Table 3 Anisotropic Displacement Parameters (Å^2^×10^3^) for 4`. The Anisotropic displacement factor exponent takes the form: -2π^2^[h^2^a\*^2^U_11_+2hka\*b\*U_12_+…].

| Atom | U_11_ | U_22_ | U_33_ | U_23_ | U_13_ | U_12_ |
| --- | --- | --- | --- | --- | --- | --- |
| Cl1 | 76.6(4) | 41.8(3) | 76.8(4) | -0.7(2) | 14.4(3) | -1.7(2) |
| O1 | 55.0(8) | 44.7(8) | 57.3(9) | -4.0(6) | 17.3(7) | 1.8(6) |
| O2 | 74.1(11) | 44.6(9) | 87.9(14) | 1.5(8) | 14.3(10) | 4.0(8) |
| O3 | 75.6(12) | 84.7(14) | 62.5(11) | 10.8(10) | 18.4(9) | -26.2(10) |
| O4 | 79.2(13) | 56.7(11) | 93.3(15) | 15.5(10) | 12.2(11) | -12.8(9) |
| C9 | 41.6(10) | 42.3(10) | 46.8(10) | -2.2(8) | 9.0(8) | 2.2(8) |
| C10 | 41.3(9) | 46.2(10) | 41.4(9) | 2.0(8) | 8.7(8) | 1.8(8) |
| C4 | 43.5(10) | 55.6(12) | 42.5(10) | 2.0(8) | 10.4(8) | -0.5(9) |
| C3 | 44.9(10) | 51.4(11) | 46.4(11) | 6.1(9) | 2.4(9) | -4.2(9) |
| C5 | 46.1(10) | 48.3(11) | 47.3(11) | -3.2(8) | 11.7(8) | 5.0(8) |
| C2 | 51.1(11) | 42.6(11) | 58.0(12) | 3.3(9) | 4.7(9) | 1.9(8) |
| C8 | 51.5(11) | 54.8(12) | 49.8(11) | -3.0(9) | 18.2(9) | 0.1(9) |
| C6 | 47.8(11) | 42.3(10) | 51.0(11) | 0.9(8) | 6.8(9) | 0.8(8) |
| C7 | 51.5(11) | 52.0(12) | 51.1(11) | 3.6(9) | 14.6(9) | -4.5(9) |
| C11 | 51.6(12) | 64.0(14) | 54.5(12) | 14.2(11) | 0.3(10) | -11.8(11) |

Table 4 Bond Lengths for 4`.

| Atom | Atom | Length/Å |  | Atom | Atom | Length/Å |
| --- | --- | --- | --- | --- | --- | --- |
| Cl1 | C6 | 1.737(2) |  | C10 | C4 | 1.438(3) |
| O1 | C9 | 1.373(3) |  | C10 | C5 | 1.398(3) |
| O1 | C2 | 1.367(3) |  | C4 | C3 | 1.344(3) |
| O2 | C2 | 1.208(3) |  | C3 | C2 | 1.458(3) |
| O3 | C11 | 1.206(4) |  | C3 | C11 | 1.496(3) |
| O4 | C11 | 1.320(4) |  | C5 | C6 | 1.372(3) |
| C9 | C10 | 1.397(3) |  | C8 | C7 | 1.380(3) |
| C9 | C8 | 1.388(3) |  | C6 | C7 | 1.394(3) |

Table 5 Bond Angles for 4`.

| Atom | Atom | Atom | Angle/˚ |  | Atom | Atom | Atom | Angle/˚ |
| --- | --- | --- | --- | --- | --- | --- | --- | --- |
| C2 | O1 | C9 | 122.25(17) |  | O1 | C2 | C3 | 117.1(2) |
| O1 | C9 | C10 | 121.45(19) |  | O2 | C2 | O1 | 116.4(2) |
| O1 | C9 | C8 | 116.92(18) |  | O2 | C2 | C3 | 126.5(2) |
| C8 | C9 | C10 | 121.6(2) |  | C7 | C8 | C9 | 118.7(2) |
| C9 | C10 | C4 | 117.4(2) |  | C5 | C6 | Cl1 | 120.11(17) |
| C9 | C10 | C5 | 119.02(19) |  | C5 | C6 | C7 | 121.7(2) |
| C5 | C10 | C4 | 123.62(19) |  | C7 | C6 | Cl1 | 118.16(17) |
| C3 | C4 | C10 | 120.7(2) |  | C8 | C7 | C6 | 119.9(2) |
| C4 | C3 | C2 | 121.1(2) |  | O3 | C11 | O4 | 121.0(2) |
| C4 | C3 | C11 | 119.6(2) |  | O3 | C11 | C3 | 120.8(3) |
| C2 | C3 | C11 | 119.3(2) |  | O4 | C11 | C3 | 118.1(2) |
| C6 | C5 | C10 | 118.99(19) |  |  |  |  |  |

Table 6 Torsion Angles for 4`.

| A | B | C | D | Angle/˚ |  | A | B | C | D | Angle/˚ |
| --- | --- | --- | --- | --- | --- | --- | --- | --- | --- | --- |
| Cl1 | C6 | C7 | C8 | 179.90(19) |  | C4 | C3 | C2 | O1 | 0.8(3) |
| O1 | C9 | C10 | C4 | 0.3(3) |  | C4 | C3 | C2 | O2 | -178.6(2) |
| O1 | C9 | C10 | C5 | -179.20(19) |  | C4 | C3 | C11 | O3 | 4.6(3) |
| O1 | C9 | C8 | C7 | 179.9(2) |  | C4 | C3 | C11 | O4 | -175.4(2) |
| C9 | O1 | C2 | O2 | -179.6(2) |  | C5 | C10 | C4 | C3 | -179.1(2) |
| C9 | O1 | C2 | C3 | 1.0(3) |  | C5 | C6 | C7 | C8 | -0.1(4) |
| C9 | C10 | C4 | C3 | 1.5(3) |  | C2 | O1 | C9 | C10 | -1.5(3) |
| C9 | C10 | C5 | C6 | -1.2(3) |  | C2 | O1 | C9 | C8 | 178.4(2) |
| C9 | C8 | C7 | C6 | -0.1(4) |  | C2 | C3 | C11 | O3 | -175.0(2) |
| C10 | C9 | C8 | C7 | -0.3(3) |  | C2 | C3 | C11 | O4 | 5.0(3) |
| C10 | C4 | C3 | C2 | -2.0(3) |  | C8 | C9 | C10 | C4 | -179.6(2) |
| C10 | C4 | C3 | C11 | 178.43(19) |  | C8 | C9 | C10 | C5 | 1.0(3) |
| C10 | C5 | C6 | Cl1 | -179.24(16) |  | C11 | C3 | C2 | O1 | -179.6(2) |
| C10 | C5 | C6 | C7 | 0.8(3) |  | C11 | C3 | C2 | O2 | 1.0(4) |
| C4 | C10 | C5 | C6 | 179.4(2) |  |  |  |  |  |  |

Table 7 Hydrogen Atom Coordinates (Å×10^4^) and Isotropic Displacement Parameters (Å^2^×10^3^) for 4`.

| Atom | *x* | *y* | *z* | U(eq) |
| --- | --- | --- | --- | --- |
| H4 | 3647.51 | 9366.6 | 4305.31 | 115 |
| H4A | 4006.45 | 5691.1 | 3321.73 | 57 |
| H5 | 3184.97 | 3534.29 | 4058.14 | 57 |
| H8 | 270.9 | 5295.33 | 7622.26 | 62 |
| H7 | 354.67 | 3185.25 | 7348.41 | 62 |

4`


# 4`

Table 1 Crystal data and structure refinement for 4`.

|  |  |
| --- | --- |
| Identification code | 4` |
| Empirical formula | C_10_H_5_ClO_4_ |
| Formula weight | 224.59 |
| Temperature/K | 296 |
| Crystal system | monoclinic |
| Space group | P2_1_/c |
| a/Å | 8.8456(3) |
| b/Å | 10.9181(3) |
| c/Å | 9.4257(3) |
| α/° | 90 |
| β/° | 90.398(3) |
| γ/° | 90 |
| Volume/Å^3^ | 910.29(5) |
| Z | 4 |
| ρ_calc_g/cm^3^ | 1.639 |
| μ/mm^‑1^ | 3.673 |
| F(000) | 456.0 |
| Crystal size/mm^3^ | 0.3 × 0.2 × 0.2 |
| Radiation | Cu Kα (λ = 1.54184) |
| 2Θ range for data collection/° | 10 to 152.238 |
| Index ranges | -10 ≤ h ≤ 10, -11 ≤ k ≤ 13, -11 ≤ l ≤ 8 |
| Reflections collected | 3222 |
| Independent reflections | 1827 [R_int_ = 0.0212, R_sigma_ = 0.0235] |
| Data/restraints/parameters | 1827/0/137 |
| Goodness-of-fit on F^2^ | 1.062 |
| Final R indexes [I>=2σ (I)] | R_1_ = 0.0516, wR_2_ = 0.1333 |
| Final R indexes [all data] | R_1_ = 0.0597, wR_2_ = 0.1442 |
| Largest diff. peak/hole / e Å^-3^ | 0.42/-0.44 |
