# Supplementary material for: Design, Spectroscopy, and Assessment of Cholinesterase Inhibition and Antimicrobial Activities of Novel Coumarin–Thiadiazole Hybrids
Source: Int J Mol Sci. 2022 Jun 5;23(11):6314. doi: 10.3390/ijms23116314 (PMC9180949; doi:10.3390/ijms23116314)
Supplement: Supplementary file 1 [file ijms-23-06314-s001.zip › Supplementary materials - Crystallographic data.zip!/Supplementary materials - Crystallographic data/4/4_tables.html]

4


# 4

Table 1 Crystal data and structure refinement for 4.

|  |  |
| --- | --- |
| Identification code | 4 |
| Empirical formula | C_11_H_6.5_ClN_3_O_3_S |
| Formula weight | 296.20 |
| Temperature/K | 295.3(9) |
| Crystal system | triclinic |
| Space group | P-1 |
| a/Å | 4.9572(2) |
| b/Å | 16.7370(5) |
| c/Å | 17.3370(7) |
| α/° | 109.745(3) |
| β/° | 90.661(3) |
| γ/° | 94.347(3) |
| Volume/Å^3^ | 1348.90(9) |
| Z | 4 |
| ρ_calc_g/cm^3^ | 1.459 |
| μ/mm^‑1^ | 4.043 |
| F(000) | 602.0 |
| Crystal size/mm^3^ | 0.3 × 0.05 × 0.04 |
| Radiation | CuKα (λ = 1.54184) |
| 2Θ range for data collection/° | 17.96 to 152.482 |
| Index ranges | -6 ≤ h ≤ 6, -20 ≤ k ≤ 16, -19 ≤ l ≤ 21 |
| Reflections collected | 6909 |
| Independent reflections | 4344 [R_int_ = 0.2129, R_sigma_ = 0.1163] |
| Data/restraints/parameters | 4344/0/401 |
| Goodness-of-fit on F^2^ | 1.000 |
| Final R indexes [I>=2σ (I)] | R_1_ = 0.0941, wR_2_ = 0.2386 |
| Final R indexes [all data] | R_1_ = 0.1474, wR_2_ = 0.2575 |
| Largest diff. peak/hole / e Å^-3^ | 0.89/-0.55 |

Table 2 Fractional Atomic Coordinates (×10^4^) and Equivalent Isotropic Displacement Parameters (Å^2^×10^3^) for 4. U_eq_ is defined as 1/3 of of the trace of the orthogonalised U_IJ_ tensor.

| Atom | *x* | *y* | *z* | U(eq) |
| --- | --- | --- | --- | --- |
| Cl1A | 9460(3) | 5656.5(12) | 9342.3(9) | 78.9(5) |
| O1A | 1283(7) | 6279(2) | 7193(2) | 55.4(8) |
| O2A | -1866(7) | 5849(2) | 6204(2) | 58.9(8) |
| C2A | -70(9) | 5648(3) | 6556(3) | 46.6(9) |
| C9A | 3278(9) | 6127(3) | 7673(3) | 49.2(9) |
| C8A | 4490(12) | 6803(3) | 8310(4) | 64.3(13) |
| C7A | 6431(12) | 6672(4) | 8815(4) | 65.7(13) |
| C6A | 7122(10) | 5837(4) | 8677(3) | 56.2(11) |
| C5A | 5977(10) | 5165(3) | 8044(3) | 52.6(10) |
| C10A | 3984(8) | 5292(3) | 7516(3) | 44.6(9) |
| C4A | 2699(9) | 4628(3) | 6838(3) | 45.7(9) |
| S11A | -3369(2) | 4172.2(7) | 5116.0(7) | 48.5(3) |
| N14A | 461(8) | 3352(2) | 5388(2) | 49.8(8) |
| N13A | -1066(7) | 2777(2) | 4745(3) | 48.7(8) |
| N16A | -4962(8) | 2667(3) | 3931(3) | 58.4(10) |
| C3A | 794(8) | 4783(3) | 6358(3) | 42.2(8) |
| C15A | -491(8) | 4105(3) | 5655(2) | 41.7(8) |
| C12A | -3149(8) | 3114(3) | 4548(3) | 45.8(9) |
| Cl1B | 16572(4) | 1198.5(18) | 9531.6(13) | 107.5(7) |
| O1B | 10927(8) | 3152(2) | 7897(3) | 66.3(9) |
| O2B | 7739(9) | 3300(2) | 7077(3) | 72.1(10) |
| C2B | 8812(11) | 2811(3) | 7328(3) | 56.5(11) |
| C9B | 12242(11) | 2671(4) | 8247(3) | 61.2(12) |
| C8B | 14326(12) | 3078(4) | 8826(4) | 73.0(16) |
| C7B | 15649(13) | 2616(5) | 9209(4) | 81.8(19) |
| C6B | 14931(12) | 1749(5) | 9016(4) | 71.0(15) |
| C5B | 12903(11) | 1336(4) | 8439(3) | 62.6(12) |
| C10B | 11530(10) | 1797(3) | 8048(3) | 53.7(10) |
| C4B | 9420(9) | 1424(3) | 7428(3) | 50.8(10) |
| C3B | 8093(9) | 1895(3) | 7085(3) | 47.9(9) |
| S11B | 4290(2) | 2116.6(7) | 5952.8(8) | 54.3(3) |
| N14B | 4897(8) | 745(2) | 6258(2) | 51.0(8) |
| N13B | 2850(8) | 535(2) | 5679(3) | 52.6(9) |
| N16B | 386(10) | 1129(3) | 4878(3) | 63.5(11) |
| C15B | 5886(9) | 1542(3) | 6468(3) | 45.9(9) |
| C12B | 2293(10) | 1180(3) | 5444(3) | 52.6(10) |
| O2 | -178(12) | -756(4) | 7475(4) | 51.4(14) |
| O1 | 4865(10) | -736(3) | 6752(3) | 78.8(12) |
| O3 | -440(30) | -1408(10) | 7837(10) | 67(4) |
| O4 | -400(60) | -1083(16) | 8707(15) | 118(9) |

Table 3 Anisotropic Displacement Parameters (Å^2^×10^3^) for 4. The Anisotropic displacement factor exponent takes the form: -2π^2^[h^2^a\*^2^U_11_+2hka\*b\*U_12_+…].

| Atom | U_11_ | U_22_ | U_33_ | U_23_ | U_13_ | U_12_ |
| --- | --- | --- | --- | --- | --- | --- |
| Cl1A | 77.4(9) | 79.6(11) | 70.9(8) | 16.6(7) | -21.7(6) | -1.8(7) |
| O1A | 65(2) | 31.7(16) | 68.3(19) | 15.0(14) | 0.6(14) | 5.5(13) |
| O2A | 60(2) | 44.8(19) | 79(2) | 27.6(16) | 1.6(15) | 16.6(15) |
| C2A | 46(2) | 35(2) | 60(2) | 17.0(18) | 8.9(16) | 4.9(17) |
| C9A | 56(3) | 33(2) | 58(2) | 14.9(18) | 5.2(17) | -0.3(17) |
| C8A | 77(4) | 34(3) | 73(3) | 8(2) | 3(2) | -4(2) |
| C7A | 75(3) | 45(3) | 64(3) | 5(2) | -1(2) | -10(2) |
| C6A | 55(3) | 52(3) | 56(2) | 14(2) | -1.6(17) | -6(2) |
| C5A | 54(2) | 44(2) | 58(2) | 14.6(19) | -1.7(17) | 0.3(19) |
| C10A | 45(2) | 33(2) | 53(2) | 11.7(16) | 1.8(15) | -2.2(16) |
| C4A | 47(2) | 31(2) | 57(2) | 12.5(17) | 2.3(15) | -1.4(16) |
| S11A | 42.8(6) | 38.0(6) | 64.4(6) | 15.8(4) | -2.9(4) | 9.5(4) |
| N14A | 48.5(19) | 32.2(18) | 67(2) | 15.0(16) | -9.4(14) | 3.7(14) |
| N13A | 41.9(18) | 33.8(18) | 69(2) | 15.8(16) | -5.0(14) | 1.0(14) |
| N16A | 44(2) | 47(2) | 78(3) | 13.7(19) | -12.2(16) | 4.1(16) |
| C3A | 44(2) | 27(2) | 56(2) | 16.0(16) | 4.8(15) | 0.5(15) |
| C15A | 40.0(19) | 33(2) | 55(2) | 18.6(17) | 1.1(14) | 3.7(15) |
| C12A | 35.9(19) | 41(2) | 65(2) | 23.2(19) | 2.3(15) | 3.0(16) |
| Cl1B | 94.3(13) | 135(2) | 97.1(12) | 45.1(12) | -25.3(9) | 10.6(12) |
| O1B | 75(2) | 36.0(18) | 78(2) | 8.4(16) | 6.9(17) | -9.3(16) |
| O2B | 84(3) | 37(2) | 98(3) | 25.8(19) | 8(2) | 5.1(17) |
| C2B | 60(3) | 36(2) | 72(3) | 17(2) | 14(2) | 0.2(19) |
| C9B | 60(3) | 47(3) | 62(3) | 2(2) | 9(2) | -9(2) |
| C8B | 68(3) | 62(4) | 70(3) | 2(3) | 3(2) | -17(3) |
| C7B | 69(4) | 89(5) | 64(3) | 0(3) | 2(2) | -16(3) |
| C6B | 64(3) | 84(4) | 62(3) | 21(3) | 3(2) | 1(3) |
| C5B | 56(3) | 61(3) | 65(3) | 16(2) | 0(2) | -3(2) |
| C10B | 50(2) | 46(3) | 60(2) | 13(2) | 8.6(17) | -2.7(19) |
| C4B | 47(2) | 34(2) | 68(3) | 13.1(19) | 3.6(17) | -4.2(17) |
| C3B | 47(2) | 32(2) | 61(2) | 13.2(18) | 12.9(17) | -2.1(17) |
| S11B | 55.5(7) | 37.1(6) | 77.8(7) | 28.8(5) | 6.3(5) | 5.0(4) |
| N14B | 59(2) | 34.2(19) | 61(2) | 16.5(16) | 0.2(15) | 4.6(16) |
| N13B | 59(2) | 31.5(19) | 68(2) | 17.9(16) | -2.6(16) | 3.9(15) |
| N16B | 68(3) | 41(2) | 86(3) | 28(2) | -15(2) | 3(2) |
| C15B | 49(2) | 31(2) | 61(2) | 19.6(17) | 9.0(16) | 5.5(16) |
| C12B | 52(2) | 42(2) | 68(3) | 24(2) | 10.7(18) | 11.8(19) |
| O2 | 42(3) | 50(4) | 75(4) | 40(3) | -8(2) | -8(2) |
| O1 | 80(3) | 58(3) | 110(3) | 43(2) | -1(2) | 9(2) |
| O3 | 90(10) | 45(8) | 82(9) | 47(7) | -25(7) | -13(7) |
| O4 | 210(30) | 76(15) | 88(14) | 55(12) | -32(14) | -2(14) |

Table 4 Bond Lengths for 4.

| Atom | Atom | Length/Å |  | Atom | Atom | Length/Å |
| --- | --- | --- | --- | --- | --- | --- |
| Cl1A | C6A | 1.739(5) |  | Cl1B | C6B | 1.722(7) |
| O1A | C2A | 1.368(6) |  | O1B | C2B | 1.380(7) |
| O1A | C9A | 1.377(6) |  | O1B | C9B | 1.357(8) |
| O2A | C2A | 1.204(6) |  | O2B | C2B | 1.202(7) |
| C2A | C3A | 1.469(6) |  | C2B | C3B | 1.460(6) |
| C9A | C8A | 1.378(7) |  | C9B | C8B | 1.395(8) |
| C9A | C10A | 1.403(6) |  | C9B | C10B | 1.401(7) |
| C8A | C7A | 1.372(9) |  | C8B | C7B | 1.372(11) |
| C7A | C6A | 1.405(8) |  | C7B | C6B | 1.391(11) |
| C6A | C5A | 1.358(7) |  | C6B | C5B | 1.376(8) |
| C5A | C10A | 1.415(6) |  | C5B | C10B | 1.392(8) |
| C10A | C4A | 1.420(6) |  | C10B | C4B | 1.436(7) |
| C4A | C3A | 1.348(6) |  | C4B | C3B | 1.339(7) |
| S11A | C15A | 1.728(4) |  | C3B | C15B | 1.462(6) |
| S11A | C12A | 1.723(5) |  | S11B | C15B | 1.740(4) |
| N14A | N13A | 1.374(5) |  | S11B | C12B | 1.740(6) |
| N14A | C15A | 1.314(5) |  | N14B | N13B | 1.359(6) |
| N13A | C12A | 1.308(5) |  | N14B | C15B | 1.312(6) |
| N16A | C12A | 1.360(6) |  | N13B | C12B | 1.322(6) |
| C3A | C15A | 1.455(6) |  | N16B | C12B | 1.332(7) |

Table 5 Bond Angles for 4.

| Atom | Atom | Atom | Angle/˚ |  | Atom | Atom | Atom | Angle/˚ |
| --- | --- | --- | --- | --- | --- | --- | --- | --- |
| C2A | O1A | C9A | 123.0(3) |  | C9B | O1B | C2B | 122.1(4) |
| O1A | C2A | C3A | 117.3(4) |  | O1B | C2B | C3B | 117.4(5) |
| O2A | C2A | O1A | 117.2(4) |  | O2B | C2B | O1B | 116.5(5) |
| O2A | C2A | C3A | 125.5(4) |  | O2B | C2B | C3B | 126.1(5) |
| O1A | C9A | C8A | 118.7(4) |  | O1B | C9B | C8B | 117.8(5) |
| O1A | C9A | C10A | 119.7(4) |  | O1B | C9B | C10B | 121.7(5) |
| C8A | C9A | C10A | 121.7(5) |  | C8B | C9B | C10B | 120.5(6) |
| C7A | C8A | C9A | 120.3(5) |  | C7B | C8B | C9B | 119.3(6) |
| C8A | C7A | C6A | 118.7(5) |  | C8B | C7B | C6B | 120.2(6) |
| C7A | C6A | Cl1A | 119.2(4) |  | C7B | C6B | Cl1B | 118.8(5) |
| C5A | C6A | Cl1A | 119.1(4) |  | C5B | C6B | Cl1B | 120.1(6) |
| C5A | C6A | C7A | 121.7(5) |  | C5B | C6B | C7B | 121.1(6) |
| C6A | C5A | C10A | 120.1(5) |  | C6B | C5B | C10B | 119.4(6) |
| C9A | C10A | C5A | 117.5(4) |  | C9B | C10B | C4B | 116.8(5) |
| C9A | C10A | C4A | 118.7(4) |  | C5B | C10B | C9B | 119.5(5) |
| C5A | C10A | C4A | 123.8(4) |  | C5B | C10B | C4B | 123.7(5) |
| C3A | C4A | C10A | 121.4(4) |  | C3B | C4B | C10B | 121.8(5) |
| C12A | S11A | C15A | 86.8(2) |  | C2B | C3B | C15B | 116.6(4) |
| C15A | N14A | N13A | 113.2(3) |  | C4B | C3B | C2B | 120.0(5) |
| C12A | N13A | N14A | 111.6(4) |  | C4B | C3B | C15B | 123.4(4) |
| C4A | C3A | C2A | 119.8(4) |  | C12B | S11B | C15B | 86.9(2) |
| C4A | C3A | C15A | 121.4(4) |  | C15B | N14B | N13B | 113.5(4) |
| C15A | C3A | C2A | 118.8(4) |  | C12B | N13B | N14B | 112.8(4) |
| N14A | C15A | S11A | 113.5(3) |  | C3B | C15B | S11B | 124.6(3) |
| N14A | C15A | C3A | 120.8(4) |  | N14B | C15B | C3B | 121.9(4) |
| C3A | C15A | S11A | 125.6(3) |  | N14B | C15B | S11B | 113.4(3) |
| N13A | C12A | S11A | 114.8(4) |  | N13B | C12B | S11B | 113.4(4) |
| N13A | C12A | N16A | 122.3(4) |  | N13B | C12B | N16B | 123.9(5) |
| N16A | C12A | S11A | 122.8(3) |  | N16B | C12B | S11B | 122.7(4) |

Table 6 Torsion Angles for 4.

| A | B | C | D | Angle/˚ |  | A | B | C | D | Angle/˚ |
| --- | --- | --- | --- | --- | --- | --- | --- | --- | --- | --- |
| Cl1A | C6A | C5A | C10A | 177.2(4) |  | Cl1B | C6B | C5B | C10B | 177.7(4) |
| O1A | C2A | C3A | C4A | 5.2(6) |  | O1B | C2B | C3B | C4B | 1.8(6) |
| O1A | C2A | C3A | C15A | -176.5(4) |  | O1B | C2B | C3B | C15B | -178.7(4) |
| O1A | C9A | C8A | C7A | 178.1(5) |  | O1B | C9B | C8B | C7B | 178.2(5) |
| O1A | C9A | C10A | C5A | -177.8(4) |  | O1B | C9B | C10B | C5B | -178.5(4) |
| O1A | C9A | C10A | C4A | 3.0(6) |  | O1B | C9B | C10B | C4B | 2.8(7) |
| O2A | C2A | C3A | C4A | -175.0(5) |  | O2B | C2B | C3B | C4B | -178.5(5) |
| O2A | C2A | C3A | C15A | 3.4(7) |  | O2B | C2B | C3B | C15B | 1.0(7) |
| C2A | O1A | C9A | C8A | -178.9(4) |  | C2B | O1B | C9B | C8B | -179.0(5) |
| C2A | O1A | C9A | C10A | -0.3(6) |  | C2B | O1B | C9B | C10B | 0.2(7) |
| C2A | C3A | C15A | S11A | -11.6(6) |  | C2B | C3B | C15B | S11B | 5.6(5) |
| C2A | C3A | C15A | N14A | 170.7(4) |  | C2B | C3B | C15B | N14B | -173.5(4) |
| C9A | O1A | C2A | O2A | 176.5(4) |  | C9B | O1B | C2B | O2B | 177.8(5) |
| C9A | O1A | C2A | C3A | -3.7(6) |  | C9B | O1B | C2B | C3B | -2.5(7) |
| C9A | C8A | C7A | C6A | -0.8(9) |  | C9B | C8B | C7B | C6B | 0.4(9) |
| C9A | C10A | C4A | C3A | -1.4(6) |  | C9B | C10B | C4B | C3B | -3.4(7) |
| C8A | C9A | C10A | C5A | 0.6(7) |  | C8B | C9B | C10B | C5B | 0.6(7) |
| C8A | C9A | C10A | C4A | -178.6(4) |  | C8B | C9B | C10B | C4B | -178.1(5) |
| C8A | C7A | C6A | Cl1A | -177.0(4) |  | C8B | C7B | C6B | Cl1B | -178.1(5) |
| C8A | C7A | C6A | C5A | 1.9(9) |  | C8B | C7B | C6B | C5B | 0.4(9) |
| C7A | C6A | C5A | C10A | -1.6(8) |  | C7B | C6B | C5B | C10B | -0.7(8) |
| C6A | C5A | C10A | C9A | 0.4(7) |  | C6B | C5B | C10B | C9B | 0.2(8) |
| C6A | C5A | C10A | C4A | 179.5(4) |  | C6B | C5B | C10B | C4B | 178.9(5) |
| C5A | C10A | C4A | C3A | 179.5(4) |  | C5B | C10B | C4B | C3B | 177.9(5) |
| C10A | C9A | C8A | C7A | -0.4(8) |  | C10B | C9B | C8B | C7B | -0.9(8) |
| C10A | C4A | C3A | C2A | -2.7(6) |  | C10B | C4B | C3B | C2B | 1.2(7) |
| C10A | C4A | C3A | C15A | 179.0(4) |  | C10B | C4B | C3B | C15B | -178.3(4) |
| C4A | C3A | C15A | S11A | 166.8(3) |  | C4B | C3B | C15B | S11B | -174.9(4) |
| C4A | C3A | C15A | N14A | -11.0(6) |  | C4B | C3B | C15B | N14B | 6.0(6) |
| N14A | N13A | C12A | S11A | 1.7(5) |  | N14B | N13B | C12B | S11B | -1.0(5) |
| N14A | N13A | C12A | N16A | -179.7(4) |  | N14B | N13B | C12B | N16B | 179.4(5) |
| N13A | N14A | C15A | S11A | -0.9(5) |  | N13B | N14B | C15B | C3B | 179.7(4) |
| N13A | N14A | C15A | C3A | 177.2(4) |  | N13B | N14B | C15B | S11B | 0.5(5) |
| C15A | S11A | C12A | N13A | -1.8(4) |  | C15B | S11B | C12B | N13B | 1.0(4) |
| C15A | S11A | C12A | N16A | 179.6(4) |  | C15B | S11B | C12B | N16B | -179.3(5) |
| C15A | N14A | N13A | C12A | -0.5(6) |  | C15B | N14B | N13B | C12B | 0.3(6) |
| C12A | S11A | C15A | N14A | 1.4(4) |  | C12B | S11B | C15B | C3B | 179.9(4) |
| C12A | S11A | C15A | C3A | -176.5(4) |  | C12B | S11B | C15B | N14B | -0.8(3) |

Table 7 Hydrogen Atom Coordinates (Å×10^4^) and Isotropic Displacement Parameters (Å^2^×10^3^) for 4.

| Atom | *x* | *y* | *z* | U(eq) |
| --- | --- | --- | --- | --- |
| H8A | 3860(150) | 7430(60) | 8500(50) | 90(20) |
| H7A | 7140(170) | 7090(70) | 9440(60) | 120(30) |
| H4A | 3330(110) | 3900(40) | 6620(40) | 59(15) |
| H16C | -5361.82 | 2170.31 | 3964.1 | 70 |
| H16D | -6408.67 | 2930.71 | 3974.82 | 70 |
| H8B | 14590(130) | 3790(50) | 8850(40) | 73(18) |
| H7B | 18100(170) | 2650(70) | 9530(60) | 120(30) |
| H5B | 12880(170) | 680(70) | 8320(60) | 110(30) |
| H4B | 8850(100) | 870(40) | 7310(30) | 46(12) |
| H1A | 5607.69 | -277.06 | 6712.81 | 118 |
| H1B | 3211.46 | -739.87 | 6613.76 | 118 |
| H16A | -580(120) | 690(40) | 4730(40) | 53(15) |
| H16B | 160(120) | 1660(40) | 4780(40) | 61(15) |

Table 8 Atomic Occupancy for 4.

| Atom | *Occupancy* |  | Atom | *Occupancy* |  | Atom | *Occupancy* |
| --- | --- | --- | --- | --- | --- | --- | --- |
| O2 | 0.5 |  | O3 | 0.25 |  | O4 | 0.25 |

4


# 4

Table 1 Crystal data and structure refinement for 4.

|  |  |
| --- | --- |
| Identification code | 4 |
| Empirical formula | C_11_H_6.5_ClN_3_O_3_S |
| Formula weight | 296.20 |
| Temperature/K | 295.3(9) |
| Crystal system | triclinic |
| Space group | P-1 |
| a/Å | 4.9572(2) |
| b/Å | 16.7370(5) |
| c/Å | 17.3370(7) |
| α/° | 109.745(3) |
| β/° | 90.661(3) |
| γ/° | 94.347(3) |
| Volume/Å^3^ | 1348.90(9) |
| Z | 4 |
| ρ_calc_g/cm^3^ | 1.459 |
| μ/mm^‑1^ | 4.043 |
| F(000) | 602.0 |
| Crystal size/mm^3^ | 0.3 × 0.05 × 0.04 |
| Radiation | CuKα (λ = 1.54184) |
| 2Θ range for data collection/° | 17.96 to 152.482 |
| Index ranges | -6 ≤ h ≤ 6, -20 ≤ k ≤ 16, -19 ≤ l ≤ 21 |
| Reflections collected | 6909 |
| Independent reflections | 4344 [R_int_ = 0.2129, R_sigma_ = 0.1163] |
| Data/restraints/parameters | 4344/0/401 |
| Goodness-of-fit on F^2^ | 1.000 |
| Final R indexes [I>=2σ (I)] | R_1_ = 0.0941, wR_2_ = 0.2386 |
| Final R indexes [all data] | R_1_ = 0.1474, wR_2_ = 0.2575 |
| Largest diff. peak/hole / e Å^-3^ | 0.89/-0.55 |
